# Diastereospecific Synthesis of Vicinally Substituted 2-Oxazolidinones via Oxidative Rearrangement of α,β-Unsaturated γ-Lactams

**DOI:** 10.1021/acs.joc.4c02653

**Published:** 2025-01-06

**Authors:** Anna Lidskog, Yutang Li, Arvind Kumar Gupta, Abhishek Mishra, Anders Sundin, Kenneth Wärnmark

**Affiliations:** Center for Analysis and Synthesis, Department of Chemistry, Lund University, Lund SE 221 00, Sweden

## Abstract

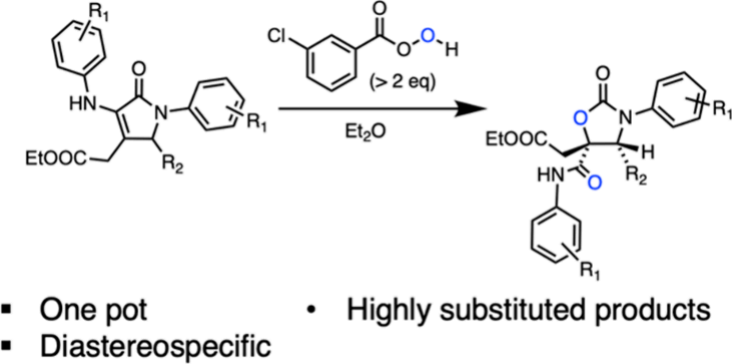

A diastereospecific synthesis of vicinally substituted 2-oxazolidinones from α,β-unsaturated lactams using *m*-chloroperoxybenzoic acid is reported. Several highly substituted 2-oxazolidinones were obtained in 19–46% yields in a one-pot reaction with complete control over the relative stereochemistry. The proposed reaction sequence consists of a Baeyer–Villiger oxidation, an epoxidation, and a concerted rearrangement. Experimental results and density functional theory calculations indicate that a CH_2_COOEt substituent at position 4 of the lactam is necessary for the diastereospecific rearrangement to take place.

2-Oxazolidinones make up a class of five-membered heterocyclic compounds that have found broad applications in both chemistry and medicine.^1^ 2-Oxazolidinones have been employed as effective chiral auxiliaries in a number of different asymmetric reactions, including α-alkylation of enolates, aldol reactions, 1,4-additions, and cycloadditions.^2^ In addition, the oxazolidinone scaffold can be found in biologically active molecules, such as the antibiotics linezolid^3^ and tedizolid^4^ and cytokine modulator (−)-cytoxazone,^5^ the latter containing vicinal substituents of a specific configuration (Figure 1).

**Figure 1:**
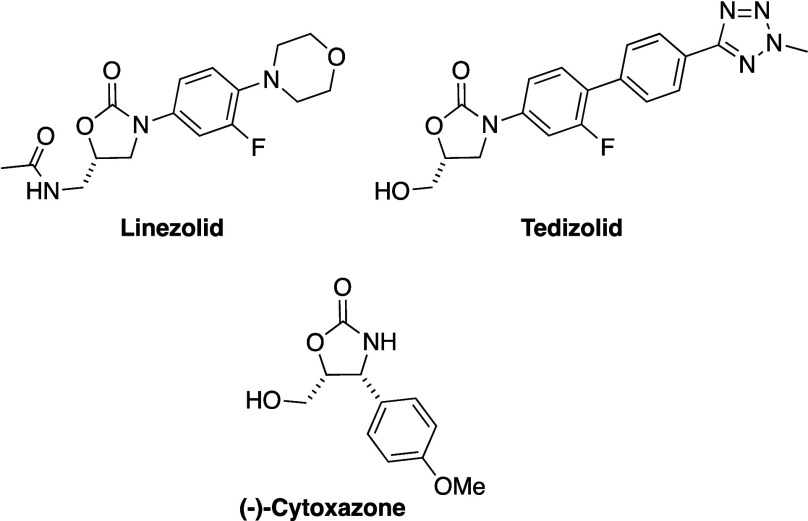
Examples of drugs and bioactive molecules containing 2-oxazolidinones.

Due to their important properties, the synthesis of 2-oxazolidinones has attracted a great deal of attention, with reported synthetic strategies including cycloaddition of CO_2_ to aziridines,^6^ cycloaddition of isocyanates and epoxides,^7^ and cyclization of *N*-Boc propargyl or ethynyl amines.^8^ Diastereo- or enantioselective methods for the synthesis of substituted 2-oxazolidinones are less common, although some notable examples using chiral catalysts or chiral auxiliaries have been reported.^9^ Herein, we report the synthesis of vicinally substituted 2-oxazolidinones through a diastereospecific oxidative rearrangement of α,β-unsaturated γ-lactams using *m*-chloroperoxybenzoic acid (*m*CPBA) (Scheme 1). The starting materials (α,β-unsaturated γ-lactams) are synthetically accessible, with some constraints, by a one-pot three-component reaction developed by Palacios^10^ and modified by Li.^11^

**Scheme 1:**
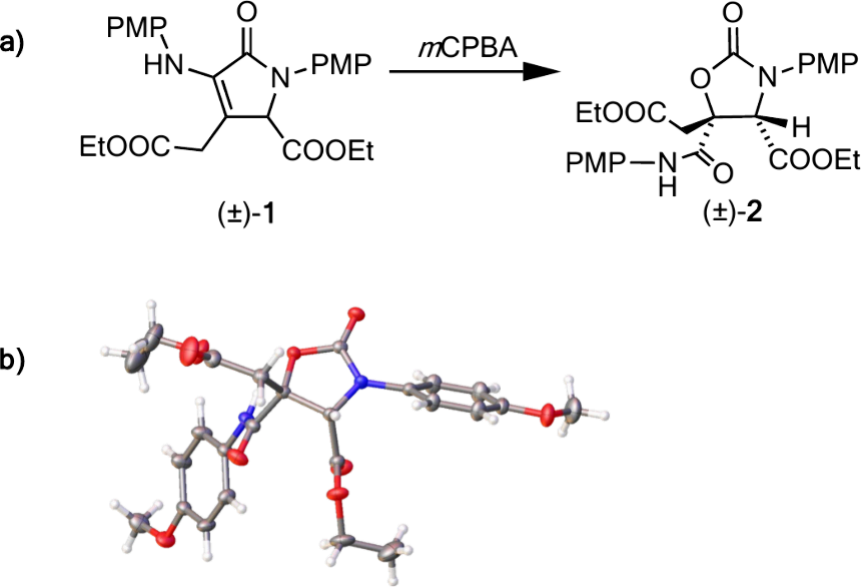
(a) Oxidative Rearrangement of α,β-Unsaturated γ-Lactam (±)-**1** into Oxazolidinone (±)-**2** and (b) Crystal Structure of Oxazolidinone (±)-**2** PMP = *p*-methoxyphenyl. Thermal ellipsoids are shown at the 30% probability level.

The conditions for the oxidative rearrangement were optimized in terms of the number of equivalents of the oxidant, reaction time, temperature, concentration, and solvent (Table S1). As a starting point, α,β-unsaturated γ-lactam (±)-**1** was reacted with 3 equiv of *m*CPBA in CH_2_Cl_2_ at room temperature, which afforded oxazolidinone (±)-**2** in 35% isolated yield. The variation of the number of equivalents of *m*CPBA, the reaction temperature and concentration did not affect or improve the yield. However, changing the solvent from CH_2_Cl_2_ to diethyl ether increased the yield from 35% to 46% (Table S1).

After reaction for 4 h, all of the α,β-unsaturated γ-lactam (±)-**1** was consumed, and prolonging the reaction time further did not increase the yield of the desired product (±)-**2**. In addition to a 46% yield of oxazolidinone product (±)-**2**, one side product (**(±)-3**) was isolated in ∼15% yield. The side product has the same molecular weight as product (±)-**2** and very similar ^1^H and ^13^C nuclear magnetic resonance (NMR) spectra [Figures S48 and S49, compared to Figures S27 and S28 for product (±)-**2**]. While the ^1^H NMR spectrum of product (±)-**2** contains an amide proton resonance at 8.34 ppm (Figure S27), the ^1^H NMR spectrum of side product **3** instead contains a peak at 6.15 ppm (Figure S48). Combined with the observed differences in the chemical shifts of some of the peaks in the ^13^C NMR spectra (Figures S28 and S49), this indicates that the connectivity of the structure of side product **(±)-3** is different from that of the structure of product (±)-**2**. Unfortunately, attempted crystallizations of compound **(±)-3** to obtain crystals suitable for X-ray diffraction (XRD) studies were unsuccessful. However, based on the combined one- and two-dimensional (2D) NMR spectra (Figures S48–S54), a proposed structure of compound (±)-**3** is shown in Figure 2a. A more detailed discussion of this assignment is included in the Supporting Information.

**Figure 2:**
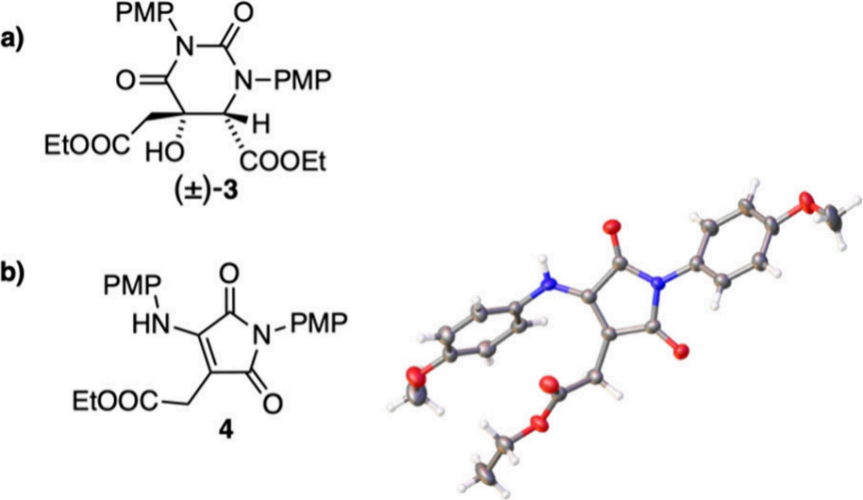
(a) Suggested structure of side product (±)-**3**. (b) Structure of side product **4** (left) and corresponding crystal structure (right). Thermal ellipsoids are shown at the 30% probability level. PMP = *p*-methoxyphenyl.

In addition to product (±)-**2** and side product (±)-**3**, the reaction crude contained decomposed or overoxidized starting material, which could not be isolated or identified. Additionally, it was found that exposure of (±)-**2** to a base during the workup or purification resulted in the hydrolysis of the oxazolidinone to the corresponding aminoalcohol, which reacts further to form maleimide **4** (Figure 2b). The structure of **4** was confirmed by single-crystal XRD.

Using the optimized reaction conditions, a small substrate scope was established using α,β-unsaturated γ-lactams (±)-**1** and (±)-**5**–**9** to yield oxazolidinones (±)-**2** and (±)-**16**–**20**, respectively (Table 1 and Supporting Information). The scope was limited by the availability of the α,β-unsaturated γ-lactams (see the Supporting Information), which were synthesized following a modified protocol previously reported by Palacios and co-workers.^10^ Both electron-donating and -withdrawing substituents on the phenyl rings were tolerated and gave the corresponding oxazolidinones (±)-**2** and (±)-**16**–**18** in 26–46% isolated yields (Table 1). The substituent at position 5 could be exchanged from an ester to a cyclohexyl or *p*-nitrophenyl group, giving the corresponding products (±)-**19** and (±)-**20** in 19% and 33% isolated yields, respectively. Interestingly, when the CH_2_COOEt group at position 4 was replaced with a hydrogen, no oxazolidinone product was observed [compounds (±)-**10**–**15** (Table S3)]. This indicates that the ester group plays an important role in the reaction mechanism (vide infra).

**Table 1:**
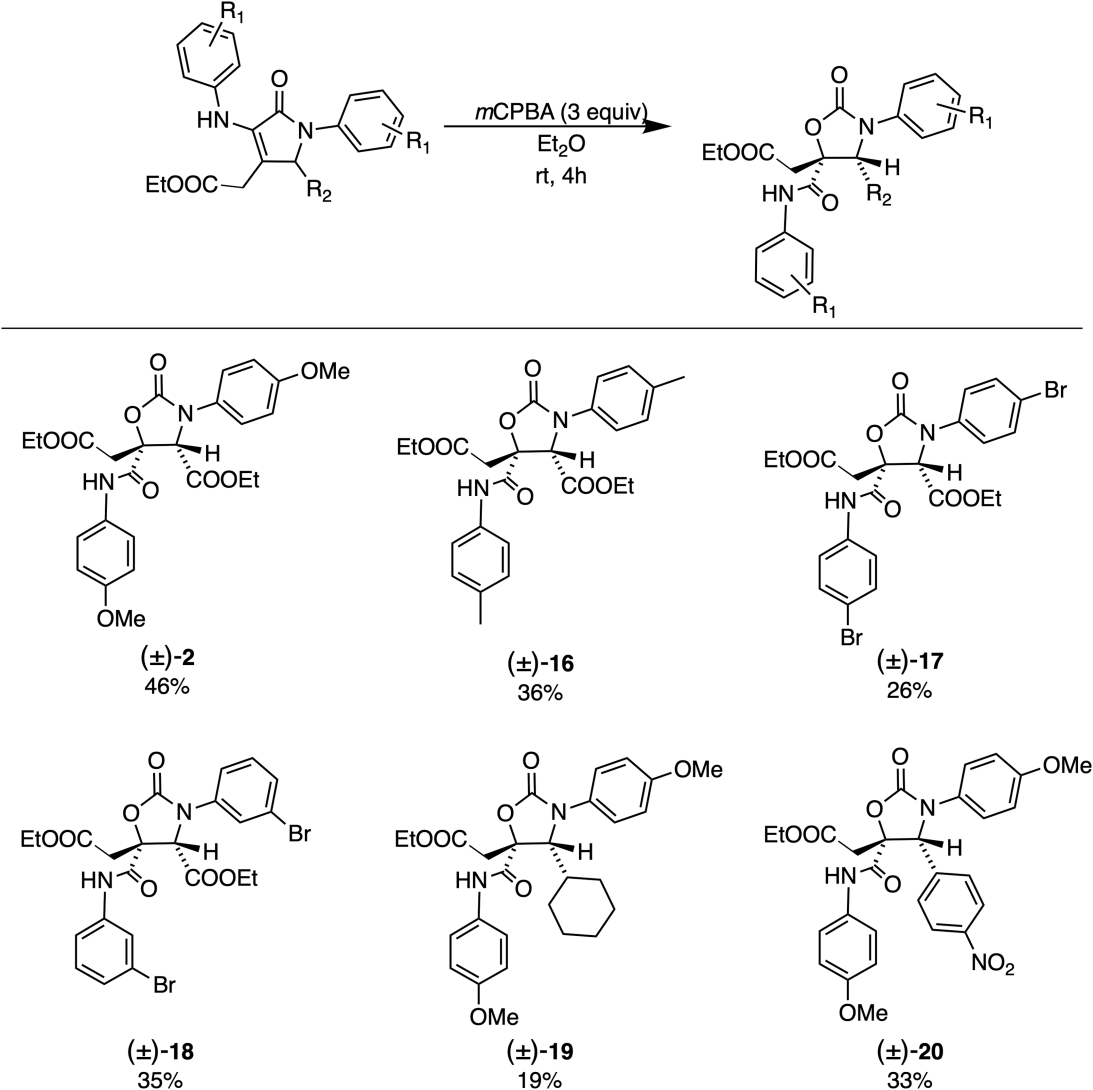
Substrate Scope^a^,^b^

a Reaction conditions: α,β-unsaturated γ-lactam (0.1 mmol, 1 equiv), *m*CPBA (0.3 mmol, 3 equiv), Et_2_O (4.0 mL).

b Isolated yield after column chromatography.

The transformation of the α,β-unsaturated γ-lactams into oxazolidinones is proposed to occur through a combination of a Baeyer–Villiger oxidation, an epoxidation, and a rearrangement (Scheme 2). The proposed reaction pathway was modeled by density functional theory (DFT) calculations using the M06-2X functional and the 6-31G** basis set, which found plausible transition states for each of the proposed reaction steps (Supporting Information).

**Scheme 2:**
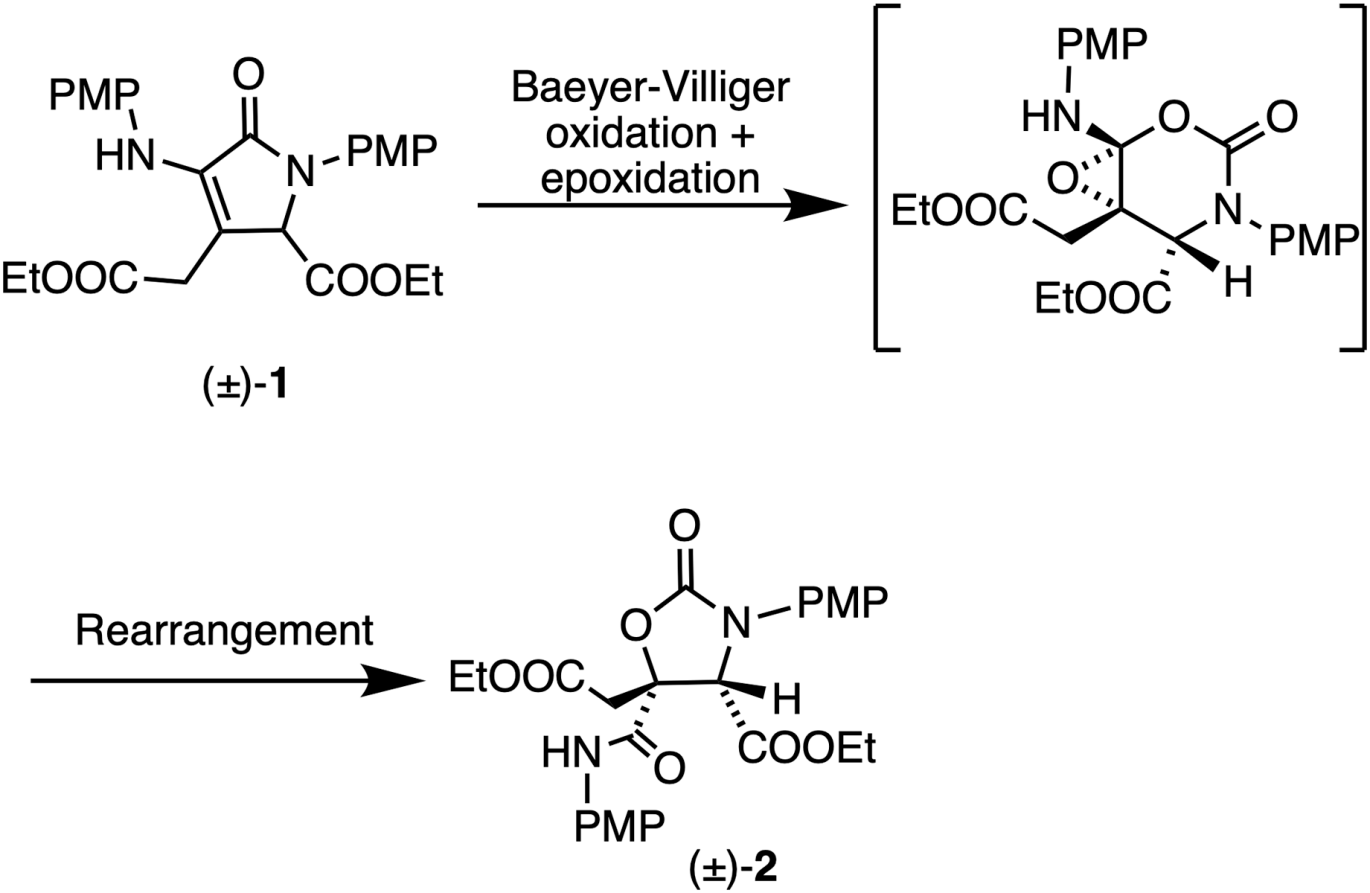
Proposed Reaction Pathway, PMP = *p*-Methoxyphenyl

Treatment of α,β-unsaturated ketones and aldehydes with *m*CPBA is known to yield a mixture of the corresponding epoxides, enol esters, and epoxy esters.^12^ In most reported cases, the Baeyer–Villiger oxidation appears to be faster than the epoxidation, although in the presence of an excess of *m*CPBA, both processes will occur and the doubly oxidized epoxy ester will be the major product.^12c^ As no intermediates were observed or isolated during the reaction of (±)-**1** with *m*CPBA, the order of the Baeyer–Villiger oxidation and the epoxidation could not be determined. Compound (±)-**1** is a highly functionalized molecule containing an α,β-unsaturated carbonyl, an amide, and an enamine. As such, the reactivity is difficult to predict.^13^ The proposed Baeyer–Villiger oxidation of compound (±)-**1** with *m*CPBA would constitute a rare example of Baeyer–Villiger oxidation of an unsaturated lactam. Typically, the treatment of unsaturated γ-lactams with peroxides or peracids yields the corresponding epoxide.^14^ In the case presented here, the presence of an amine substituent in the α-position of compound (±)-**1** (resulting in an enamine functionality) could enable the Baeyer–Villiger oxidation. The Baeyer–Villiger oxidation of α-imino ketones has been reported.^15^

The relative stereochemistry of product (±)-**2**, which was confirmed by XRD analysis and 2D NMR spectroscopy (Scheme 1 and Figure S26), indicates that of the two possible epoxide esters, only the diastereomer shown in Scheme 2 will rearrange into oxazolidinone product (±)-**2**. Given the modest yields of the reaction, it is reasonable to assume that both epoxides are formed, but only one of the diastereomers can rearrange into the desired oxazolidinone. Considering the calculated transition state for the rearrangement (Scheme 3), the most likely explanation for the selective rearrangement of only one diastereomer is steric hindrance. In the unreactive epoxide diastereomer, the R_2_ substituent is *cis* to the CH_2_COOEt and NH-PMP groups, which would result in a highly congested transition state for the rearrangement.

**Scheme 3:**
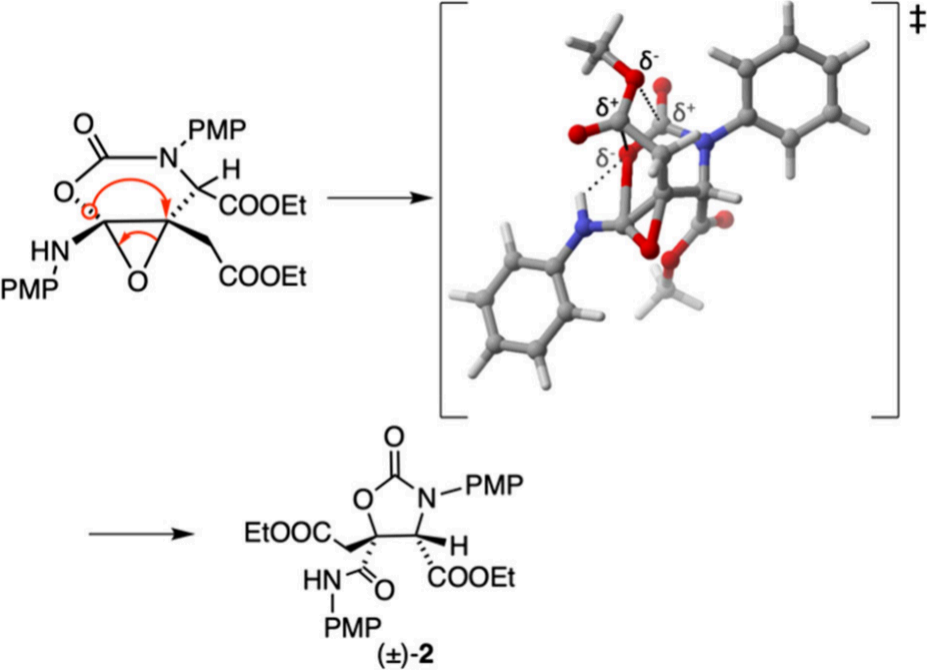
Mechanism of the Proposed Rearrangement Including the Calculated Transition State, PMP = *p*-Methoxyphenyl Dotted lines indicate stabilizing interactions.

The proposed rearrangement of the epoxy ester intermediate into oxazolidinone (±)-**2** is shown in Scheme 3 and consists of a 1,2-migration of the carbamate oxygen, resulting in the formation of a new amide. The observed relative stereochemistry of the product suggests that the rearrangement is concerted, as a stepwise rearrangement would lead to a mixture of two diastereomeric products. Rearrangements of epoxides through 1,2- alkyl^16^ or acyl^17^ migrations are well-known. Previously reported rearrangements are typically catalyzed by protic or Lewis acid and proceed in a stepwise manner.^16a,18^ In the calculated transition state for the rearrangement of the epoxy ester intermediate into oxazolidinone (±)-**2**, stabilizing electrostatic interactions can be observed between the migrating group and the ester group at position 4 (Scheme 3). The fact that no rearrangement product was observed when the CH_2_COOEt group was replaced by a hydrogen (Table S3) suggests that this stabilization is necessary for the rearrangement to proceed. The calculated transition state is also further stabilized by hydrogen bonding between the amine proton and the partially negative migrating oxygen.

In summary, a one-pot diastereospecific oxidative rearrangement of substituted α,β-unsaturated γ-lactams into 2-oxazolidinones was discovered. Reaction optimizations were performed, and a small substrate scope was established. The proposed reaction pathway includes a rare Baeyer–Villiger oxidation of an unsaturated lactam, an epoxidation, and an epoxide rearrangement through a 1,2-migration of a carbamate oxygen. DFT calculations indicate that the transition state of the rearrangement is stabilized by a CH_2_COOEt substituent at position 4. This was also observed experimentally as α,β-unsaturated γ-lactams without the ester substituent failed to give any product.

The discovered reaction adds α,β-unsaturated γ-lactams as starting materials to the synthetic tool box for oxazolidinones containing vicinal substituents. Although the presented reaction scope is somewhat limited by the availability of the starting α,β-unsaturated γ-lactams via the facile one-pot, three-component reaction, the diastereospecific nature of the transformation to vicinally substituted 2-oxazolidinones should trigger further exploration of this pathway to new antibiotics such as cytoxazone.

**Safety Statement.***m*CPBA was purified to 98% purity before use in the oxidative rearrangement; 95–100% *m*CPBA is explosive and can be detonated by shock or sparks. The purified *m*CPBA should therefore be stored in a refrigerator in a tightly closed container.

## Data Availability

The data underlying this study are available in the published article and its Supporting Information.

## Supporting Information Available

Optimization of the reaction conditions, experimental procedures and characterization data for all new compounds, copies of ^1^H and ^13^C NMR spectra, X-ray diffraction data for (±)-**2** and **4**, and procedures and energies for the DFT calculations (PDF)
DFT geometries (XYZ)

## Supplementary Material

jo4c02653_si_001.pdf

jo4c02653_si_002.xyz
