# Supplementary material for: Diastereospecific Synthesis of Vicinally Substituted 2-Oxazolidinones via Oxidative Rearrangement of α,β-Unsaturated γ-Lactams
Source: J Org Chem. 2025 Jan 6;90(2):1209–13. doi: 10.1021/acs.joc.4c02653 (PMC11744868; doi:10.1021/acs.joc.4c02653)
Supplement: Supplementary file 1 — jo4c02653_si_001.pdf [file jo4c02653_si_001.pdf]

Supporting information

Diastereospecific Synthesis of Vicinally Substituted 2-Oxazolidinones via Oxidative Rearrangement of α,β-Unsaturated γ-Lactams

Anna Lidskog, Yutang Li, Arvind Kumar Gupta, Abhishek Mishra, Anders Sundin, Kenneth Wärnmark*

Center for Analysis and Synthesis, Department of Chemistry, Lund University, Lund SE 221 00, Sweden

Email: kenneth.warnmark@chem.lu.se

Table of Content

Materials and methods *S3*

Optimization of oxidative rearrangement reaction *S4*

Experimental procedures and characterization data *S6*
General procedure for the synthesis of starting materials S6
Attempts to expand the substrate scope S12
General procedure for the oxidative rearrangement S12

Structure of side product 3 .... *S17*

Single crystal X-ray (scXRD) analysis of (±)-2 and 4 .... *S18*

DFT calculations *S20*

Copies of NMR spectra *S23*

References *S53*

Materials and methods

All moisture- or air-sensitive reactions were carried out under an atmosphere of dry nitrogen or argon using oven-dried glassware. All chemicals and solvents were used as received from commercial sources without further purification, with the exception of Et_2O which was obtained from a Braun SPS-800 system. Thin layer chromatography (TLC) was performed on Merck aluminum-backed silica gel 60 F-254 plates with visualization by ultraviolet light at 254 nm or by treatment with an aqueous solution of $KMnO_4$. Column chromatography was performed using silica gel (60 Å, 230-400 mesh, obtained from Sigma Aldrich). NMR spectra were recorded on 400 MHz (^{1}H at 400 MHz and ^{13}C at 101 MHz) Bruker Avance II or a 500 MHz (^{1}H at 500 MHz and ^{13}C at 126 MHz) Bruker Avance III HD spectrometer. Chemical shifts are referenced to the residual $CDCl_3$ peak as internal standard (^{1}H: 7.26 ppm, ^{13}C: 77.16 ppm). Chemical shifts (δ) are expressed in parts per million (ppm) and coupling constants (*J*) are reported in Hertz (Hz). The following abbreviations are used to indicate apparent multiplicities: s, singlet; d, doublet; dd, doublet of doublets; ddd, doublet of doublet of doublets; t, triplet; m, multiplet. Assignments of peaks were done using ^{1}H-^{1}H COSY, ^{1}H-^{1}H ROESY, ^{1}H-^{1}H NOESY and ^{1}H-^{13}C HMQC experiments. High resolution mass spectra (HRMS) were recorded on a Waters XEVO-G2 QTOF spectrometer using electron spray ionization in positive or negative mode. Elemental analyses were performed by A. Kolbe, Mikroanalytisches Laboratorium, Germany.

*m*CPBA was purchased from Acros (70-75%) and purified by a reported procedure[S1] to 98% purity. *Caution!* 95–100 % *m*CPBA is explosive and can be detonated by shock or sparks, whereas commercial 70–85 % *m*CPBA has been found to not be shock sensitive. The purified *m*CPBA should therefore be stored in a refrigerator in a tightly closed container.

Diethyl 2-oxopentanedionate was synthesized following a reported procedure[S2] and purified by vacuum distillation. Ethyl 3-(2-ethoxy-2-oxoethyl)-1-(4-methoxyphenyl)-4-((4-methoxyphenyl)amino)-5-oxo-2,5-dihydro-1*H*-pyrrole-2-carboxylate (**1**) was synthesized following a reported procedure.[S3]

Optimization of oxidative rearrangement reaction

Table S1. Optimization of oxidative rearrangement of (±)-**1**.[a]

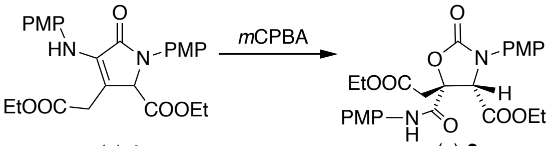

(±)-**1** (±)-**2**

	*m*CPBA (equiv.)	Reaction time (h)	Temp (°C)	[**1**] (mmol)	Additive	Solvent	Yield (%) [b]
1	3	24	rt	25	-	CH_2Cl_2	35
2	2	24	rt	25	-	CH_2Cl_2	10
3	4	24	rt	25	-	CH_2Cl_2	31
4	3	4	rt	25	-	CH_2Cl_2	33
5	3	0.5	rt	25	-	CH_2Cl_2	5
6	3	24	0	25	-	CH_2Cl_2	31
7	3	24	-40	25	-	CH_2Cl_2	32
8	3	24	-78	25	-	CH_2Cl_2	0 [c]
9	3	24	rt	50	-	CH_2Cl_2	trace
10	3	24	rt	12.5	-	CH_2Cl_2	33
11	3	24	rt	25	$NaHCO_3$	CH_2Cl_2	32
12	3	24	rt	25	Et_3N	CH_2Cl_2	0
13	3	24	rt	25	$NaHCO_3$ + 18-C-6	CH_2Cl_2	0
14	3	24	rt	25	-	Toluene	26
15	3	24	reflux	25	-	Toluene	33
16	3	24	rt	25	-	EtOH	25
17	3	24	reflux	25	-	EtOH	33
18	3	24	rt	25	-	MeCN	34
19	3	24	rt	25	-	$CHCl_3$	34
20	3	24	rt	25	-	THF	29
21	3	24	rt	25	-	Et_2O	44
22	3	4	rt	25	-	Et_2O	46
23	2.5	4	rt	25	-	Et_2O	40
24	3	4	rt	25	AcOH	Et_2O	35

[a] Reactions run with 0.1 mmol (±)-**1**. PMP = *p*-methoxyphenyl. [b] Isolated yields after column chromatography. [c] Insoluble reagents.

Table S2. Attempted oxidative rearrangement of (±)-**1** using other oxidants.

Oxidant	Equiv.	Reaction time (h)	Temp (°C)	Additive	Solvent	Yield **2** (%)
H_2O_2	3	2	rt	NaOH	MeOH	0 [b]
H_2O_2	6	2	rt	Et_3N	CH_2Cl_2	0 [b]
CF_3COOOH [a]	8	1.5	0	Na_2CO_3	CH_2Cl_2	0 [c]
t-BuOOH	5	40	rt	Triton B	THF	0 [c]

[a] Prepared in situ from H_2O_2 and $(CF_3CO)_2O$. [b] Starting material (±)-**1** tautomerized. [c] Starting material decomposed.

Table S3. Oxidative rearrangement of substrates (±)-**10-15**[a]

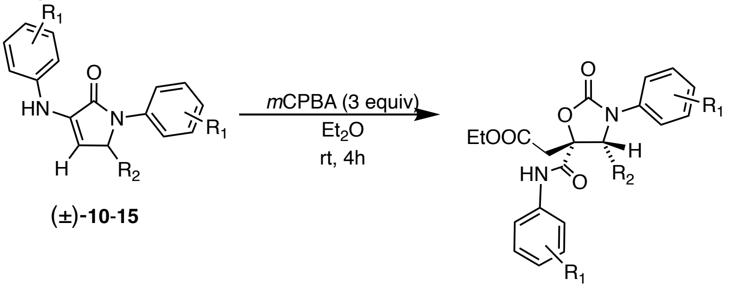

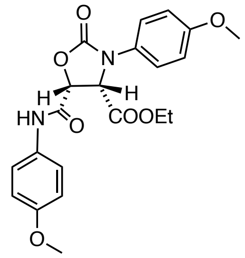	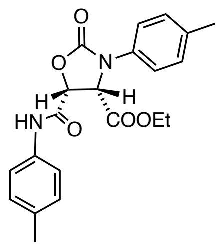	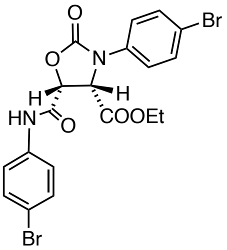
0%	0%	0%
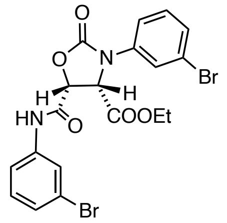	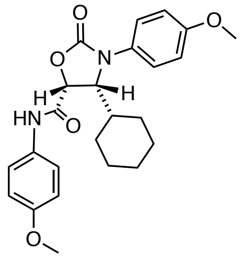	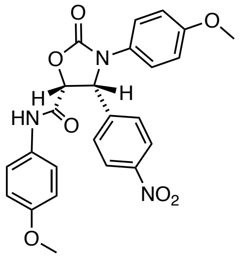
0%	0%	0%

[a] Reaction conditions: α,β-unsaturated γ-lactam (0.1 mmol, 1 eq), *m*CPBA (0.3 mmol, 3 eq), Et_2O (4.0 mL).

Experimental procedures and characterization data

General procedure for the synthesis of starting materials

Starting materials (±)-**5-15** were synthesized following a modified previously reported procedure.[S4]

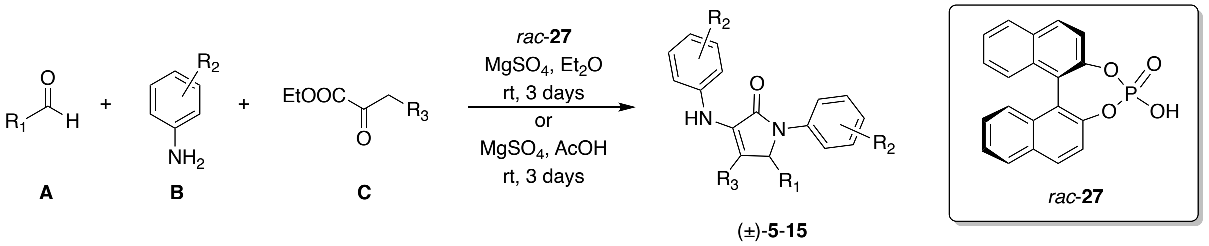

Procedure A:

A mixture of aldehyde **A** (2.5 mmol), amine **B** (5.0 mmol), ethyl pyruvate derivative **C** (7.5 mmol), phosphoric acid catalyst *rac*-**27** (10 µmol) and anhydrous $MgSO_4$ (3.0 g) in Et_2O (20 mL) was stirred at room temperature for 3 d. The reaction mixture was filtered and washed with CH_2Cl_2 (2 x 10 mL). The combined organic phases were concentrated under reduced pressure.

Procedure B:

A mixture of aldehyde **A** (2.5 mmol), amine **B** (5.0 mmol), ethyl pyruvate derivative **C** (7.5 mmol) and anhydrous $MgSO_4$ (3.0 g) in acetic acid (30 mL) was stirred at room temperature for 3 d. The reaction mixture was filtered and washed with CH_2Cl_2 (2 x 10 mL). The combined organic phases were concentrated under reduced pressure.

Compound (±)-5

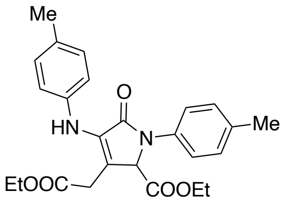

5

Compound (±)-**5** was synthesized following procedure A with ethyl glyoxalate (**A**), *p*-toluidine (**B**) and diethyl 2-oxopentanedioate (**C**). The crude was purified by silica gel chromatography (ø = 3 cm, h = 8 cm, *n*-heptane/EtOAc 8:1). Compound (±)-**5** was obtained as a pale yellow solid (288 mg, 26%).

^{1}H NMR (400 MHz, $CDCl_3$): δ 7.49 (d, *J* = 8.6 Hz, 2H), 7.18 (d, *J* = 8.4 Hz, 2H), 7.07 (d, *J* = 7.6 Hz, 2H), 6.91 (d, *J* = 8.3 Hz, 2H), 6.15 (s, 1H), 5.31 (s, 1H), 4.19–4.06 (m, 4H), 3.29 (d, *J* = 18 Hz, 1H), 3.07 (d, *J* = 18 Hz, 1H), 2.33 (s, 3H), 2.30 (s, 3H), 1.22 (t, *J* = 7.1 Hz, 3H), 1.14 (t, *J* = 7.1 Hz, 3H) ppm.

^{13}C{^{1}H} NMR (101 MHz, $CDCl_3$): δ 169.8, 168.6, 166.9, 137.9, 135.3, 134.9, 134.1, 133.1, 129.8 (2C), 129.7 (2C), 121.6 (2C), 120.4 (2C), 108.3, 64.6, 62.0, 61.2, 32.0, 21.0, 20.9, 14.2, 14.1 ppm.

HRMS (ESI+) m/z $[M+H]^+$ Calcd (%) for $C_{25}H_{29}N_2O_5$: 437.2086. Found: 437.2076.

IR (neat) ν_{max} 3309, 2981, 1732, 1690, 1613, 1513, 1385, 1292, 1252, 1178, 1022 cm^{-1}.

Anal. Calcd for $C_{25}H_{28}N_2O_5$: C 68.79, H 6.47, N 6.42; Found: C 68.64, H 6.41, N 6.36.

R_f = 0.2 (*n*-heptane/EtOAc 8:1)

Compound (±)-6

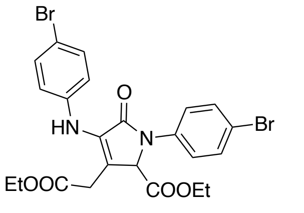

6

Compound (±)-**6** was synthesized following procedure A with ethyl glyoxalate (**A**), 4-bromoaniline (**B**) and diethyl 2-oxopentanedioate (**C**). The crude was purified by silica gel chromatography (ø = 3 cm, h = 10 cm, *n*-heptane/EtOAc 8:1). Compound (±)-**6** was obtained as a pale yellow solid (188 mg, 13%).

^{1}H NMR (400 MHz, $CDCl_3$) δ: 7.50 (d, J = 1.6 Hz, 4H), 7.36 (d, J = 8.8 Hz, 2H), 6.84 (d, J = 8.8 Hz, 2H), 6.21 (s, 1H), 5.34 (s, 1H), 4.22–4.08 (m, 4H), 3.31 (d, J = 18 Hz, 1H), 3.11 (d, J = 18 Hz, 1H), 1.24 (t, J = 7.2 Hz, 3H), 1.15 (t, J = 7.1 Hz, 3H) ppm.

^{13}C{^{1}H} NMR (101 MHz, $CDCl_3$) δ: 169.4, 168.0, 166.7, 139.9, 136.9, 133.5, 132.4 (2C), 132.3 (2C), 122.0 (2C), 121.5 (2C), 118.3, 115.7, 112.1, 64.2, 62.5, 61.5, 32.3, 14.3, 14.1 ppm.

HRMS (ESI-) m/z $[M-H]^-$ Calcd (%) for $C_{23}H_{21}N_2O_5Br_2$: 562.9817. Found: 562.9817.

IR (neat) ν_{max} 3309, 2981, 1733, 1693, 1589, 1489, 1380, 1287, 1253, 1181, 1021 cm^{-1}.

Anal. Calcd for $C_{23}H_{22}Br_2N_2O_5$: C 48.79, H 3.92, N 4.95; Found: C 48.61, H 3.95, N 4.91.

R*f* = 0.14 (*n*-heptane/EtOAc 6:1)

Compound (±)-7

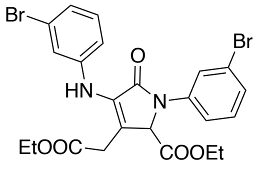

7

Compound (±)-**7** was synthesized following procedure B with ethyl glyoxalate (**A**), 3-bromoaniline (**B**) and diethyl 2-oxopentanedioate (**C**). The crude was purified by silica gel chromatography (ø = 3 cm, h = 12 cm, *n*-heptane/EtOAc 7:1), followed by precipitation with EtOH. Compound (±)-**7** was obtained as a white solid (152 mg, 11%).

^{1}H NMR (400 MHz, $CDCl_3$): δ 7.85 (t, J = 2.0 Hz, 1H), 7.55–7.52 (m, 1H), 7.32–7.30 (m, 1H), 7.27–7.23 (m, 1H), 7.15–7.10 (m, 3H), 6.90–6.87 (m, 1H), 6.18 (s, 1H), 5.37 (s, 1H), 4.20–4.14 (m, 4H), 3.36 (d, J = 18.0 Hz, 1H), 3.15 (d, J = 17.9 Hz, 1H), 1.26 (t, J = 7.2 Hz, 3H), 1.17 (t, J = 7.1 Hz, 3H) ppm.

^{13}C{^{1}H} NMR (101 MHz, $CDCl_3$) δ: 169.3, 167.8, 166.7, 142.3, 139.0, 133.1, 130.6, 130.6, 128.3, 126.0, 123.05, 123.04, 123.01, 122.7, 118.6, 118.4, 113.7, 64.3, 62.5, 61.7, 32.3, 14.3, 14.1 ppm.

HRMS (ESI+) m/z $[M+H]^+$ Calcd (%) for $C_{23}H_{23}Br_2N_2O_5$: 564.9966. Found: 564.9974.

IR (neat) ν_{max} 3313, 2981, 2927, 1735, 1707, 1591, 1511, 1480, 1374, 1249, 1187, 1023 cm^{-1}.

Anal. Calcd for $C_{23}H_{22}Br_2N_2O_5$: C 48.79, H 3.92, N 4.95; Found: C 48.61, H 4.01, N 4.85.

R*f* = 0.2 (*n*-heptane/EtOAc 8:1)

Compound (±)-8

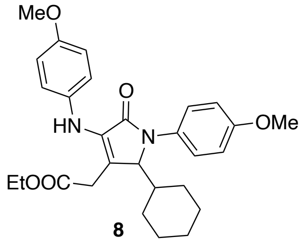

8

Compound (±)-**8** was synthesized following procedure A with cyclohexanecarboxaldehyde (**A**), *p*-anisidine (**B**) and diethyl 2-oxopentanedioate (**C**).The crude was purified by silica gel chromatography (ø = 3 cm, h = 8 cm, petroleum ether/EtOAc 3:1), followed by precipitation with EtOH. Compound (±)-**8** was obtained as a pale yellow solid (315 mg, 26%).

^{1}H NMR (400 MHz, $CDCl_3$): δ 7.31 (d, *J* = 8.8 Hz, 2H), 6.96–6.90 (m, 4H), 6.83 (d, *J* = 8.8 Hz, 2H), 5.87 (s, 1H), 4.72 (d, *J* = 2.0 Hz, 1H), 4.15–4.03 (m, 2H), 3.83 (s, 3H), 3.79 (s, 3H), 3.32 (d, *J* = 17.6 Hz, 1H), 3.03 (d, *J* = 17.6 Hz, 1H), 1.73–1.42 (m, 7H), 1.22 (t, *J* = 7.1 Hz, 3H), 1.16–0.94 (m, 3H), 0.81–0.70 (m, 1H) ppm.

^{13}C{^{1}H} NMR (101 MHz, $CDCl_3$): δ 170.2, 167.1, 157.7, 155.6, 134.9, 133.5, 130.6, 126.0 (2C), 122.5 (2C), 115.7, 114.50 (2C), 114.46 (2C), 66.2, 61.1, 55.7, 55.6, 40.2, 33.4, 29.0, 27.8, 26.9, 26.7, 26.5, 14.3 ppm.

HRMS (ESI+) m/z $[M+H]^+$ Calcd (%) for $C_{28}H_{35}N_2O_5$: 479.2546. Found: 479.2552.

IR (neat) ν_{max} 3297, 2929, 2853, 1732, 1677, 1509, 1443, 1403, 1242, 1157, 1033 cm^{-1}.

Anal. Calcd for $C_{28}H_{34}N_2O_5$: C 70.27, H 7.16, N 5.85; Found: C 70.26, H 7.15, N 5.84.

R*f* = 0.2 (PE/EtOAc 3:1)

Compound (±)-9

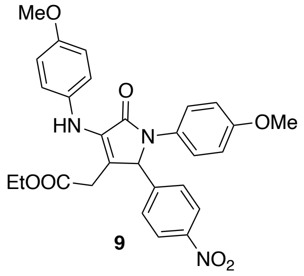

9

Compound (±)-**9** was synthesized following procedure A with 4-nitrobenzaldehyde (**A**), *p*-anisidine (**B**) and diethyl 2-oxopentanedioate (**C**).The crude was purified by silica gel chromatography (ø = 3 cm, h = 10 cm, CH_2Cl_2/EtOAc 30:1). Compound (±)-**9** was obtained as a yellow solid (813 mg, 63%).

^{1}H NMR (400 MHz, $CDCl_3$): δ 8.12 (d, *J* = 8.8 Hz, 2H), 7.38–7.32 (m, 4H), 6.99 (d, *J* = 8.4 Hz, 2H), 6.83–6.77 (m, 4H), 6.13 (s, 1H), 5.81 (s, 1H), 4.07–3.99 (m, 2H), 3.77 (s, 3H), 3.72 (s, 3H), 3.09 (d, *J* = 17.4 Hz, 1H), 2.46 (d, *J* = 17.4 Hz, 1H), 1.18 (t, *J* = 7.1 Hz, 3H) ppm.

^{13}C{^{1}H} NMR (101 MHz, $CDCl_3$): δ 170.1, 167.1, 157.2, 156.5, 148.0, 144.9, 133.5, 133.2, 129.9, 128.5 (2C), 124.3 (2C), 124.1 (2C), 123.6 (2C), 114.5 (2C), 114.4 (2C), 113.0, 65.4, 61.2, 55.6, 55.4, 31.5, 14.2 ppm.

HRMS (ESI+) m/z $[M+Na]^+$ Calcd (%) for $C_{28}H_{27}N_3O_7Na$: 540.1747. Found: 540.1736.

IR (neat) ν_{max} 3304, 2934, 1729, 1681, 1508, 1389, 1346, 1239, 1179, 1030 cm^{-1}.

Anal. Calcd for $C_{28}H_{27}N_3O_7$: C 64.98, H 5.26, N 8.12; Found: 64.91, 5.24, 8.07.

R*f* = 0.13 (petroleum ether/EtOAc 3:1)

Compound (±)-10

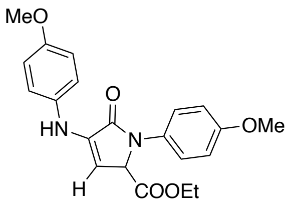

10

Compound (±)-**10** was synthesized following procedure B with ethyl glyoxalate (**A**), *p*-anisidine (**B**) and ethyl pyruvate (**C**).The crude was purified by silica gel chromatography (ø = 3 cm, h = 8 cm, CH_2Cl_2/EtOAc 40:1), followed by precipitation with EtOH. Compound (±)-**10** was obtained as a white solid (238 mg, 25%).

^{1}H NMR (500 MHz, $CDCl_3$): δ 7.48 (d, *J* = 7.2 Hz, 2H), 7.05 (d, *J* = 7.2 Hz, 2H), 6.92 (d, *J* = 7.2 Hz, 2H), 6.88 (d, *J* = 6.8 Hz, 2H), 6.57 (s, 1H), 5.83 (d, *J* = 2.8 Hz, 1H), 5.20 (d, *J* = 2.9 Hz, 1H), 4.19–4.06 (m, 2H), 3.80 (s, 3H), 3.79 (s, 3H), 1.16 (t, *J* = 7.1 Hz, 3H) ppm.

^{13}C{^{1}H} NMR (126 MHz, $CDCl_3$): δ 169.0, 166.8, 157.4, 154.9, 135.7, 134.4, 130.8, 123.1 (2C), 119.0 (2C), 114.8 (2C), 114.5 (2C), 97.2, 63.2, 62.0, 55.7, 55.5, 14.1 ppm.

HRMS (ESI+) m/z $[M+Na]^+$ Calcd (%) $C_{21}H_{22}N_2O_5Na$: 405.1426. Found: 405.1427.

IR (neat) ν_{max} 3302, 1742, 1687, 1651, 1547, 1510, 1406, 1247, 1179, 1114, 1029, 1016 cm^{-1}.

Anal. Calcd for $C_{21}H_{22}N_2O_5$: C 65.96, H 5.80, N 7.33; Found: C 65.81, H 5.73, N 7.29.

R$_f$ = 0.3 (CH_2Cl_2/EtOAc 40:1)

Compound (±)-11

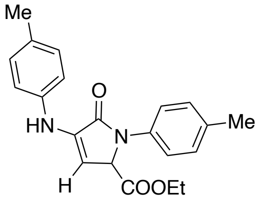

11

Compound (±)-**11** was synthesized following procedure B with ethyl glyoxalate (**A**), *p*-toluidine (**B**) and ethyl pyruvate (**C**). The crude was purified by silica gel chromatography (ø = 3 cm, h = 8 cm, *n*-heptane /EtOAc 9:1), followed by recrystallization from EtOH. Compound (±)-**11** was obtained as a white solid (112 mg, 13%).

^{1}H NMR (400 MHz, $CDCl_3$): δ 7.49 (d, *J* = 8.8 Hz, 2H), 7.20 (d, *J* = 8.0 Hz, 2H), 7.14 (d, *J* = 7.6 Hz, 2H), 7.00 (d, *J* = 8.4 Hz, 2H), 6.65 (s, 1H), 5.92 (d, *J* = 2.8 Hz, 1H), 5.26 (d, *J* = 2.9 Hz, 1H), 4.21–4.07 (m, 2H), 2.35 (s, 3H), 2.32 (s, 3H), 1.17 (t, *J* = 7.1 Hz, 3H) ppm.

^{13}C{^{1}H} NMR (101 MHz, $CDCl_3$): δ 168.9, 166.7, 138.4, 135.2, 135.10, 135.09, 131.2, 129.9 (2C), 129.8 (2C), 120.6 (2C), 117.2 (2C), 98.1, 62.8, 62.0, 20.9, 20.7, 14.0 ppm.

HRMS (ESI+) m/z $[M+H]^+$ Calcd (%) for $C_{21}H_{23}N_2O_3$: 351.1709. Found: 351.1710.

IR (neat) ν_{max} 3347, 2980, 1751, 1673, 1658, 1618, 1536, 1511, 1387, 1173, 1114, 1023 cm^{-1}.

Anal. Calcd for $C_{21}H_{22}N_2O_3$: C 71.98, H 6.33, N 7.99; Found: C 71.79, H 6.24, N 7.91.

R$_f$ = 0.5 (CH_2Cl_2/EtOAc 100:1)

Compound (±)-12

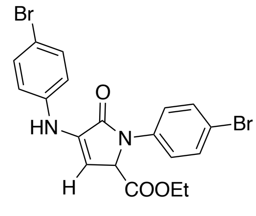

12

Compound (±)-**12** was synthesized following procedure B with ethyl glyoxalate (**A**), 4-bromoaniline (**B**) and ethyl pyruvate (**C**). After the reaction, the formed solid was collected by vacuum filtration and washed with Et_2O (3 x 20 mL), affording compound (±)-**12** as a white solid (341 mg, 28%).

^{1}H NMR (400 MHz, $CDCl_3$): δ 7.51 (s, 4H), 7.44 (d, *J* = 8.8 Hz, 2H), 6.96 (d, *J* = 9.2 Hz, 2H), 6.66 (s, 1H), 5.97 (d, *J* = 2.9 Hz, 1H), 5.26 (d, *J* = 2.8 Hz, 1H), 4.21–4.12 (m, 2H), 1.18 (t, *J* = 7.1 Hz, 3H) ppm.

^{13}C{^{1}H} NMR (126 MHz, $CDCl_3$): δ 168.2, 166.3, 139.8, 136.8, 134.5, 132.4 (2C), 132.3 (2C), 121.7 (2C), 118.7 (2C), 118.4, 114.2, 99.6, 62.5, 62.3, 14.0 ppm.

HRMS (ESI-) m/z $[M-H]^-$ Calcd (%) for $C_{19}H_{15}N_2O_3Br_2$: 476.9449. Found: 476.9449.

IR (neat) ν_{max} 3312, 1732, 1693, 1655, 1592, 1536, 1494, 1390, 1307, 1197, 1076, 1007 cm^{-1}.

Anal. Calcd for $C_{19}H_{16}Br_2N_2O_3$: C 47.53, H 3.36, N 5.83; Found: C 47.47, H 3.31, N 5.81.

R_f = 0.25 (*n*-heptane/EtOAc 6:1)

Compound (±)-13

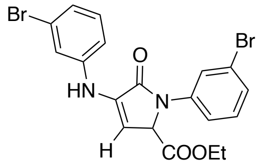

13

Compound (±)-**13** was synthesized following procedure B with ethyl glyoxalate (**A**), 3-bromoaniline (**B**) and ethyl pyruvate (**C**). The crude was purified by silica gel chromatography (ø = 3 cm, h = 8 cm, *n*-heptane/EtOAc 8:1), followed by recrystallization from EtOH. Compound (±)-**13** was obtained as an off-white solid (521 mg, 43%).

^{1}H NMR (400 MHz, $CDCl_3$): δ 7.85 (t, *J* = 2.0 Hz, 1H), 7.54–7.51 (m, 1H), 7.35–7.32 (m, 1H), 7.30–7.24 (m, 2H), 7.21-7.17 (m, 1H), 7.14–7.11 (m, 1H), 7.01–6.98 (m, 1H), 6.73 (s, 1H), 6.02 (d, *J* = 2.9 Hz, 1H), 5.27 (d, *J* = 2.9 Hz, 1H), 4.23–4.13 (m, 2H), 1.20 (t, *J* = 7.1 Hz, 3H) ppm.

^{13}C{^{1}H} NMR (101 MHz, $CDCl_3$): δ 168.2, 166.4, 142.1, 139.0, 134.3, 130.9, 130.6, 128.5, 125.0, 123.34, 123.25, 123.1, 119.8, 118.6, 115.9, 100.4, 62.6, 62.5, 14.1 ppm.

HRMS (ESI+) m/z $[M+H]^+$ Calcd (%) for $C_{19}H_{17}Br_2N_2O_3$: 478.9606. Found: 478.9613.

IR (neat) ν_{max} 3313, 1744, 1692, 1656, 1590, 1532, 1479, 1438, 1382, 1270, 1180, 1114, 1017 cm^{-1}.

Anal. Calcd for $C_{19}H_{16}Br_2N_2O_3$: C 47.53, H 3.36, N 5.83; Found: C 47.41, H 3.33, N 5.80.

R_f = 0.2 (*n*-heptane/EtOAc 8:1)

Compound (±)-14

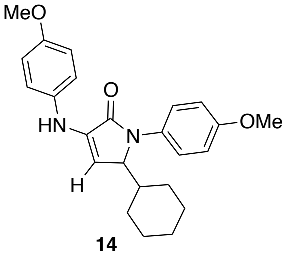

14

Compound (±)-**14** was synthesized following procedure B with cyclohexanecarboxaldehyde (**A**), *p*-anisidine (**B**) and ethyl pyruvate (**C**). The crude was purified by recrystallization from EtOH. Compound (±)-**14** was obtained as an off-white solid (388 mg, 39%).

^{1}H NMR (400 MHz, $CDCl_3$): δ 7.34 (d, *J* = 9.1 Hz, 2H), 7.05 (d, *J* = 9.2 Hz, 2H), 6.96 (d, *J* = 9.2 Hz, 2H), 6.90 (d, *J* = 8.8 Hz, 2H), 6.39 (br s, 1H), 5.87 (d, *J* = 2.5 Hz, 1H), 4.57 (t, *J* = 2.9, 2.9 Hz, 1H), 3.83 (s, 3H), 3.80 (s, 3H), 1.76–1.59 (m, 5H), 1.34–1.18 (m, 3H), 1.11–0.79 (m, 3H) ppm.

^{13}C{^{1}H} NMR (101 MHz, $CDCl_3$): δ 166.7, 157.5, 154.4, 135.4, 134.4, 130.0, 125.2 (2C), 118.5 (2C), 114.9 (2C), 114.6 (2C), 102.1, 65.1, 55.8, 55.6, 38.8, 30.4, 26.7, 26.6, 25.7, 25.3 ppm.

HRMS (ESI+) m/z $[M+H]^+$ Calcd (%) for $C_{24}H_{29}N_2O_3$: 393.2178. Found: 393.2186.

IR (neat) ν_{max} 3308, 2928, 1672, 1646, 1545, 1512, 1430, 1412, 1242, 1180, 1114, 1032 cm^{-1}.

Anal. Calcd for $C_{24}H_{28}N_2O_3 \cdot 0.2H_2O$: C 72.78, H 7.23, N 7.07; Found: C 72.61, H 7.02, N 7.04

R*f* = 0.4 (petroleum ether/EtOAc 3:1)

Compound (±)-15

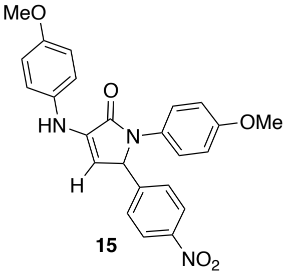

15

Compound (±)-**15** was synthesized following procedure B with 4-nitrobenzaldehyde (**A**), *p*-anisidine (**B**) and ethyl pyruvate (**C**). The crude was purified by recrystallization from EtOH. Compound (±)-**15** was obtained as an off-white solid (231 mg, 24%).

^{1}H NMR (500 MHz, $CDCl_3$): δ 8.12 (d, *J* = 8.5 Hz, 2H), 7.36 (d, *J* = 9.0 Hz, 2H), 7.33 (d, *J* = 9.0 Hz, 2H), 7.03 (d, *J* = 9.0 Hz, 2H), 6.86 (d, *J* = 9 Hz, 2H), 6.82 (d, *J* = 9.0 Hz, 2H), 6.53 (s, 1H), 5.88 (d, *J* = 2.5 Hz, 1H), 5.68 (d, *J* = 2.5 Hz, 1H), 3.78 (s, 3H), 3.74 (s, 3H) ppm.

^{13}C{^{1}H} NMR (126 MHz, $CDCl_3$): δ 166.8, 157.2, 154.7, 147.6, 145.5, 134.3, 133.8, 129.6, 127.7 (2C), 124.2 (2C), 123.6 (2C), 118.7 (2C), 114.7 (2C), 114.4 (2C), 104.1, 63.8, 55.5, 55.3 ppm.

HRMS (ESI+) m/z $[M+H]^+$ Calcd (%) for $C_{24}H_{22}N_3O_5$: 432.1559. Found: 432.1560.

IR (neat) ν_{max} 3311, 1677, 1648, 1512, 1393, 1348, 1300, 1248, 1175, 1111, 1035 cm^{-1}.

Anal. Calcd $C_{24}H_{21}N_3O_5 \cdot 0.2H_2O$: C 66.26, H 4.96, N 9.66; Found: C 66.12, H 4.93, N 9.71

R*f* = 0.2 (petroleum ether/EtOAc 3:1)

Attempts to expand the substrate scope

Attempts were made to expand the substrate scope to include α,β-unsaturated γ-lactams with different substituents in the 4-position ($R_3 = CH_2COCH_3$, CH_2CN or Ph, see structure below). In two cases (R_3 = CH_2CN and Ph), syntheses of the corresponding ethyl pyruvate derivates needed for the three-component reaction (ethyl 4-cyano-2-oxo-butanoate and ethyl 2-oxo-3-phenylpropanoate) were unsuccessful. For $R_3 = CH_2COCH_3$, although the ethyl pyruvate derivative was successfully synthesized, repeated attempts at accessing the substrate through the one-pot three-component reaction (procedure A or B described above) using ethyl glyoxalate (**A**), *p*-toluidine (**B**) and ethyl 2,5-dioxohexanoate (**C**) were unsuccessful.

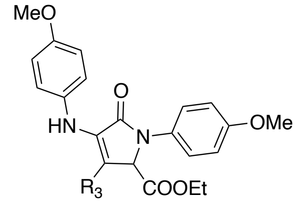

$R_3 = CH_2COCH_3$, CH_2CN, Ph

General procedure for the oxidative rearrangement

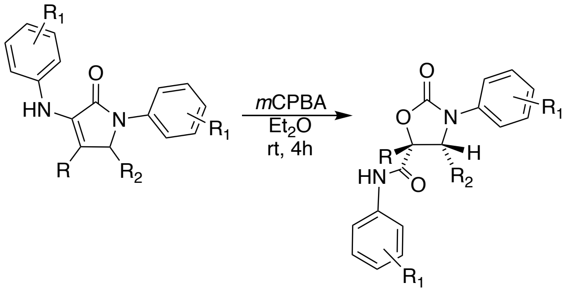

A solution of α,β-unsaturated γ-lactam (0.1 mmol) and *m*CPBA (0.3 mmol) in dry Et_2O (4.0 mL) was stirred at room temperature for 4 h. The crude was purified by silica gel chromatography.

Compound (±)-2

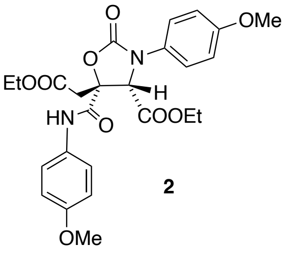

2

The general procedure was followed with (±)-**1**. The crude was purified by silica gel chromatography (ø = 2 cm, h = 8 cm, Et_2O/petroleum ether 2:1). Compound (±)-**2** was obtained as a white solid (23.2 mg, 46%).

^{1}H NMR (400 MHz, $CDCl_3$): δ 8.35 (s, 1H), 7.45 (d, *J* = 9.0 Hz, 2H), 7.37 (d, *J* = 9.1 Hz, 2H), 6.90 (d, *J* = 9.2 Hz, 2H), 6.87 (d, J = Hz, 2H), 5.01 (s, 1H), 4.20–4.14 (m, 2H), 4.11–4.04 (m, 2H), 3.79 (s, 6H), 3.29 (d, *J* = 16.0 Hz, 1H), 3.21 (d, *J* = 16.1 Hz, 1H), 1.22 (t, *J* = 7.1 Hz, 3H), 1.08 (t, *J* = 7.1 Hz, 3H) ppm.

^{13}C{^{1}H} NMR (101 MHz, $CDCl_3$): δ 167.8, 167.7, 165.3, 158.3, 157.1, 153.5, 129.4, 128.6, 124.4 (2C), 122.0 (2C), 114.7 (2C), 114.3 (2C), 80.0, 67.1, 62.6, 61.6, 55.49, 55.47, 42.3, 14.0, 13.8 ppm.

HRMS (ESI+) m/z $[M+H]^+$ Calcd (%) for $C_{25}H_{29}N_2O_9$: 501.1873. Found: 501.1868.

IR (neat) ν_{max} 2987, 1774, 1744, 1685, 1514, 1386, 1250, 1200, 1154, 1030 cm^{-1}.

Anal. Calcd for $C_{25}H_{28}N_2O_9$•$0.2H_2O$: C 59.57, H 5.68, N 5.56; Found: C 59.32, H 5.71, N 5.53

R$_f$ = 0.2 (Et_2O/petroleum ether 2:1)

*Side product **3***

Compound **3** was obtained as a yellow glass (7.6 mg, 15%).

^{1}H NMR (400 MHz, $CDCl_3$): δ 7.51–7.47 (m, 2H), 7.39–7.34 (m, 2H), 6.96–6.92 (m, 2H), 6.88–6.84 (m, 2H), 6.15 (br s, 1H), 5.05 (s, 1H), 4.28 (q, *J* = 7.2 Hz, 2H), 4.12 (q, *J* = 7.2 Hz, 2H), 3.84 (s, 3H), 3.78 (s, 3H), 3.34 (d, *J* = 14 Hz, 1H), 3.11 (d, *J* = 14 Hz, 1H), 1.30 (t, *J* = 7.2 Hz, 3H), 1.21 (t, *J* = 7.2 Hz, 3H) ppm.

^{13}C{^{1}H} NMR (101 MHz, $CDCl_3$): δ 169.0, 168.3, 161.7, 158.2, 157.3, 142.4, 137.7, 130.3, 125.7 (2C), 123.6 (2C), 114.5 (2C), 113.8 (2C), 74.2, 67.3, 62.4, 61.7, 55.7, 55.6, 41.9, 14.2, 14.1 ppm.

HRMS (ESI+) m/z $[M+H]^+$ Calcd (%) for $C_{25}H_{29}N_2O_9$: 501.1873. Found: 501.1876.

R$_f$ = 0.15 (Et_2O/PE, 2:1, v/v)

*Side product **4** (hydrolysis product)*

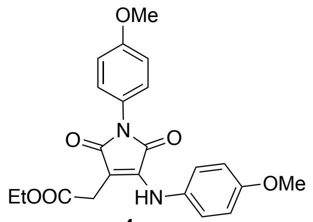

4

Compound **4** could be isolated from the reaction crude if base was added, either during workup (wash with $NaHCO_3$) or purification (Et_3N added to column for easier removal of *m*-chlorobenzoic acid). Compound **4** was obtained as a yellow glass in yields ranging from 10% (4.1 mg) to 35% (14.2 mg) depending on the amount of base added.

^{1}H NMR (400 MHz, $CDCl_3$): δ 7.30 (d, *J* = 8.8 Hz, 2H), 7.13 (d, *J* = 8.4 Hz, 2H), 6.96 (d, *J* = 8.8 Hz, 3H), 6.89 (d, *J* = 8.8 Hz, 2H), 3.95 (q, *J* = 7.1 Hz, 2H), 3.82 (s, 6H), 3.11 (s, 2H), 1.14 (t, *J* = 7.1 Hz, 3H) ppm.

^{13}C{^{1}H} NMR (101 MHz, $CDCl_3$): δ 172.3, 170.20, 167.4, 158.9, 158.5, 141.6, 129.9, 127.3 (2C), 126.5 (2C), 124.7, 114.6 (2C), 114.5 (2C), 93.6, 61.0, 55.7, 55.6, 28.2, 14.2 ppm.

HRMS (ESI+) m/z $[M+H]^+$ Calcd (%) for $C_{22}H_{23}N_2O_6$: 411.1556. Found: 411.1562.

R$_f$ = 0.18 (Et_2O/PE, 2:1, v/v)

Compound (±)-16

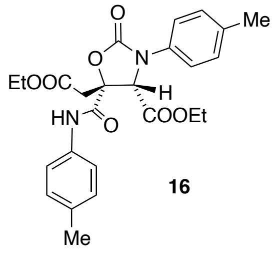

16

The general procedure was followed with (±)-**5**. The crude product was purified by silica gel chromatography (ø = 2 cm, h = 8 cm, *n*-heptane/EtOAc 8:1). Compound (±)-**16** was obtained as a light yellow solid (17 mg, 36%).

^{1}H NMR (400 MHz, CDCl$_3$): δ 8.31 (s, 1H), 7.42 (d, *J* = 8.5 Hz, 2H), 7.35 (d, *J* = 8.5 Hz, 2H), 7.18 (d, *J* = 8.0 Hz, 2H), 7.14 (d, *J* = 8.0 Hz, 2H), 5.05 (s, 1H), 4.18–4.13 (m, 2H), 4.10–4.04 (m, 2H), 3.30 (d, *J* = 16.0 Hz, 1H), 3.20 (d, *J* = 16.0 Hz, 1H), 2.33 (s, 3H), 2.32 (s, 3H), 1.20 (t, *J* = 7.2 Hz, 3H), 1.08 (t, *J* = 7.1 Hz, 3H) ppm.

^{13}C{^{1}H} NMR (101 MHz, CDCl$_3$): δ 167.7, 167.6, 165.3, 152.9, 136.4, 135.1, 133.8, 133.4, 130.0 (2C), 129.6 (2C), 121.5 (2C), 120.2 (2C), 80.1, 66.5, 62.7, 61.6, 42.4, 20.93, 20.92, 14.0, 13.8 ppm.

HRMS (ESI+) m/z [M+H]$^+$ Calcd (%) for $C_{25}H_{29}N_2O_7$: 469.1975. Found: 469.1971.

IR (neat) ν_{max} 3272, 2937, 1775, 1730, 1660, 1514, 1376, 1198, 1138, 1095, 1063, 1016 cm^{-1}.

Anal. Calcd for $C_{25}H_{28}N_2O_7$: C 64.09, H 6.02, N 5.98; Found: C 64.05, H 6.03, N 5.96

R$_f$ = 0.1 (*n*-heptane/EtOAc 8:1)

Compound (±)-17

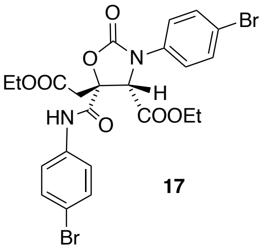

17

The general procedure was followed with (±)-**6**. The crude product was purified by silica gel chromatography (ø = 2 cm, h = 8 cm, CH_2Cl_2/MeOH 1000:3). Compound (±)-**17** was obtained as a light yellow solid (16 mg, 26%).

^{1}H NMR (400 MHz, CDCl$_3$): δ 8.39 (s, 1H), 7.53–7.49 (m, 2H), 7.45 (d, *J* = 1.5 Hz, 4H), 7.40–7.37 (m, 2H), 5.05 (s, 1H), 4.15 (q, *J* = 7.2 Hz, 2H), 4.08 (q, *J* = 7.2 Hz, 2H), 3.30 (d, *J* = 16.2 Hz, 1H), 3.20 (d, *J* = 16.3 Hz, 1H), 1.21 (t, *J* = 7.2 Hz, 3H), 1.10 (t, *J* = 7.1 Hz, 3H) ppm.

^{13}C{^{1}H} NMR (101 MHz, CDCl$_3$): δ 167.6, 167.3, 165.5, 152.4, 135.3, 135.1, 132.6 (2C), 132.2 (2C), 122.5 (2C), 121.8 (2C), 119.6, 118.3, 80.1, 66.0, 63.0, 61.8, 42.3, 14.0, 13.8 ppm.

HRMS (ESI+) m/z [M+Na]$^+$ Calcd (%) for $C_{23}H_{22}Br_2N_2O_7Na$: 618.9691. Found: 618.9685.

IR (neat) ν_{max} 3336, 2923, 2853, 1775, 1741, 1689, 1592, 1532, 1491, 1377, 1198, 1155, 1073, 1022 cm^{-1}.

Anal. Calcd for $C_{23}H_{22}Br_2N_2O_7 \bullet 0.2C_6H_{14}$: C 47.23, H 4.06, N 4.55; Found: C 47.26, H 3.89, N 4.50

R$_f$ = 0.2 (petroleum ether/diethyl ether 2:1)

Compound (±)-18

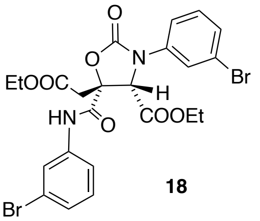

18

The general procedure was followed with (±)-**7**. The crude product was purified by silica gel chromatography (ø = 2 cm, h = 8 cm, CH_2Cl_2/EtOAc 200:1). Compound (±)-**18** was obtained as a light-yellow solid (18 mg, 35%).

^{1}H NMR (400 MHz, CDCl$_3$): δ 8.36 (s, 1H), 7.85 (t, *J* = 2.0 Hz, 1H), 7.73 (t, *J* = 2.0 Hz, 1H), 7.43–7.40 (m, 2H), 7.38–7.30 (m, 2H), 7.26 (t, *J* = 8.0, 1H), 7.21 (t, *J* = 8.0 Hz, 1H), 5.05 (s, 1H), 4.19 – 4.09

(m, 4H), 3.31 (d, J = 16.8 Hz, 1H), 3.19 (d, J = 16.4 Hz, 1H), 1.22 (t, J = 7.2 Hz, 3H), 1.14 (t, J = 7.1 Hz, 3H) ppm.

^{13}C{^{1}H} NMR (101 MHz, $CDCl_3$): δ 167.5, 167.3, 165.5, 152.2, 137.4, 137.3, 130.7, 130.4, 129.3, 128.6, 123.8, 123.2, 123.1, 122.8, 119.1, 118.7, 80.1, 65.9, 63.1, 61.8, 42.3, 14.0, 13.8 ppm.

HRMS (ESI+) m/z $[M+Na]^+$ Calcd (%) for $C_{23}H_{22}Br_2N_2O_7Na$: 618.9691. Found: 618.9678.

IR (neat) ν_{max} 3369, 2927, 2856, 1779, 1737, 1717, 1659, 1593, 1480, 1381, 1350, 1200, 1106 cm^{-1}.

Anal. Calcd for $C_{23}H_{22}Br_2N_2O_7$: C 46.18, H 3.71, N 4.68; Found: C 46.06, H 3.76, N 4.64

R$_f$ = 0.2 (CH_2Cl_2/EtOAc 200:1)

Compound (±)-19

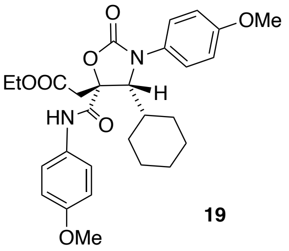

19

The general procedure was followed with (±)-**8**. The crude product was purified by silica gel chromatography (ø = 2 cm, h = 8 cm, $CHCl_3$/MeOH 120:1). Compound (±)-**19** was obtained as a yellow solid (10 mg, 19%).

^{1}H NMR (400 MHz, $CDCl_3$): δ 8.32 (s, 1H), 7.44 (d, J = 8.8 Hz, 2H), 7.38 (d, J = 9.2 Hz, 2H), 6.95–6.89 (m, 4H), 4.35 (d, J = 1.3 Hz, 1H), 4.17 – 4.04 (m, 2H), 3.83 (s, 3H), 3.81 (s, 3H), 3.28 (d, J = 15.2 Hz, 1H), 3.09 (d, J = 15.2 Hz, 1H), 2.02–1.96 (m, 1H), 1.80–1.77 (m, 1H), 1.66–1.59 (m, 2H), 1.52–1.49 (m, 2H), 1.39–1.34 (m, 1H), 1.19 (t, J = 7.2 Hz, 3H), 1.13–1.03 (m, 2H), 0.89–0.80 (m, 1H), 0.68–0.59 (m, 1H) ppm.

^{13}C{^{1}H} NMR (101 MHz, $CDCl_3$): δ 168.1, 165.0, 157.9, 157.3, 153.4, 130.3, 129.3, 125.3 (2C), 122.4 (2C), 114.44 (2C), 114.42 (2C), 82.3, 69.2, 61.5, 55.51, 55.48, 43.5, 40.4, 32.4, 27.2, 26.8, 25.9, 25.9, 14.14 ppm.

HRMS (ESI+) m/z $[M+H]^+$ Calcd (%) for $C_{28}H_{35}N_2O_7$: 511.2444. Found: 511.2449.

IR (neat) ν_{max} 3313, 2930, 2853, 1739, 1679, 1648, 1509, 1443, 1402, 1230, 1245, 1174, 1031 cm^{-1}.

Anal. Calcd for $C_{28}H_{34}N_2O_7$: C 65.87, H 6.71, N 5.49; Found: C 65.61, H 6.89, N 5.43

R$_f$ = 0.2 ($CHCl_3$/MeOH 120:1)

Compound (±)-20

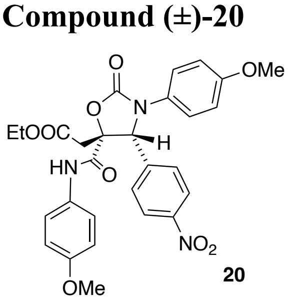

20

The general procedure was followed with (±)-**9**. The crude product was purified by silica gel chromatography (ø = 2 cm, h = 8 cm, *n*-heptane/EtOAc 3:2). Compound (±)-**20** was obtained as a yellow solid (35 mg, 33%).

^{1}H NMR (400 MHz, $CDCl_3$): δ 8.78 (s, 1H), 8.12 (d, J = 8.8 Hz, 2H), 7.35 (d, J = 9.0 Hz, 2H), 7.29 (d, J = 8.8 Hz, 2H), 6.96 (d, J = 8.4 Hz, 2H), 6.79 (d, J = 9.0 Hz, 2H), 6.68 (d, J = 9.2 Hz, 2H), 6.07 (s,

1H), 4.16 (q, J = 7.1 Hz, 2H), 3.75 (s, 3H), 3.72 (s, 3H), 3.60 (d, J = 1.8 Hz, 2H), 1.24 (t, J = 7.2 Hz, 3H) ppm.

^{13}C{^{1}H} NMR (101 MHz, $CDCl_3$): δ 196.1, 166.6, 163.3, 159.5, 157.1, 156.6, 148.3, 138.5, 132.1 (2C), 131.7, 129.8 (2C), 129.7, 123.8 (2C), 121.6 (2C), 114.3 (2C), 114.2 (2C), 71.9, 62.0, 55.6, 55.4, 47.3, 14.2 ppm.

HRMS (ESI+) m/z $[M+Na]^+$ Calcd (%) for $C_{28}H_{27}N_3O_9Na$: 572.1645. Found: 572.1659.

IR (neat) ν_{max} 3310, 2928, 1723, 1686, 1650, 1606, 1510, 1347, 1299, 1247, 1173, 1030 cm^{-1}.

Anal. Calcd for $C_{28}H_{27}N_3O_9$•$0.2H_2O$: C 60.80, H 4.99, N 7.60; Found: 60.59, H 4.99, N 7.54

R*f* = 0.34 (n-heptane/EtOAc 1:1)

Structure of side product (±)-3

Side product (±)-**3** could be isolated in ~15% yield from the reaction of (±)-**1** and *m*CPBA. Similar side products were also observed in the reactions with the other substrates. According to HRMS, compound **3** has the same molecular weight as product (±)-**2**. In addition, the ^{1}H and ^{13}C NMR spectra of **3** (Figure S48-49) are largely similar to the spectra for product (±)-**2** (Figure S27-28). Attempted crystallizations failed to yield crystals suitable for X-ray diffraction studies, making the identification of the exact structure of compound **3** difficult. A suggested structure and discussion of the support for said structure is presented below.

Comparing the ^{1}H NMR spectra of product (±)-**2** and side product (±)-**3**, the most significant difference is that the amide proton resonance in the spectrum of (±)-**2** at 8.34 ppm (Figure S27) is replaced with a broad peak at 6.15 ppm in the spectrum of (±)-**3** (Figure S48). The chemical shift (6.15 ppm) suggests that this resonance corresponds to either an amide proton or a particularly deshielded hydroxyl proton. Looking at the ^{13}C NMR spectra, there are differences in the chemical shifts of some of the carbonyl peaks, as well as some of the other peaks (Figure S28 vs Figure S49, Table S4). Taken together, this indicates a structure with a different connectivity than the cyclic carbamate of product (±)-**2**. Our suggested structure is the dihydrouracil shown in Figure S1. This structure could form if the oxidation (Baeyer-Villiger and epoxidation) is followed by ring-opening and subsequent nucleophilic attack by one of the nitrogens. The OH group in the suggested structure of (±)-**3** is positioned on to the β-carbon of two esters. The observed downfield chemical shift of the hydroxyl proton (6.15 ppm) could be explained by intramolecular hydrogen bonding between the hydroxyl and the C15 carbonyl oxygen. The suggested relative stereochemistry is supported by a cross-peak between H2 and H4 in the NOESY spectrum (Figure S51). In terms of carbonyl carbons, compound (±)-**2** contains two exocyclic esters, one exocyclic amide, and one cyclic carbamate. The suggested structure of side product (±)-**3** also has two exocyclic esters, but the other two carbonyls are an endocyclic amide and a cyclic urea group (the dihydrouracil). Comparing the ^{13}C NMR spectra of product (±)-**2** and side product (±)-**3**, the biggest difference is seen for one of the carbonyl carbons, and the *ipso*-carbon in one of the methoxy phenyl groups (Table S4). This is in line with what could be expected for the suggested structure of (±)-**3**, in which the exocyclic amide of (±)-**2** is replaced by an endocyclic amide (C8 in Figure S1). The change in chemical shift for C1 (from 80.0 ppm in (±)-**2** to 74.2 ppm in (±)-**3**, Table S4) could also support the suggested structure of side product (±)-**3**, as a quaternary carbon bound to a hydroxyl group and an endocyclic amide (as is found in (±)-**3**) is expected to give a different chemical shift than a quaternary carbon bound to an exocyclic amide and an endocyclic carbamate (as is found in (±)-**2**).

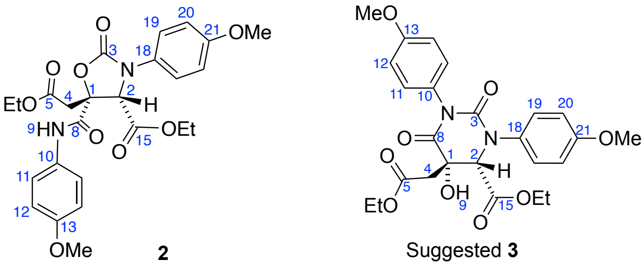

2 Suggested **3**

Figure S1. Structure of product (±)-**2** and suggested structure of side product (±)-**3**.

Table S4. Comparison of selected ^{13}C NMR shifts (ppm) of **2** and **3**.

		(±)-**2**	(±)-**3**[a]	Δδ
Carbonyls	C3	153.5	157.3	3.8
	C5	167.7 or 167.8	169.0	1.3
	C8	167.7 or 167.8	142.4	-25.4
	C15	165.3	168.3	3.0
Other	C1	80.0	74.2	-5.8
	C10	129.4	137.7	8.3

[a] Assignment based on suggested structure shown in Figure S1.

Single crystal X-ray (scXRD) analysis of (±)-2 and 4

All SC-XRD measurements were performed using graphite-monochromatized Mo Kα radiation (λ = 0.71073 Å) using the Agilent Xcalibur Sapphire3 diffractometer high-brilliance IμS radiation source. Data collections were performed at 295 K and 150K. The structure was solved by direct methods and refined by full-matrix least-squares techniques against F2 using all data (SHELXT, SHELXS).[S5-6] All non-hydrogen atoms were refined with anisotropic displacement parameters if not stated otherwise. Absorption was corrected using multi-scan empirical absorption correction with spherical harmonics as implemented in the SCALE3 ABSPACK scaling algorithm.[S7] Hydrogen atoms were constrained in geometric positions to their parent atoms using OLEX2 software.[S8]

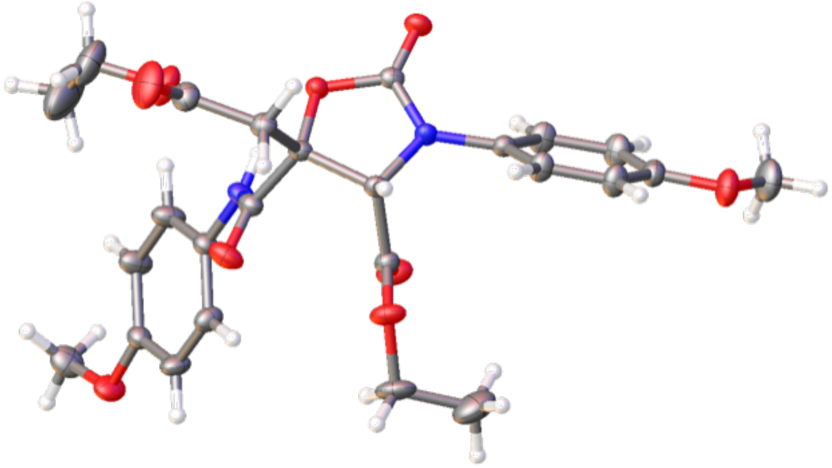

Figure S2. Crystal structure of (±)-**2**. Thermal ellipsoids are shown at 30% probability.

Table S5. Crystal data for (±)-**2**. *Crystallization*: A solution (±)-**2** in $CHCl_3$ was covered by a layer of EtOH. After three days, single crystals suitable for X-ray analysis were obtained.

Chemical formula	$C_{25}H_{28}N_2O_9$	
Formula weight	500.49	
Collection temperature /K	293(2)	
Crystal size /mm^3	0.35 x 0.26 x 0.18	
Wavelength /Å	0.71073	
Crystal system	Triclinic	
Space group	$P\bar{1}$	
Unit cell dimensions:	a = 6.0112(8) Å	α = 88.841(6) °
	b = 12.8634(11) Å	β = 88.088(7) °
	c = 15.9914(10) Å	γ = 87.697(9) °
Unit cell volume /Å^3	1234.6(2) Å^3	
Z, Calculated density /Mg/m^3	2, 1.346	
Radiation type	MoKα	
Absorption coefficient, m/mm^{-1}	0.103	
No. reflections collected / unique	11156 / 5712	
R_{int}	0.0343	
Completeness to theta = 25.000 /%	99.8	
Data / restraints / parameters	5712 / 15 / 329	
Goodness of fit on F^2	1.022	
Final R indices $(I > 2\sigma(I))$	R_1 = 0.0705, wR_2 = 0.1398	
R indices *(all data)*	R_1 = 0.1286, wR_2 = 0.1694	
Absolute structure parameter	–	
Largest diff. peak and hole /e-/Å^3	0.469 and -0.287	
CCDC	2352162	

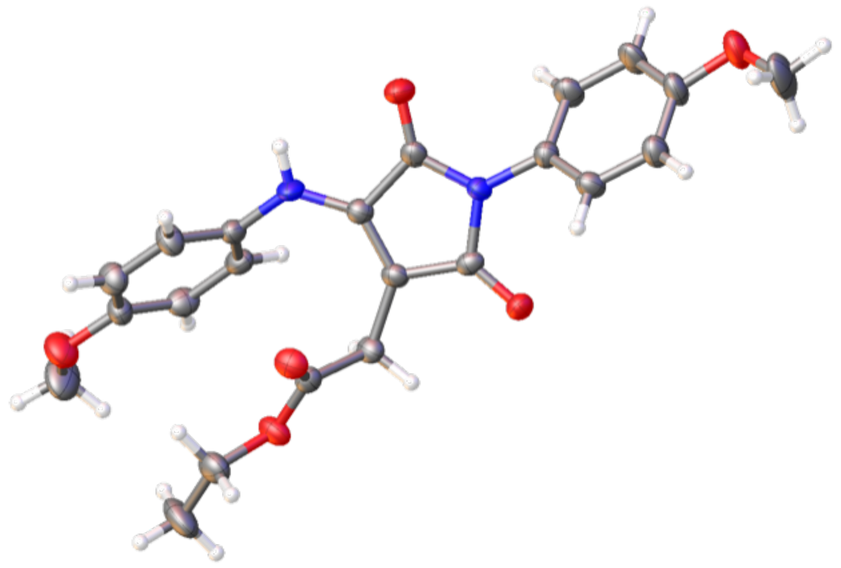

Figure S3. Crystal structure of **4**. Thermal ellipsoids are shown at 30% probability.

Table S6. Crystal data for side product **4**. *Crystallization*: A solution **4** in $CHCl_3$ was covered by a layer of EtOH. After 30 days, single crystals suitable for X-ray analysis were obtained.

Chemical formula	$C_{22}H_{22}N_2O_6$	
Formula weight	410.41	
Collection temperature /K	150	
Crystal size /mm^3	0.35 x 0.26 x 0.18	
Wavelength /Å	0.71073	
Crystal system	Orthorhombic	
Space group	$P2_12_12_1$	
Unit cell dimensions:	a = 7.3725(5) Å	α = 90 °
	b = 16.0734(10) Å	β = 90 °
	c = 34.672(2) Å	γ = 90 °
Unit cell volume /Å^3	4108.7(5) Å^3	
Z, Calculated density /Mg/m^3	8, 1.327	
Radiation type	MoKα	
Absorption coefficient, m/mm^{-1}	0.098	
No. reflections collected / unique	74220/7207	
R_{int}	0.2037	
Completeness to theta = 25.000 /%	99.6	
Data / restraints / parameters	7207 / 0 / 547	
Goodness of fit on F^2	1.013	
Final R indices $(I > 2\sigma(I))$	R_1 = 0.0751, wR_2 = 0.1258	
R indices *(all data)*	R_1 = 0.1680, wR_2 = 0.1575	
Absolute structure parameter	0.9(10)	
Largest diff. peak and hole /e-/Å^3	0.174 and -0.192	
CDCC	2352163	

DFT calculations

All QM calculations were performed with Jaguar as implemented in Schrödinger release 2020-3,[S9-10] using default settings except that the SCF accuracy level was set to accurate with a switch to analytical integrals near convergence. Gas phase energy minima and transition state geometries were determined employing density functional theory (DFT) with the M06-2X functional[S11] and 6-31G** basis set. Calculated vibrational frequencies for each transition state gave single imaginary values. Atom coordinates of each calculated species are given in the xyz file available for download as supporting information.

A simplified analogue of compound **1** (compound **1'**) was used as starting material for the calculations. The calculations were performed assuming that the Baeyer-Villiger-type oxidation takes place before the epoxidation, but opposite order is also possible. An alternative reaction pathway where an initial epoxidation is followed by a rearrangement and lastly the Baeyer-Villiger oxidation was excluded as that pathway includes a rearrangement into a β-lactam, which according to DFT calculations and orbital overlap is highly unlikely.

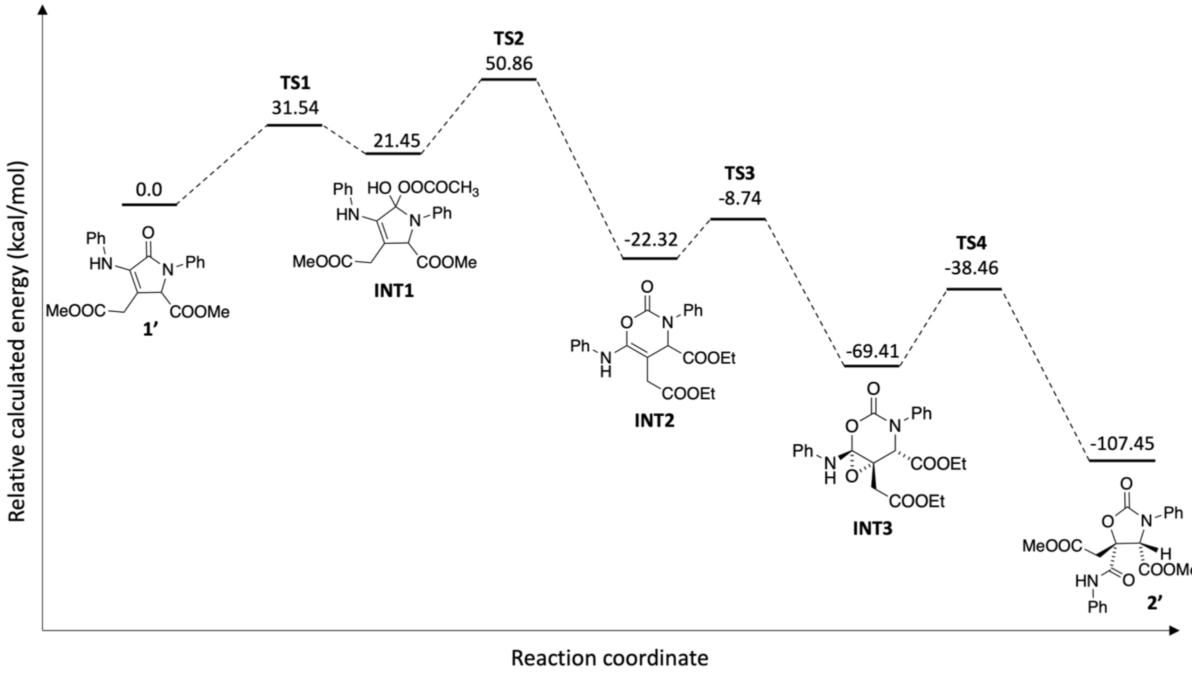

Figure S4. Reaction coordinate diagram for the proposed mechanism for the transformation of α,β-unsaturated γ-lactam **1'** into oxazolidinone **2'**. The energies are given relative to the starting material (**1'**). Calculated energies of formic acid and performic acid were added to make the reactions isodesmic.

The epoxidation could take place on either face of dihydropyranone **INT2**. Based on the known relative stereochemistry of product **2'**, only one of the epoxides will rearrange into the product. A comparison of the calculated energies of the transition states of the two possible epoxidations showed that the TS of the undesired epoxidation is 1.1 kcal/mol higher in energy then the desired epoxidation (**TS3**), most likely due to stabilizing interactions between the peracid and the COOEt substituent in the TS of the desired epoxidation.

Table S7. Structures and calculated energies of intermediates and transition states. Dashed lines indicated forming or breaking bonds.

**1’**<br>Energy: -1751.939763 Ha<br>Energy (**1’** + performic acid): -2016.717807 Ha	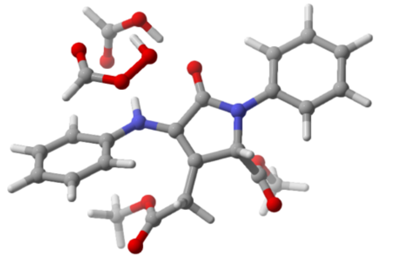
**TS1**<br>Energy: -1751.889493 Ha<br>Energy (**TS1** + performic acid): -2016.667537 Ha	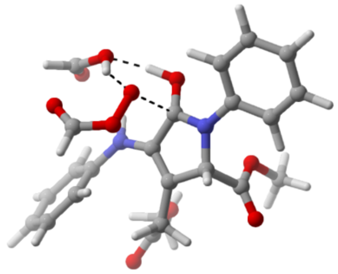
**INT1** (Criegee intermediate)<br>Energy: -1751.905576 Ha<br>Energy (**INT1 +** performic acid): -2016.68362 Ha	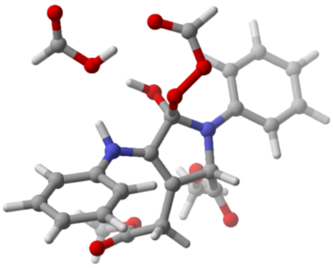
**TS2**<br>Energy: -1751.858712 Ha<br>Energy (**TS2** + performic acid): -2016.636756 Ha	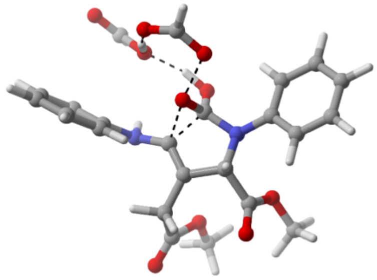
**INT2 (**Baeyer-Villiger product)<br>Energy: -1372.624906 Ha<br>Energy (**INT2** + 2 formic acid + performic acid): -2016.753382 Ha	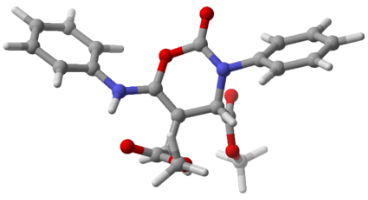
**TS3**<br>Energy: -1637.381308 Ha<br>Energy (**TS3** + 2 formic acid): -2016.73174 Ha	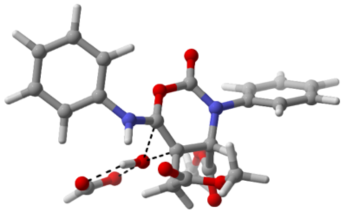

**INT3** (epoxidation product)<br>Energy: -1447.80277 Ha<br>Energy (**INT3** + 3 formic acid): -2016.828418 Ha	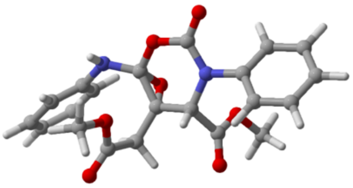
**TS4**<br>Energy: -1447.753453 Ha<br>Energy (**TS4** + 3 formic acid): -2016.779101 Ha	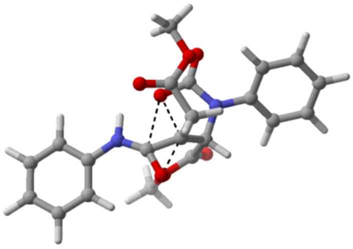
**2'** (rearrangement product)<br>Energy: -1447.863395 Ha<br>Energy (**2'** + 3 formic acid): -2016.889043 Ha	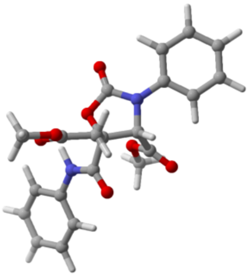

Copies of NMR spectra

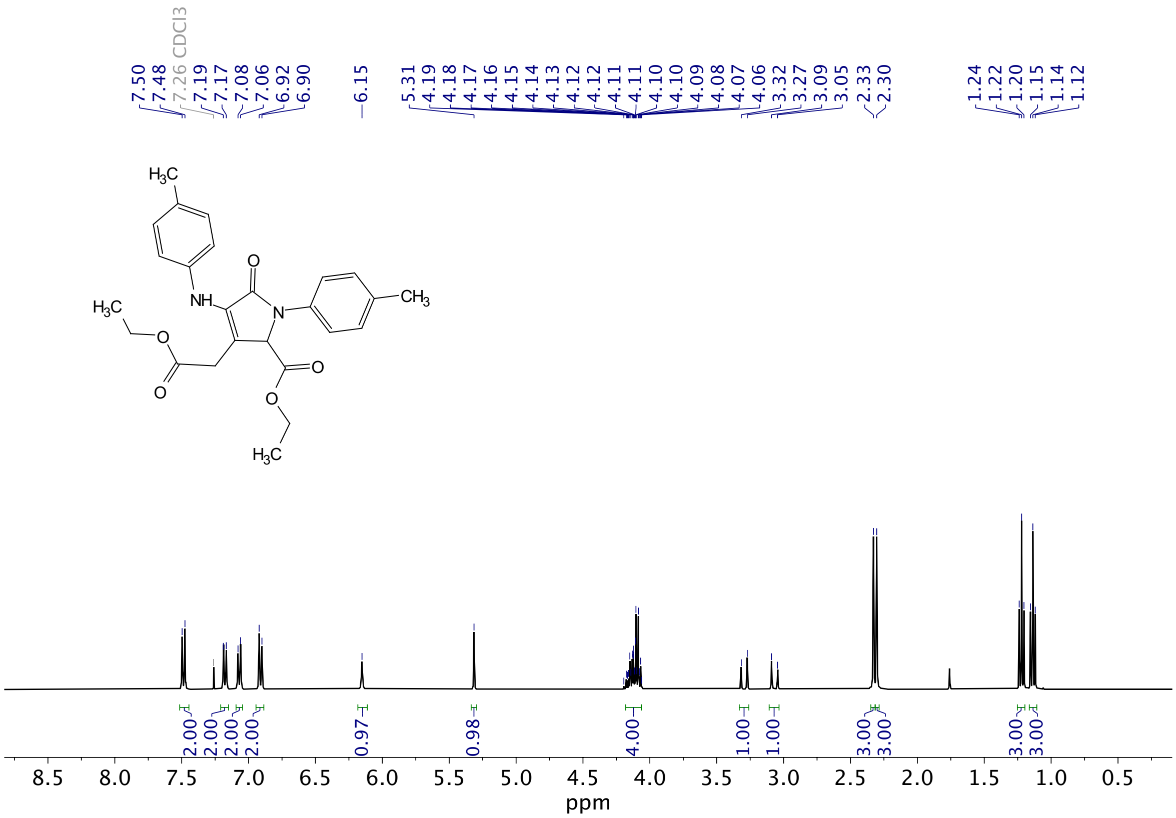

Figure S5. ^{1}H NMR spectrum (400 MHz, $CDCl_3$) of compound (±)-**5**.

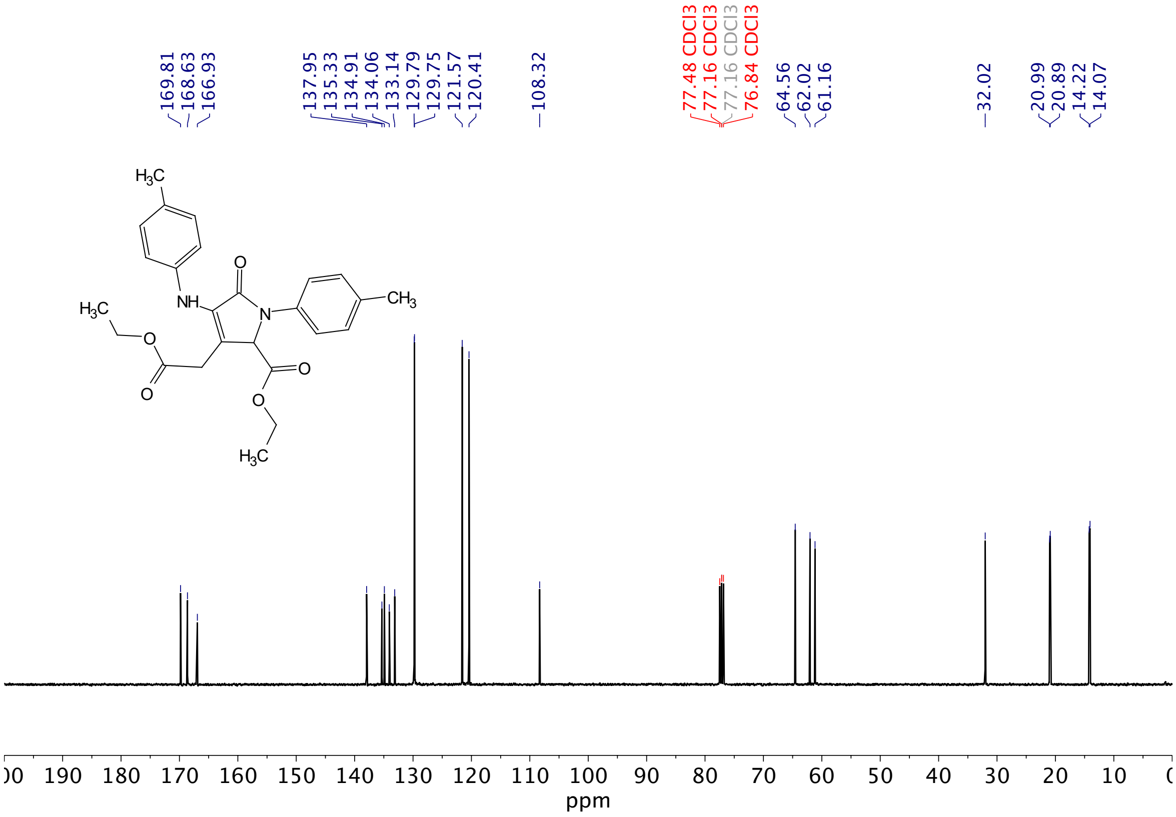

Figure S6. ^{13}C{^{1}H} NMR spectrum (101 MHz, $CDCl_3$) of compound (±)-**5**.

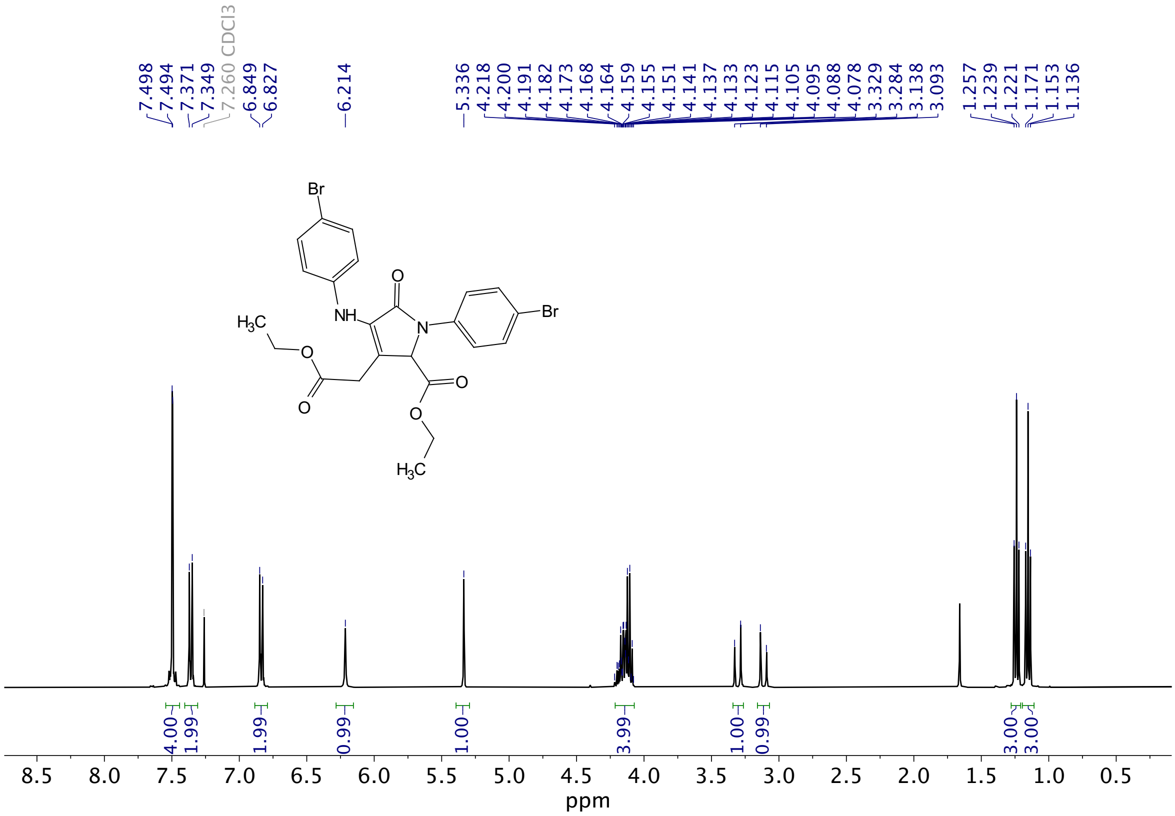

Figure S7. ^{1}H NMR spectrum (400 MHz, $CDCl_3$) of compound (±)-**6**.

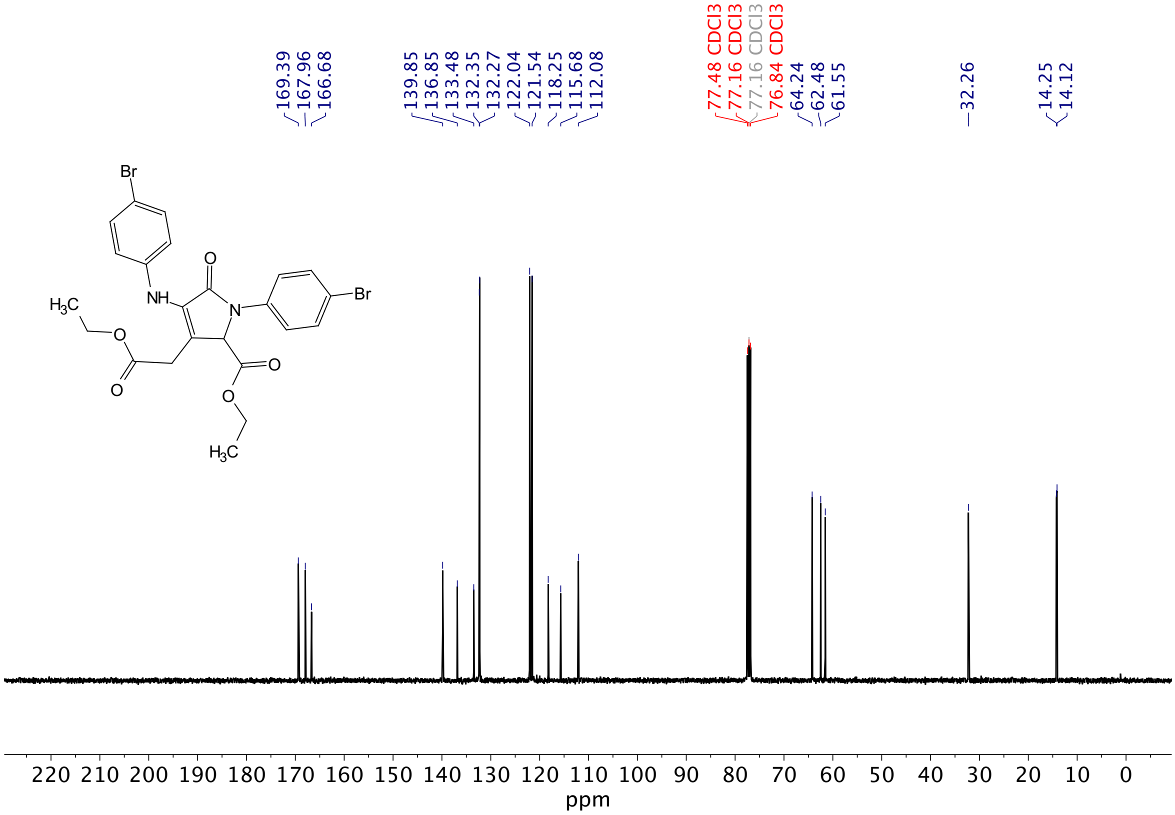

Figure S8. $^{13}C\{^{1}H\}$ NMR spectrum (101 MHz, $CDCl_3$) of compound (±)-**6**.

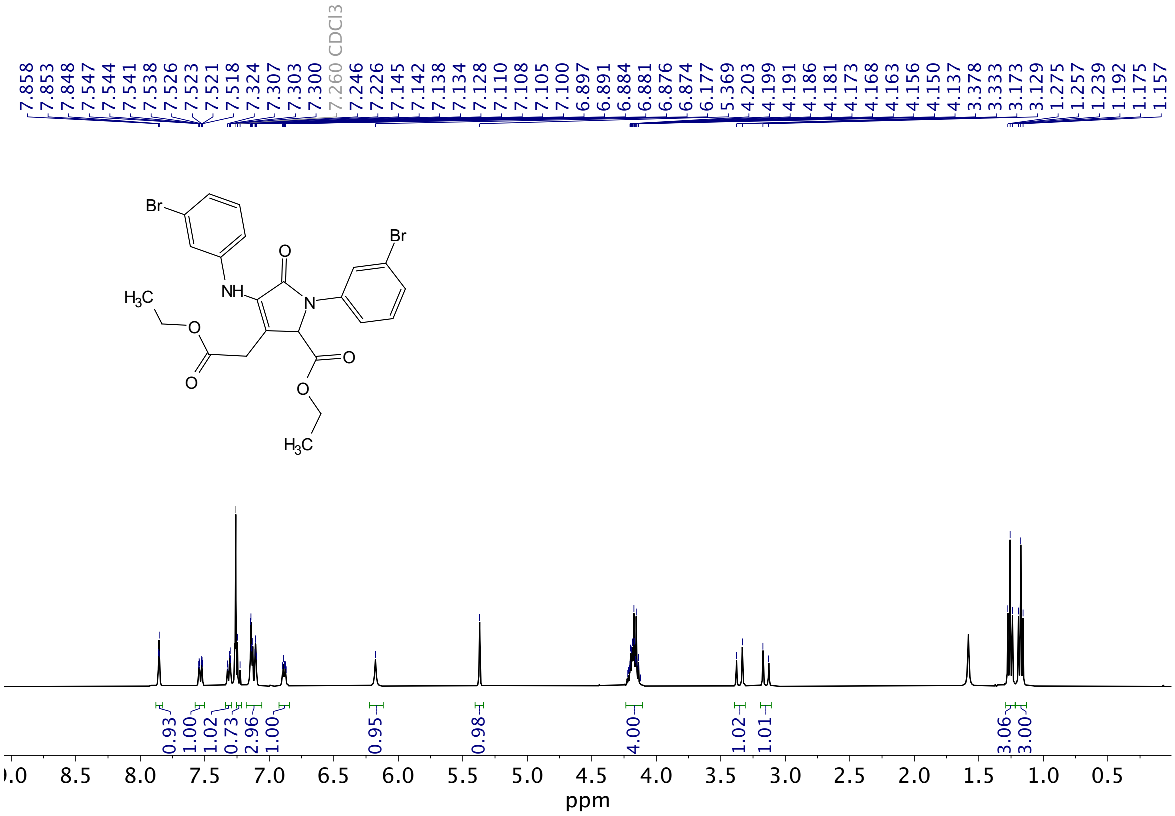

Figure S9. ^{1}H NMR spectrum (400 MHz, $CDCl_3$) of compound (±)-**7**.

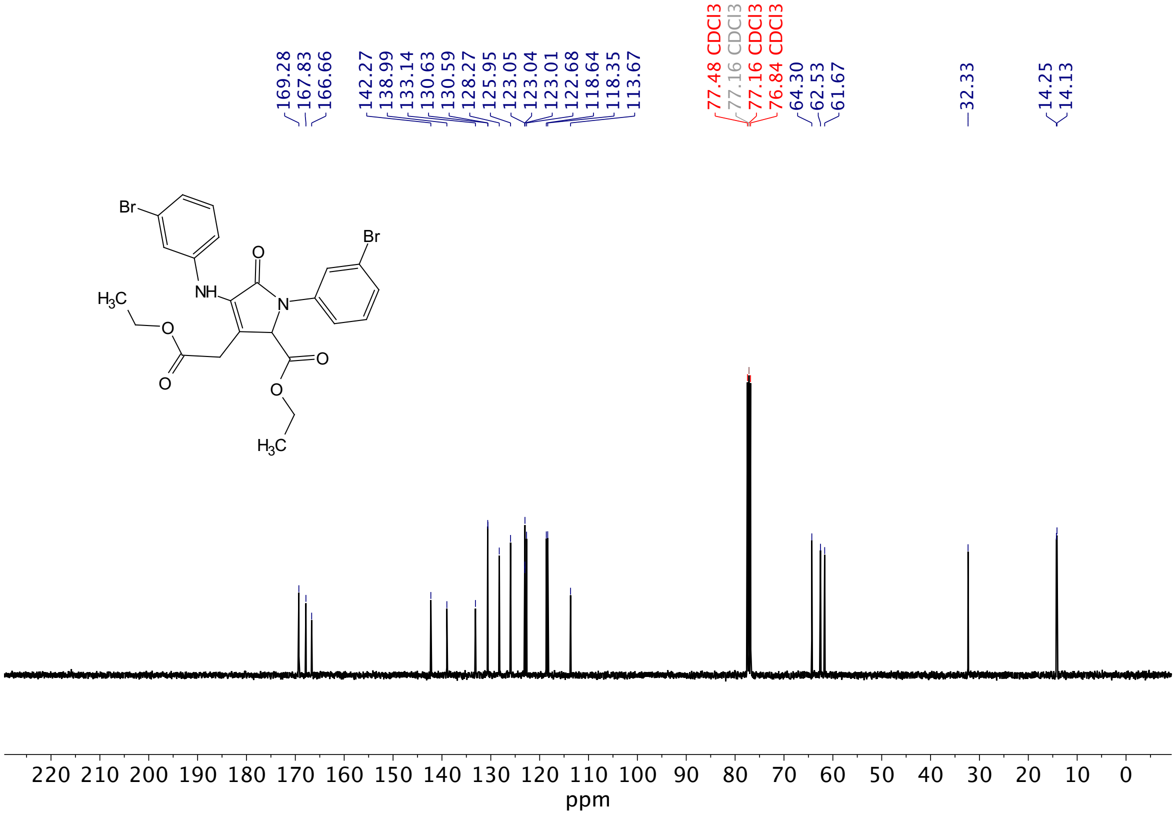

Figure S10. $^{13}C\{^{1}H\}$ NMR spectrum (101 MHz, $CDCl_3$) of compound (±)-**7**.

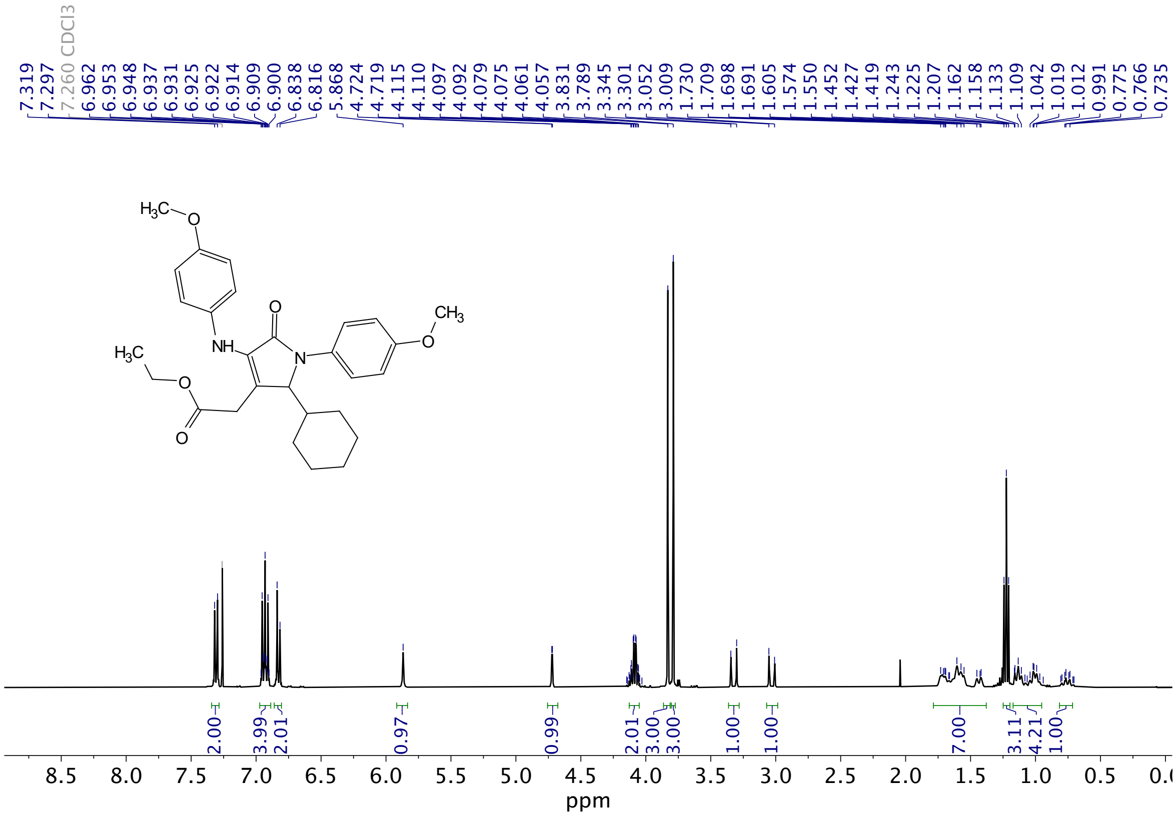

Figure S11. ^{1}H NMR spectrum (400 MHz, $CDCl_3$) of compound (±)-**8**.

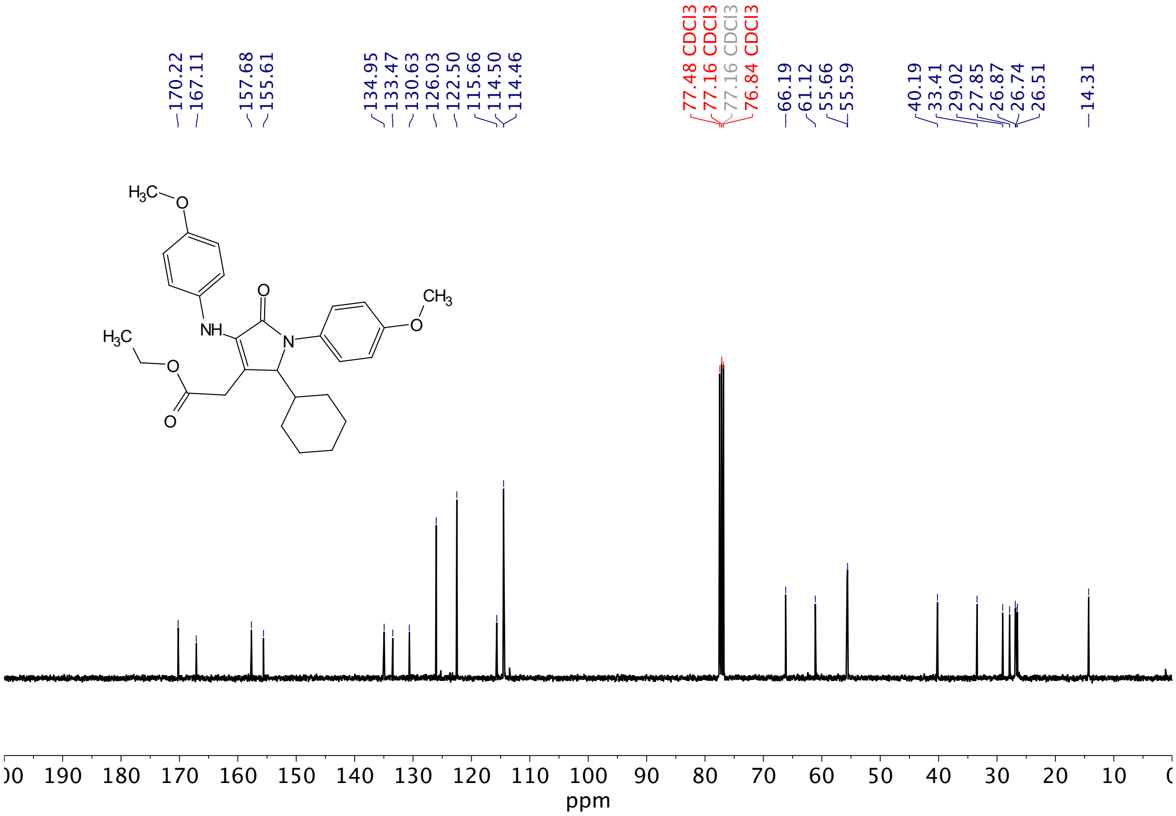

Figure S12. $^{13}C\{^{1}H\}$ NMR spectrum (101 MHz, $CDCl_3$) of compound (±)-**8**.

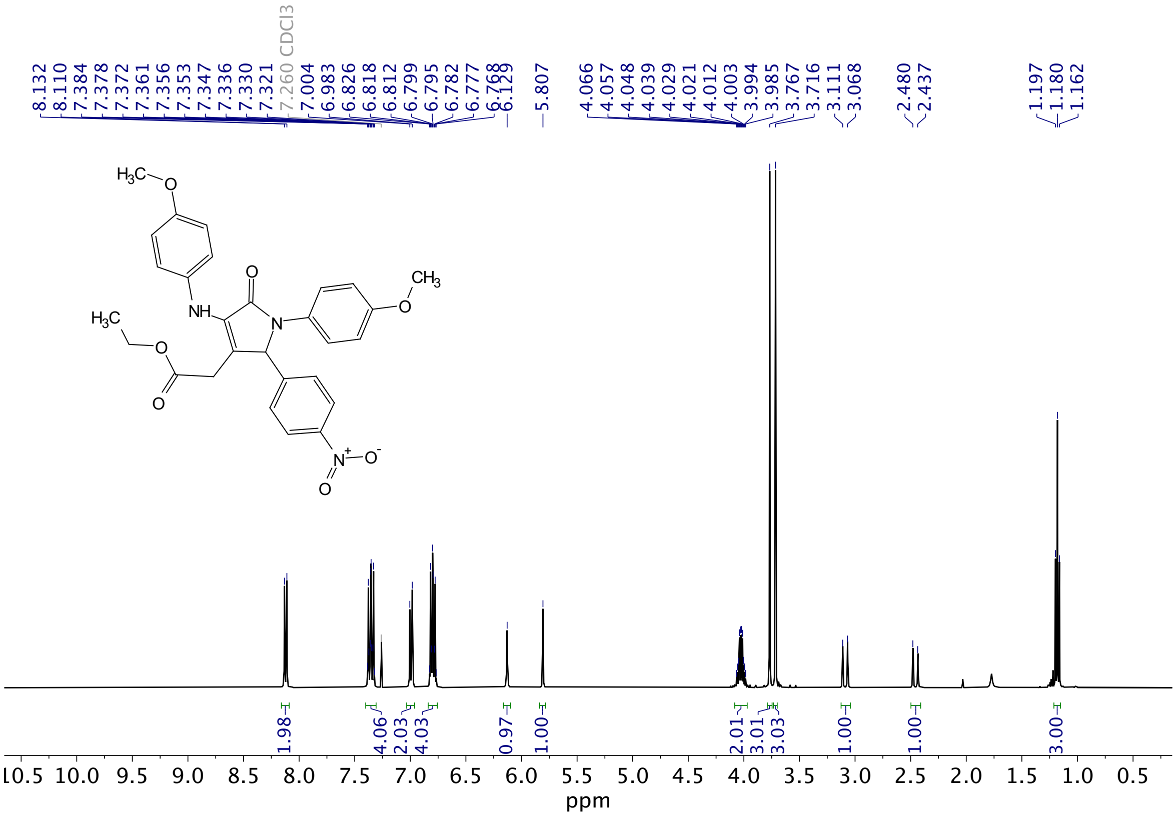

Figure S13. ^{1}H NMR spectrum (400 MHz, $CDCl_3$) of compound (±)-**9**.

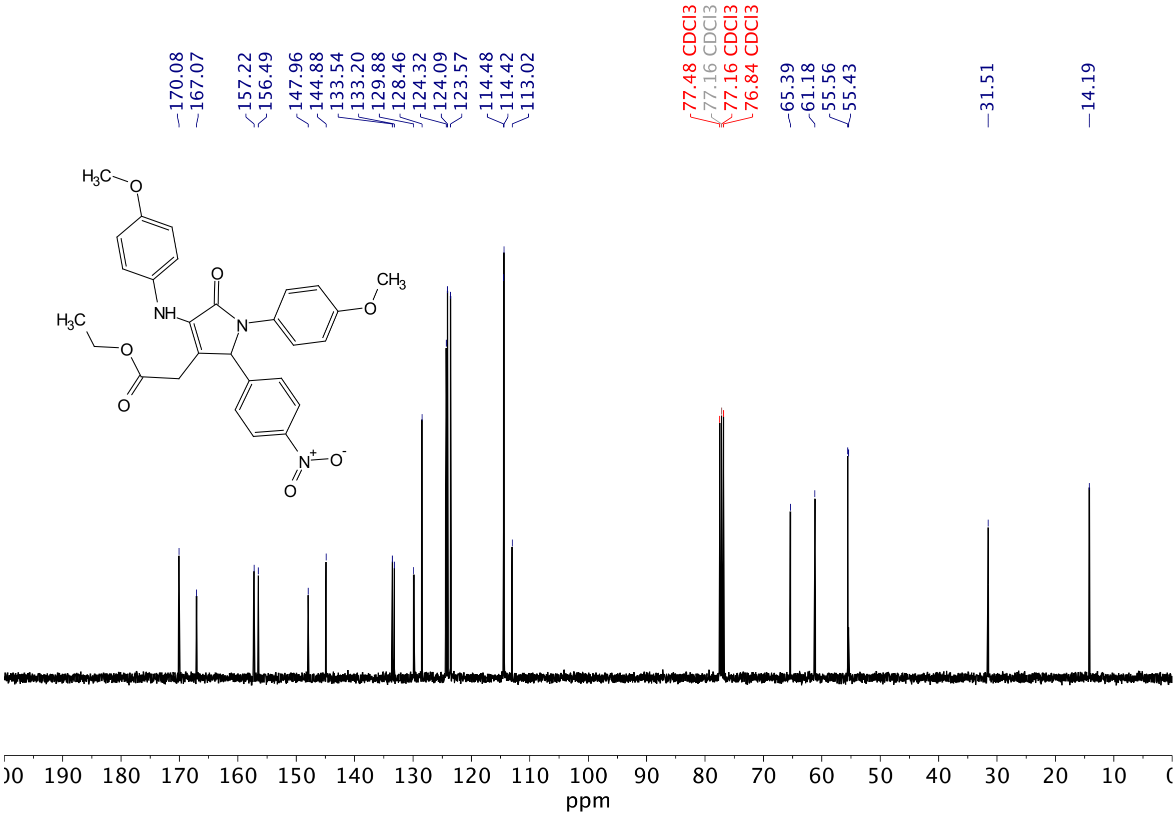

Figure S14. $^{13}C\{^{1}H\}$ NMR spectrum (101 MHz, $CDCl_3$) of compound (±)-**9**.

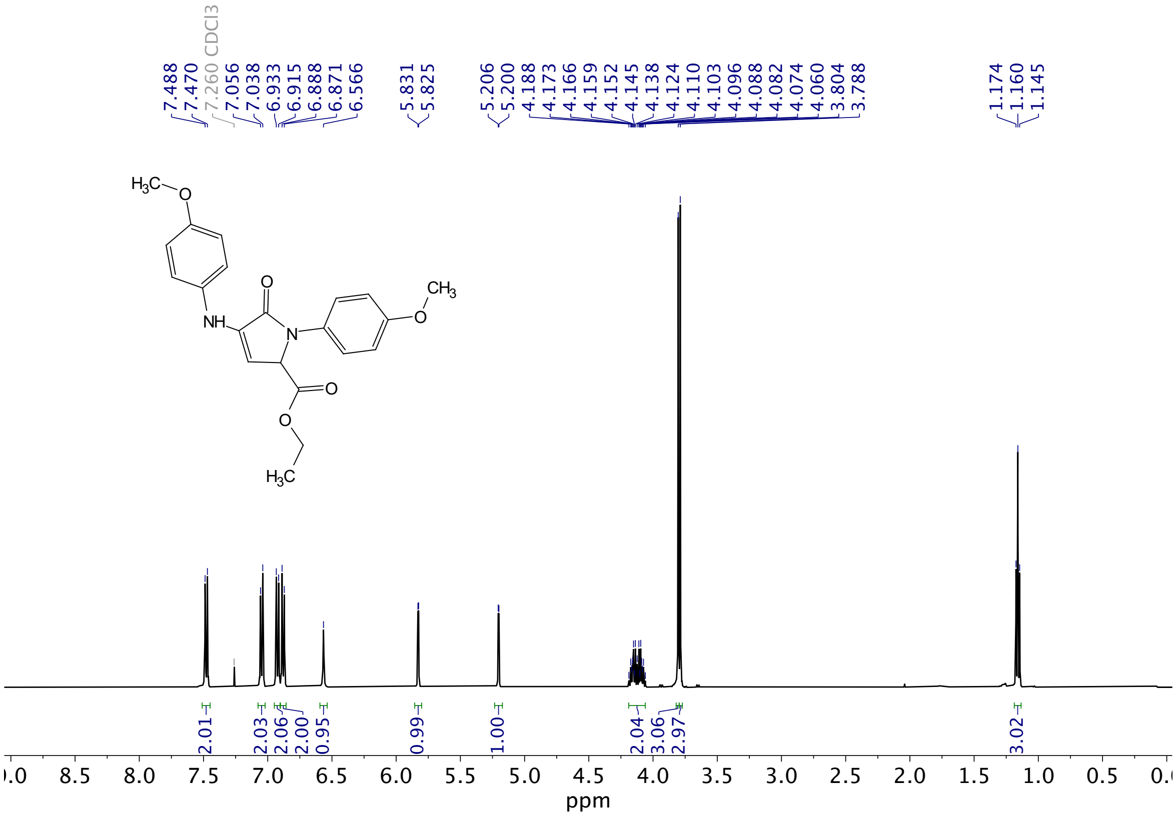

Figure S15. ^{1}H NMR spectrum (500 MHz, $CDCl_3$) of compound (±)-**10**.

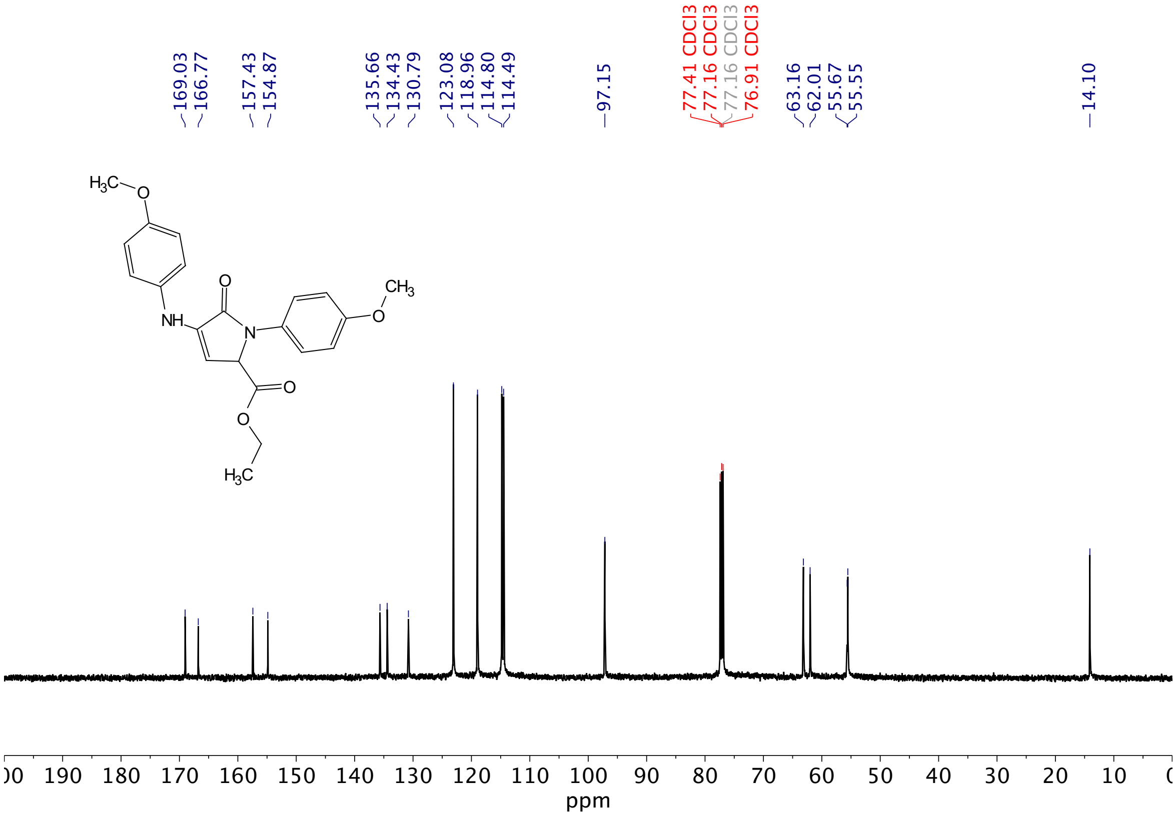

Figure S16. $^{13}C\{^{1}H\}$ NMR spectrum (126 MHz, $CDCl_3$) of compound (±)-**10**.

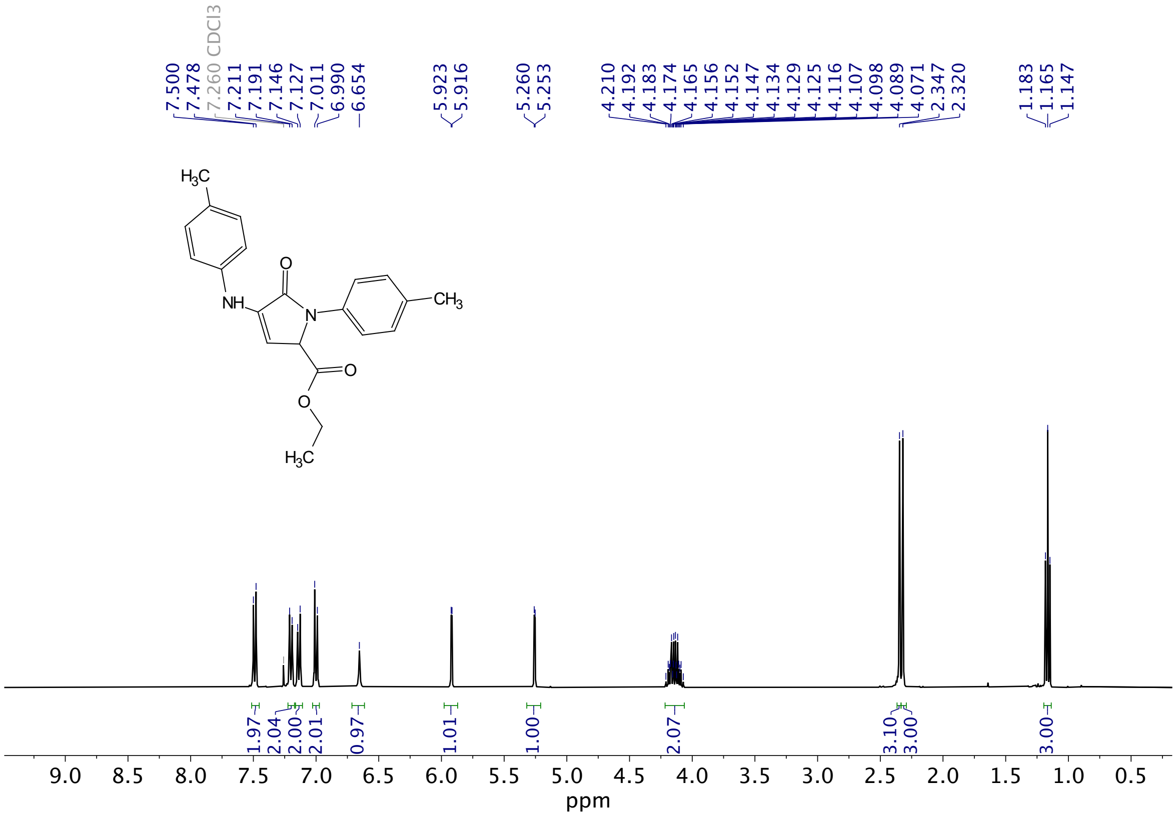

Figure S17. ^{1}H NMR spectrum (400 MHz, $CDCl_3$) of compound (±)-**11**.

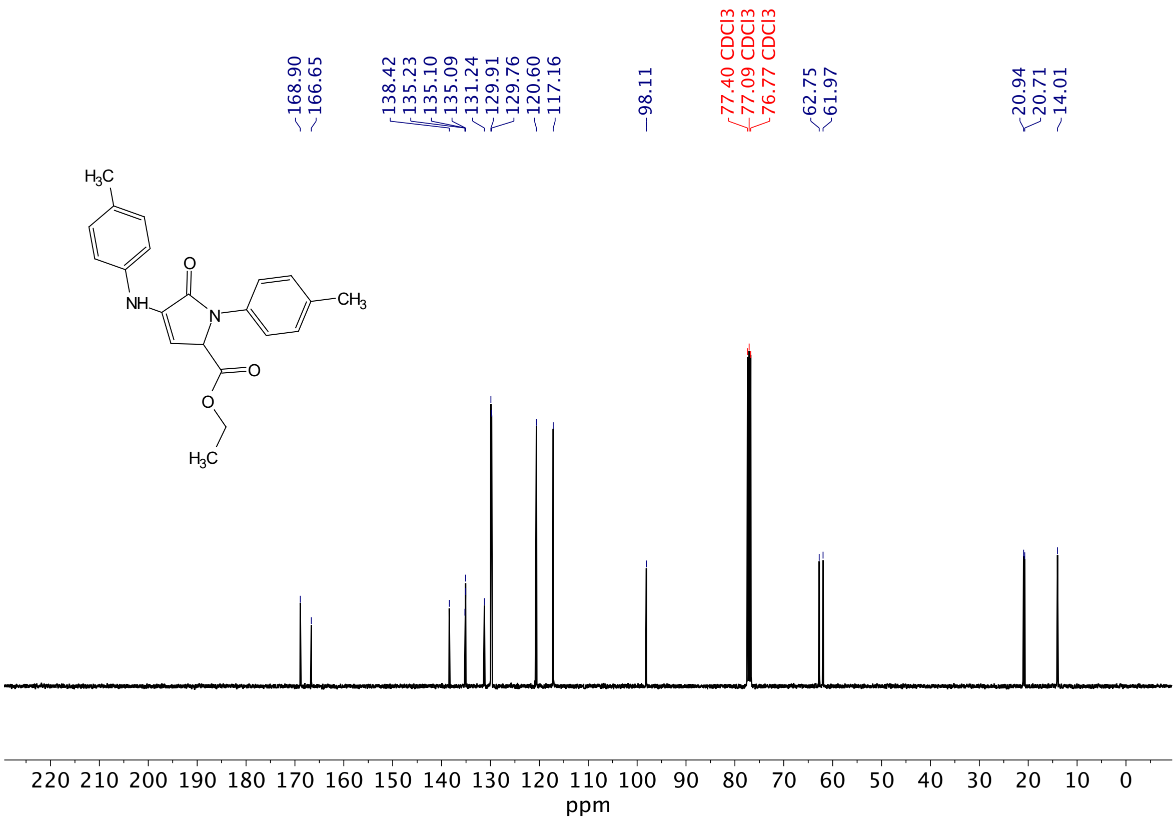

Figure S18. $^{13}C\{^{1}H\}$ NMR spectrum (101 MHz, $CDCl_3$) of compound (±)-**11**.

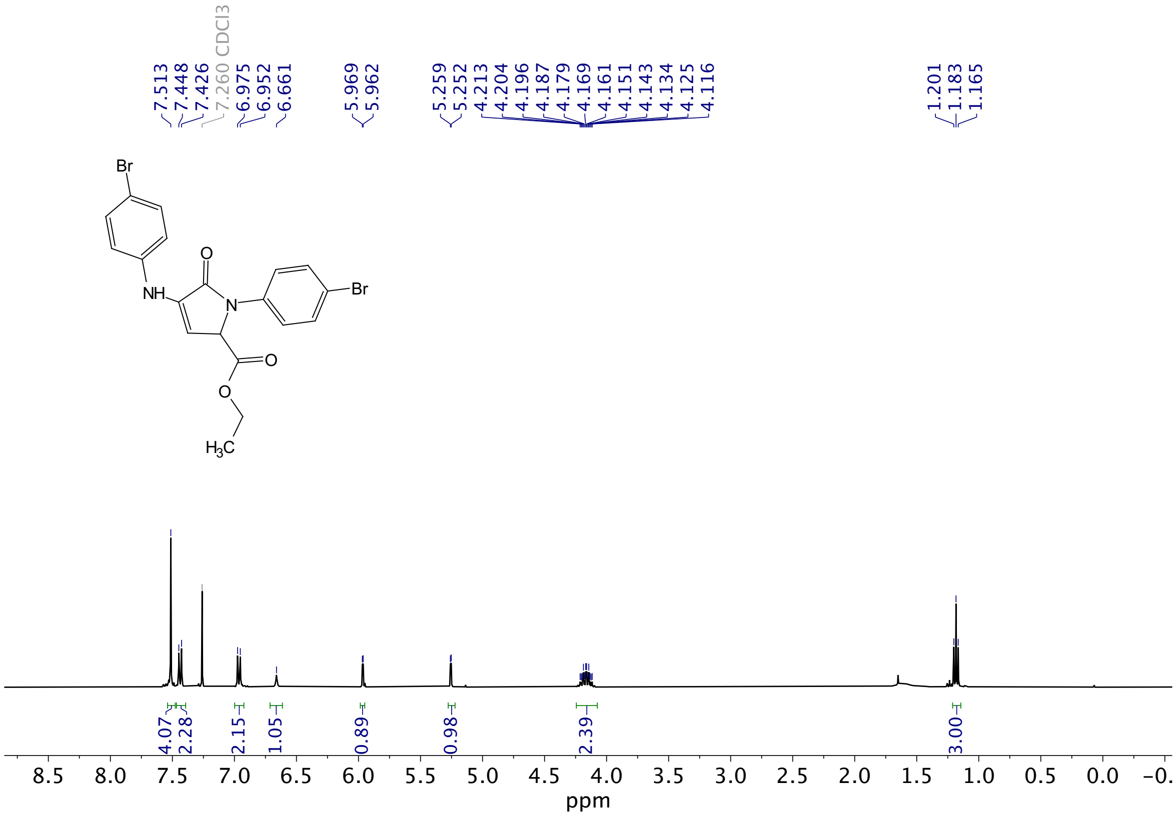

Figure S19. ^{1}H NMR spectrum (400 MHz, $CDCl_3$) of compound (±)-**12**.

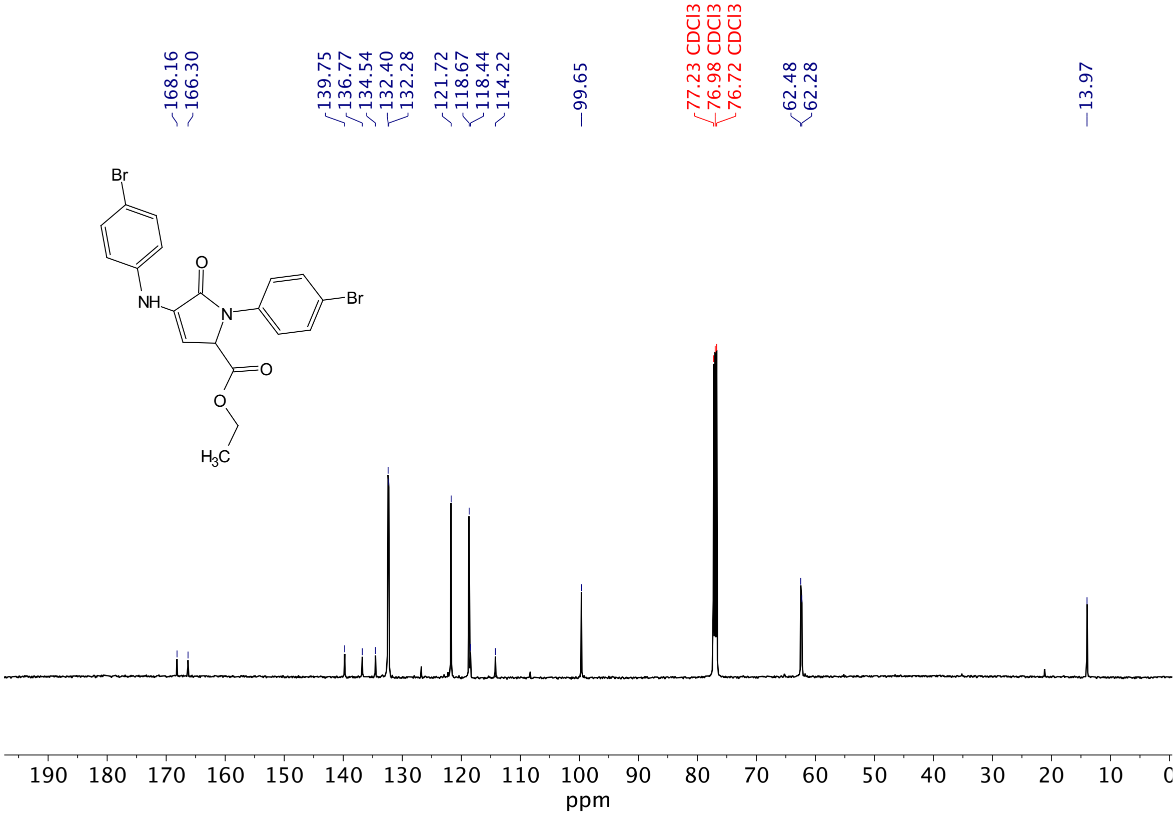

Figure S20. $^{13}C\{^{1}H\}$ NMR spectrum (126 MHz, $CDCl_3$) of compound (±)-**12**. Sample measured at 40 °C due to low solubility in chloroform.

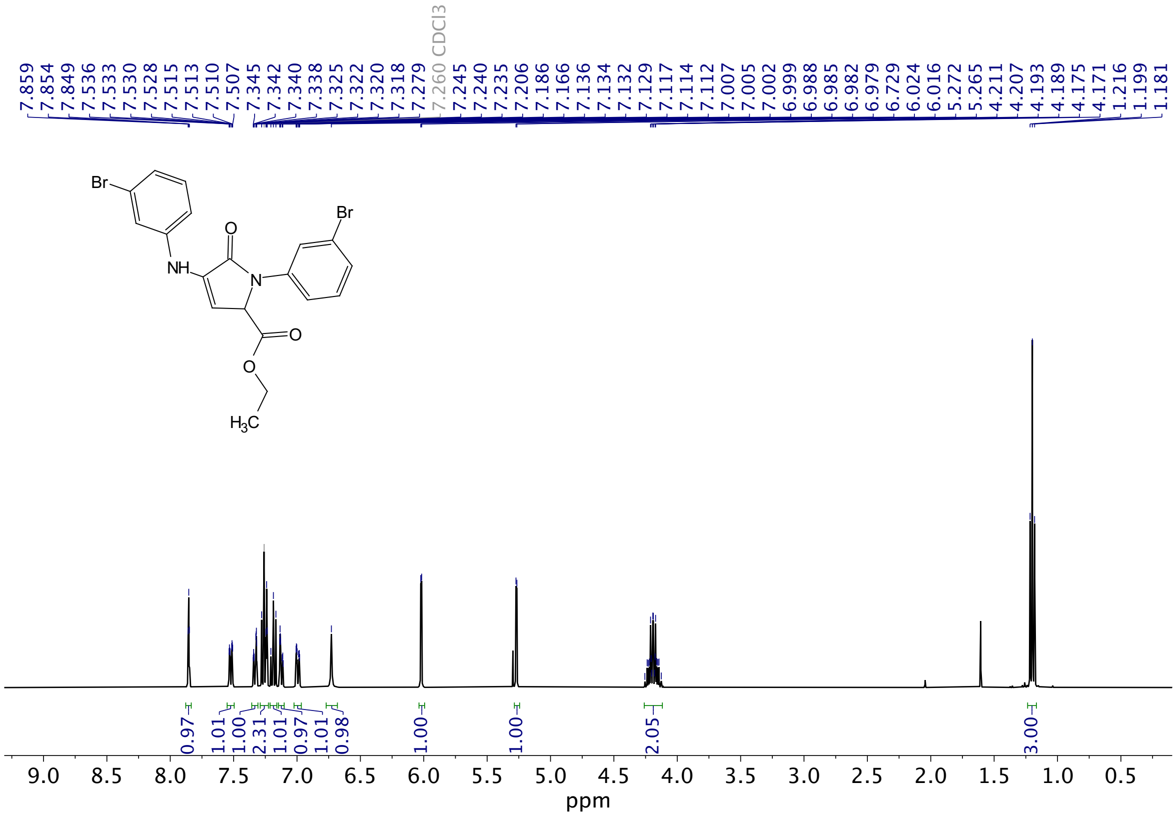

Figure S21. ^{1}H NMR spectrum (400 MHz, $CDCl_3$) of compound (±)-**13**.

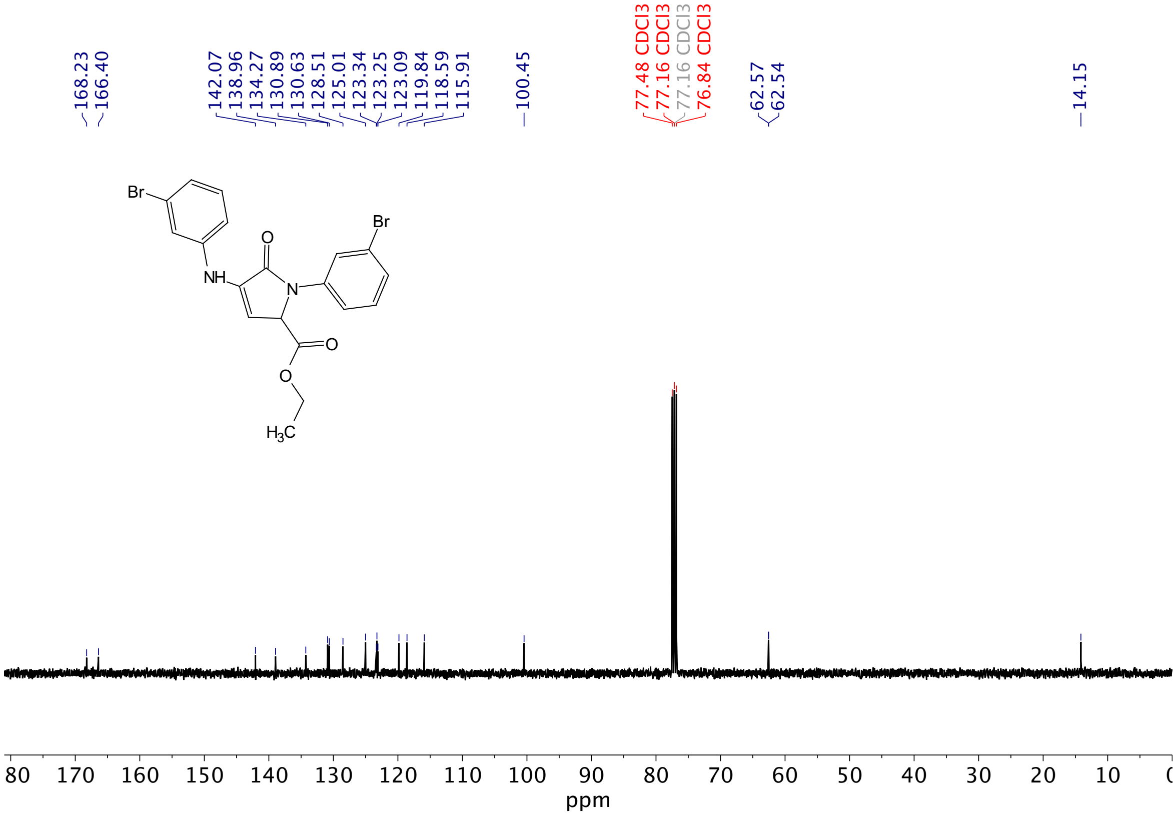

Figure S22. $^{13}C\{^{1}H\}$ NMR spectrum (101 MHz, $CDCl_3$) of compound (±)-**13**.

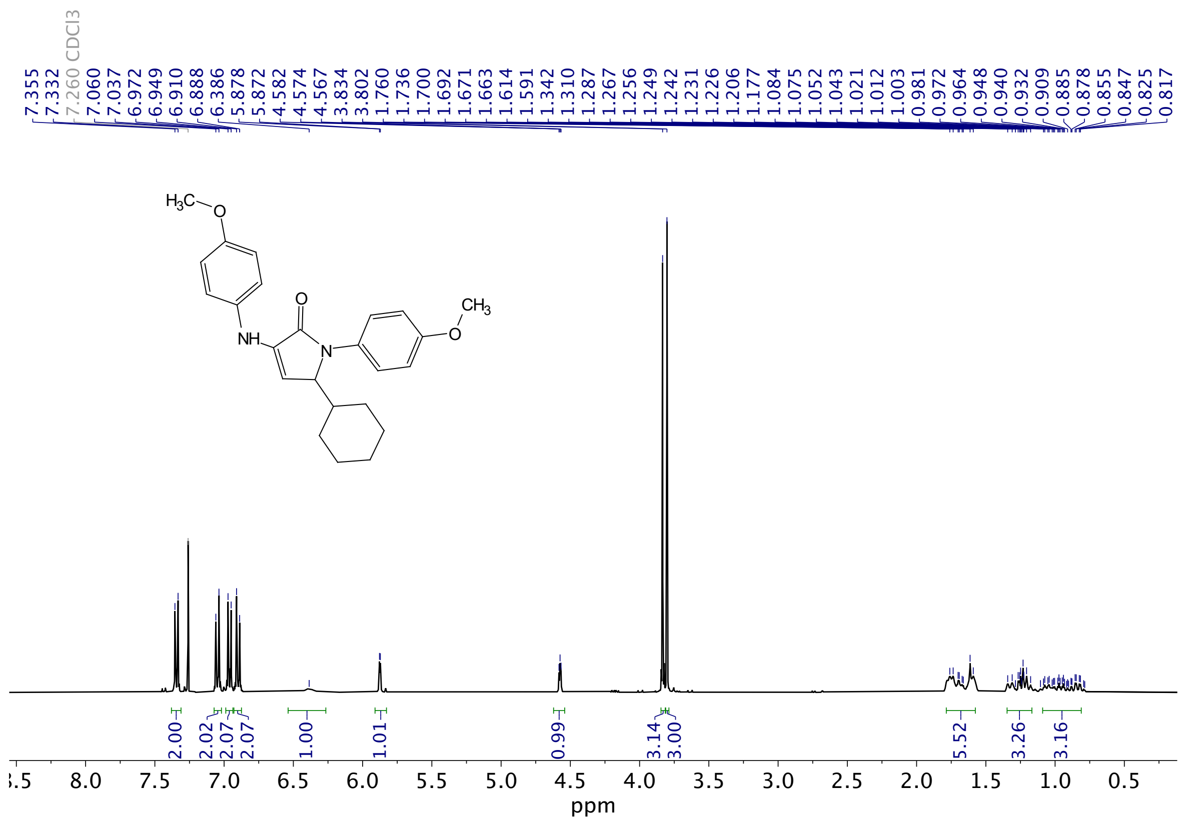

Figure S23. ^{1}H NMR spectrum (400 MHz, $CDCl_3$) of compound (±)-**14**.

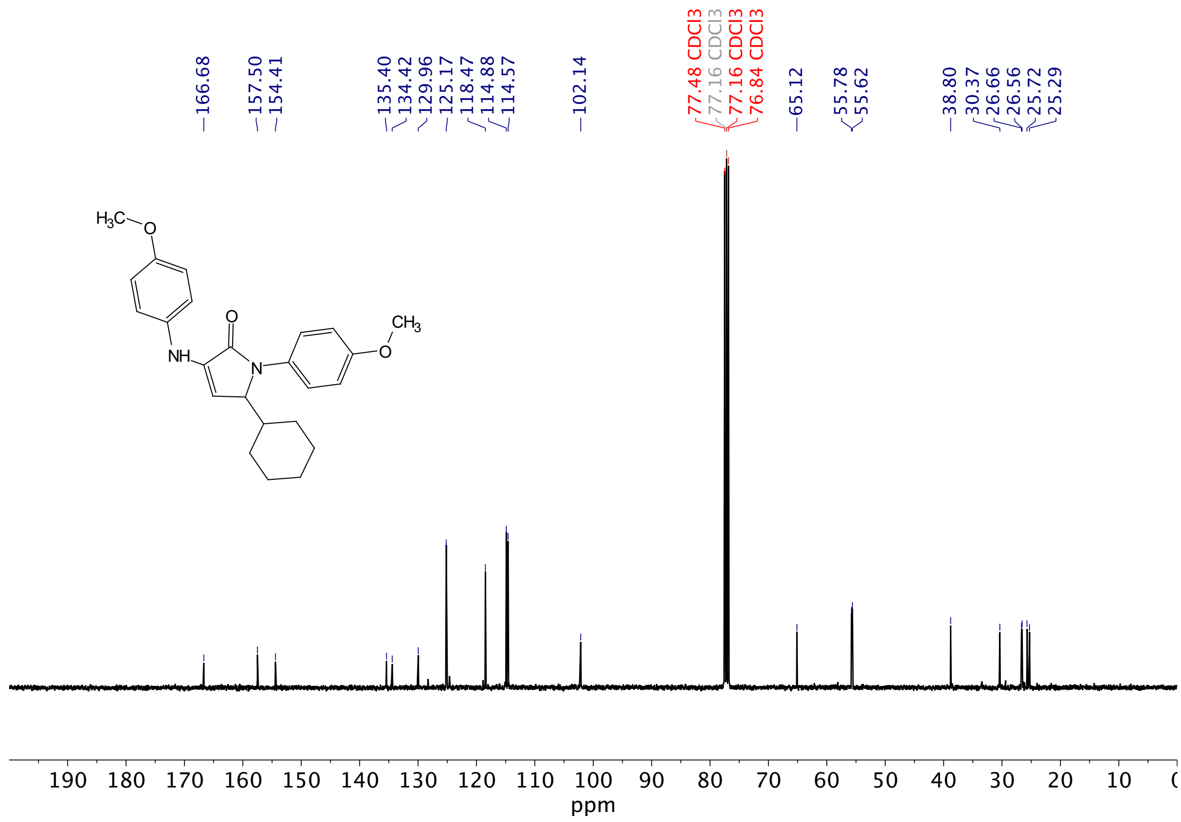

Figure S24. $^{13}C\{^{1}H\}$ NMR spectrum (100 MHz, $CDCl_3$) of compound (±)-**14**.

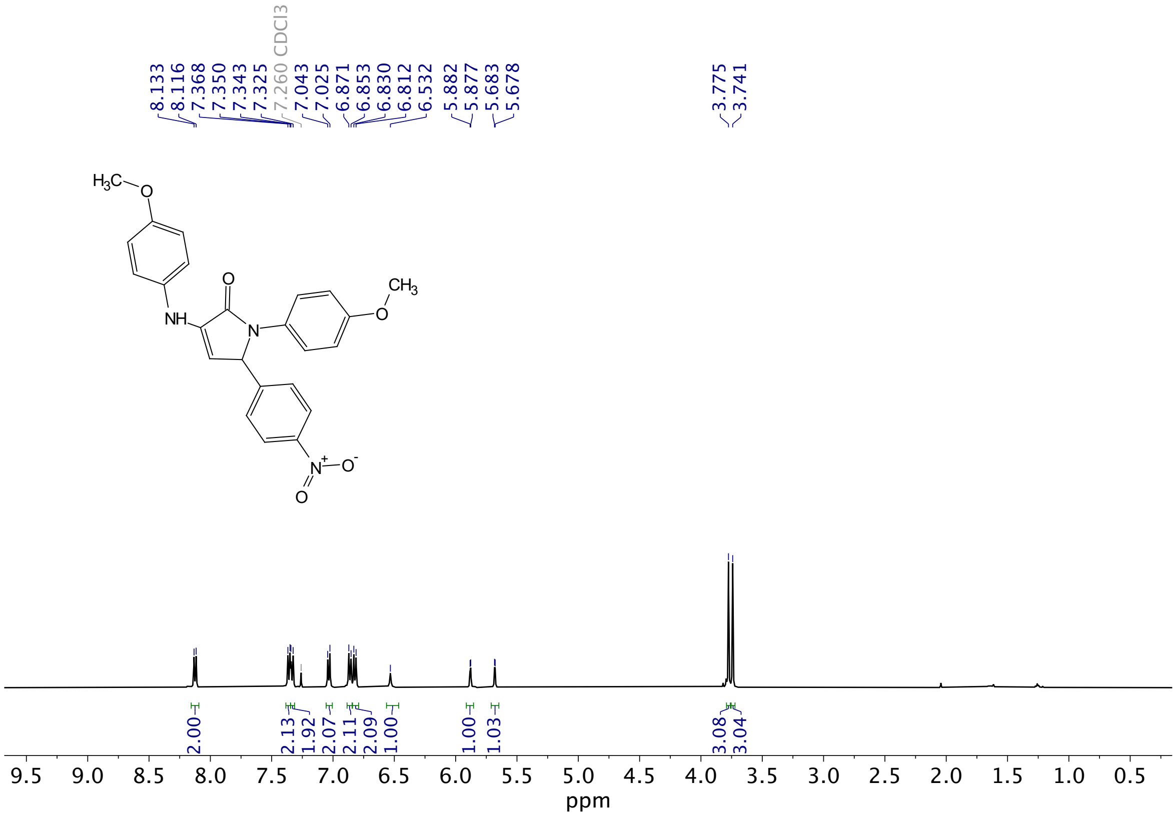

Figure S25. ^{1}H NMR spectrum (500 MHz, $CDCl_3$) of compound (±)-**15**.

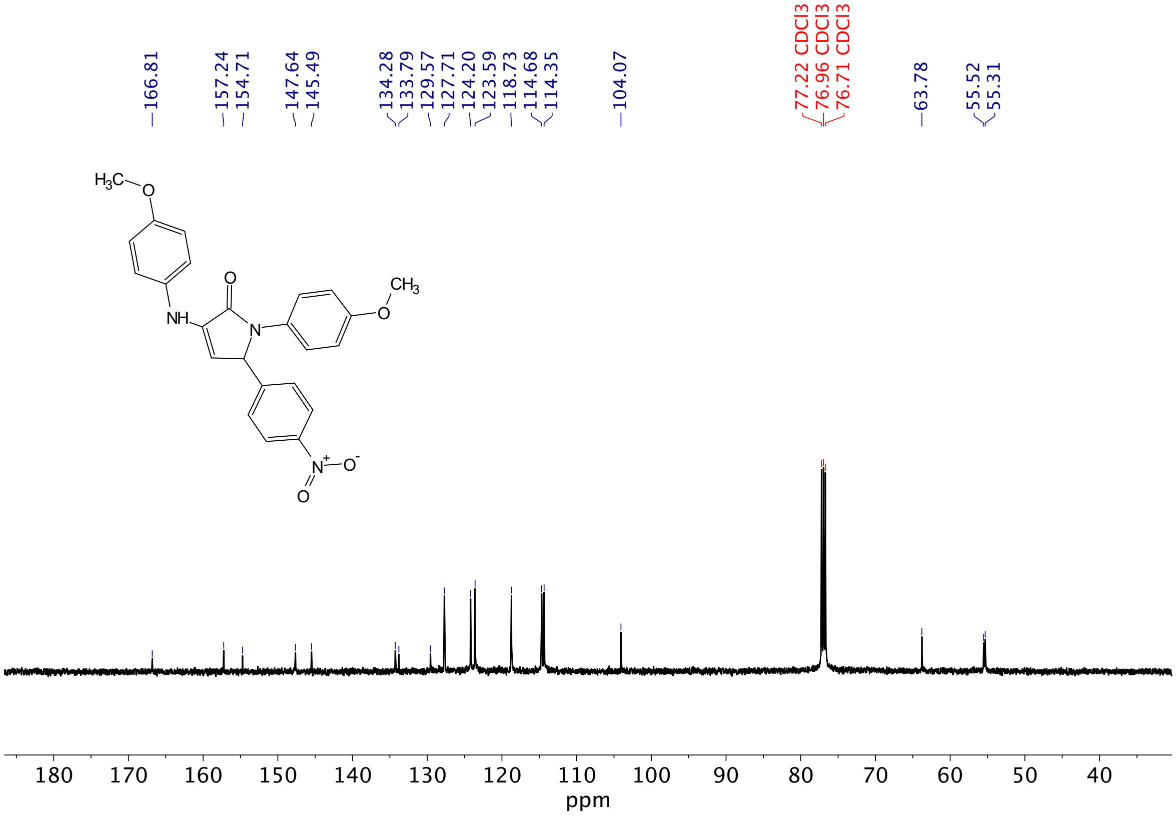

Figure S26. $^{13}C\{^{1}H\}$ NMR spectrum (126 MHz, $CDCl_3$) of compound (±)-**15**.

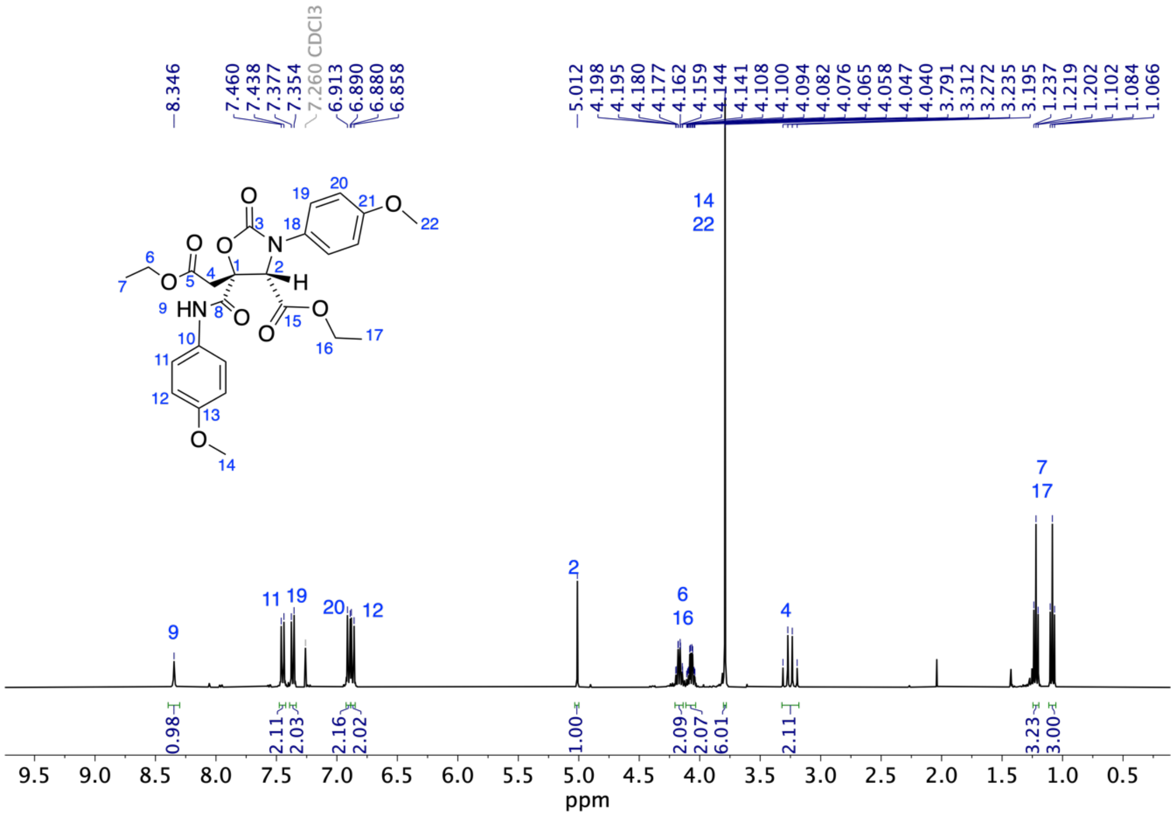

Figure S27. ^{1}H NMR spectrum (400 MHz, $CDCl_3$) of compound (±)-**2**.

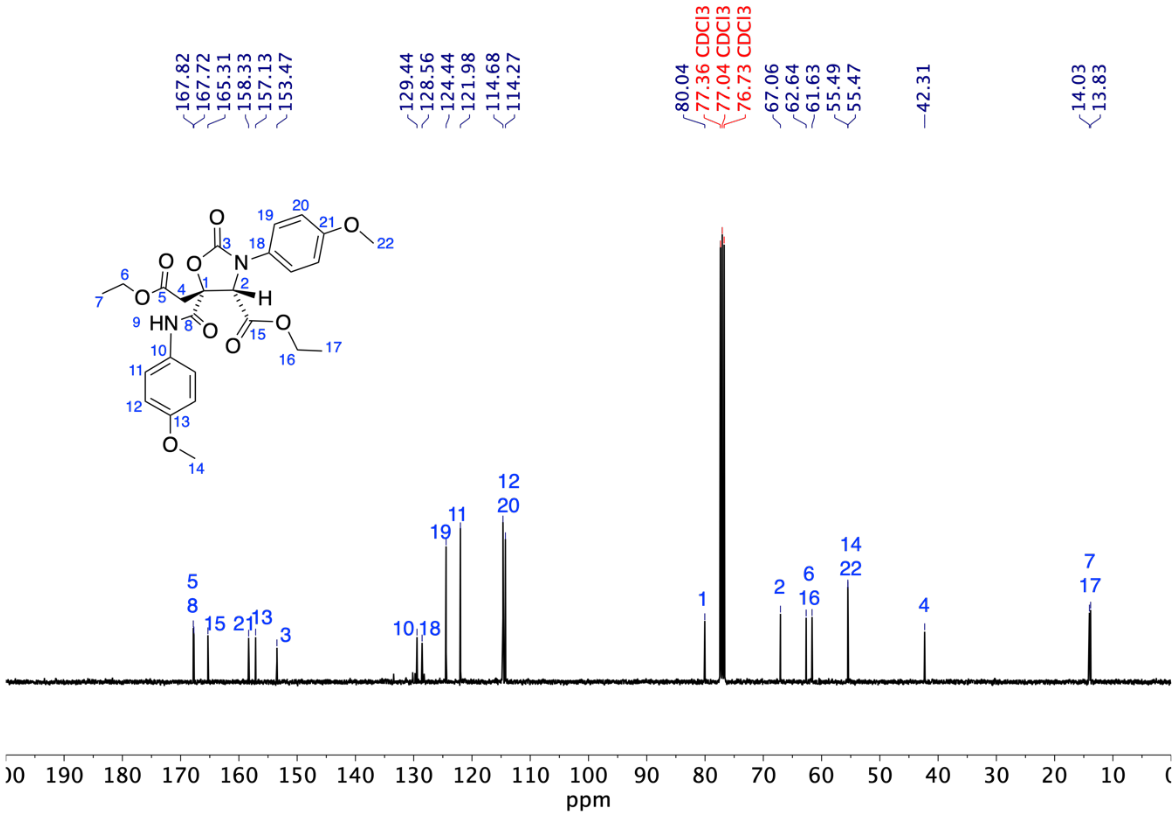

Figure S28. $^{13}C\{^{1}H\}$ NMR spectrum (101 MHz, $CDCl_3$) of compound (±)-**2**.

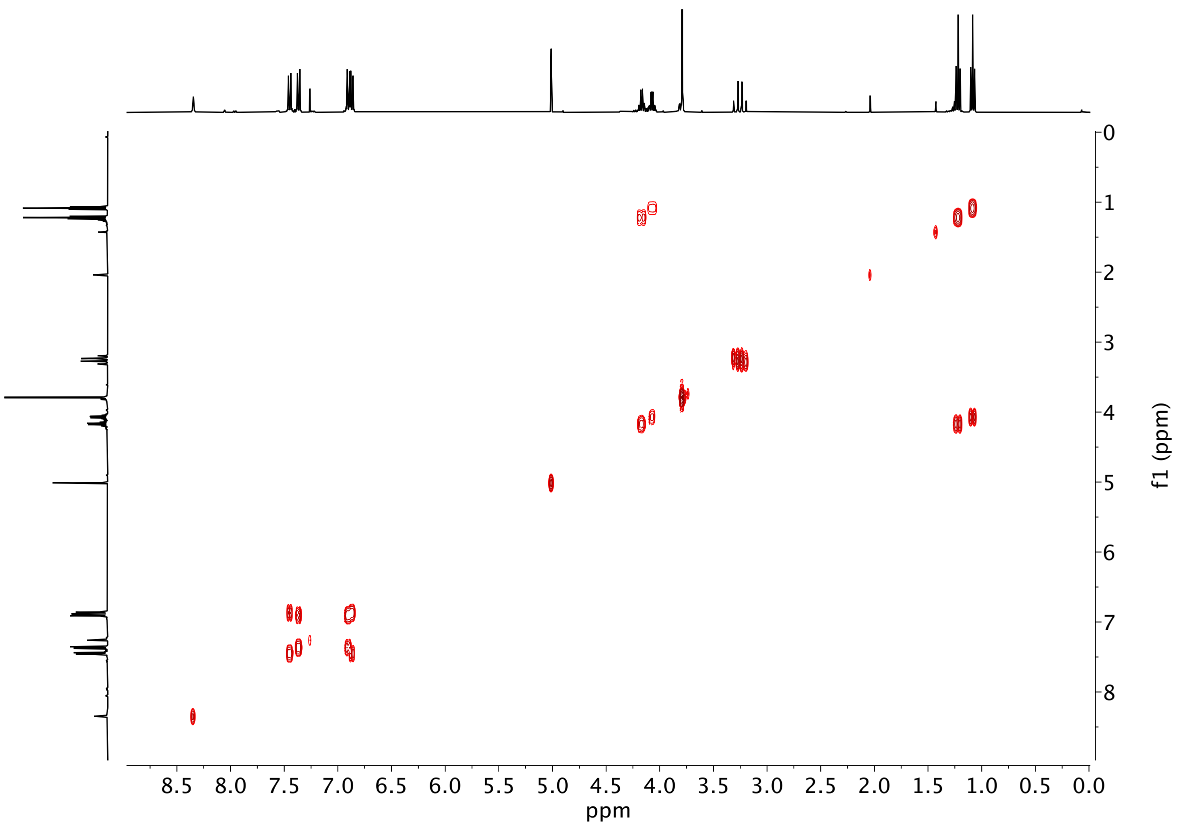

Figure S29. COSY spectrum of (±)-**2** in $CDCl_3$.

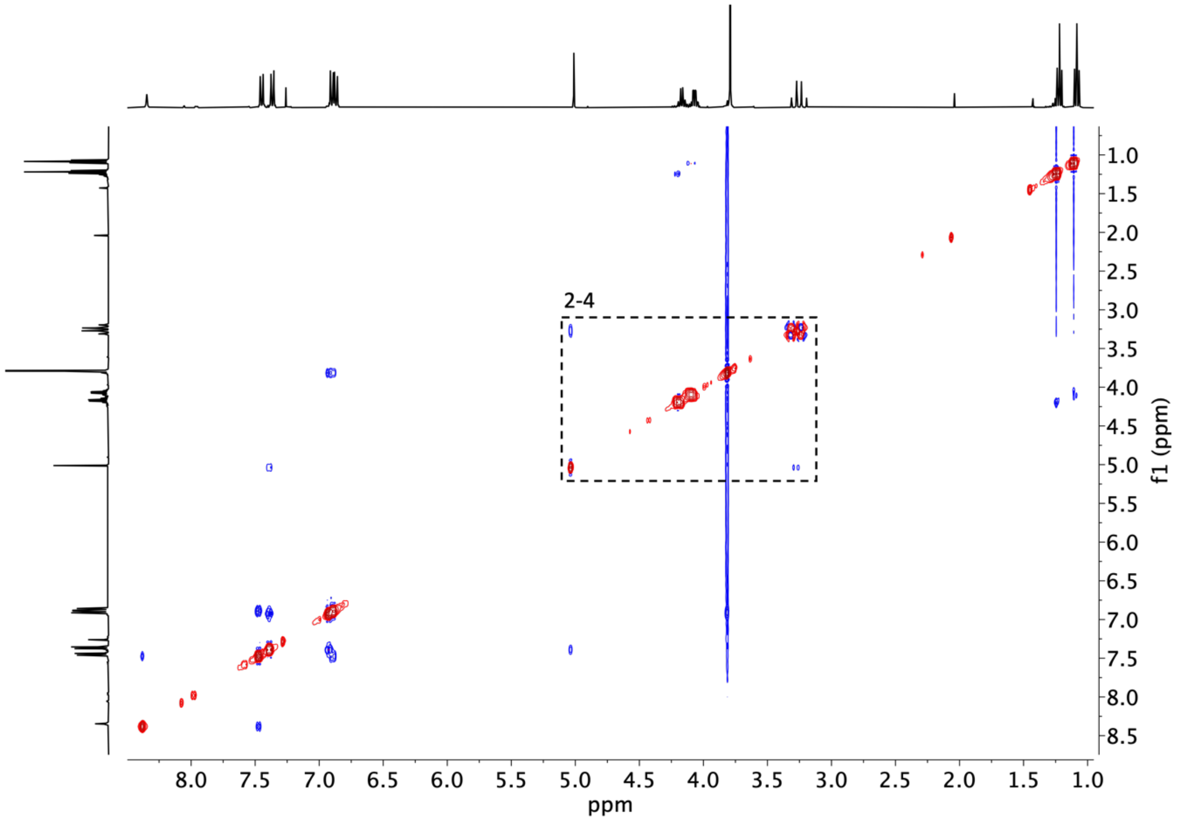

Figure S30. NOESY spectrum of (±)-**2** in $CDCl_3$. Cross-peak between H2 and H4 marked (corroborating relative stereochemistry).

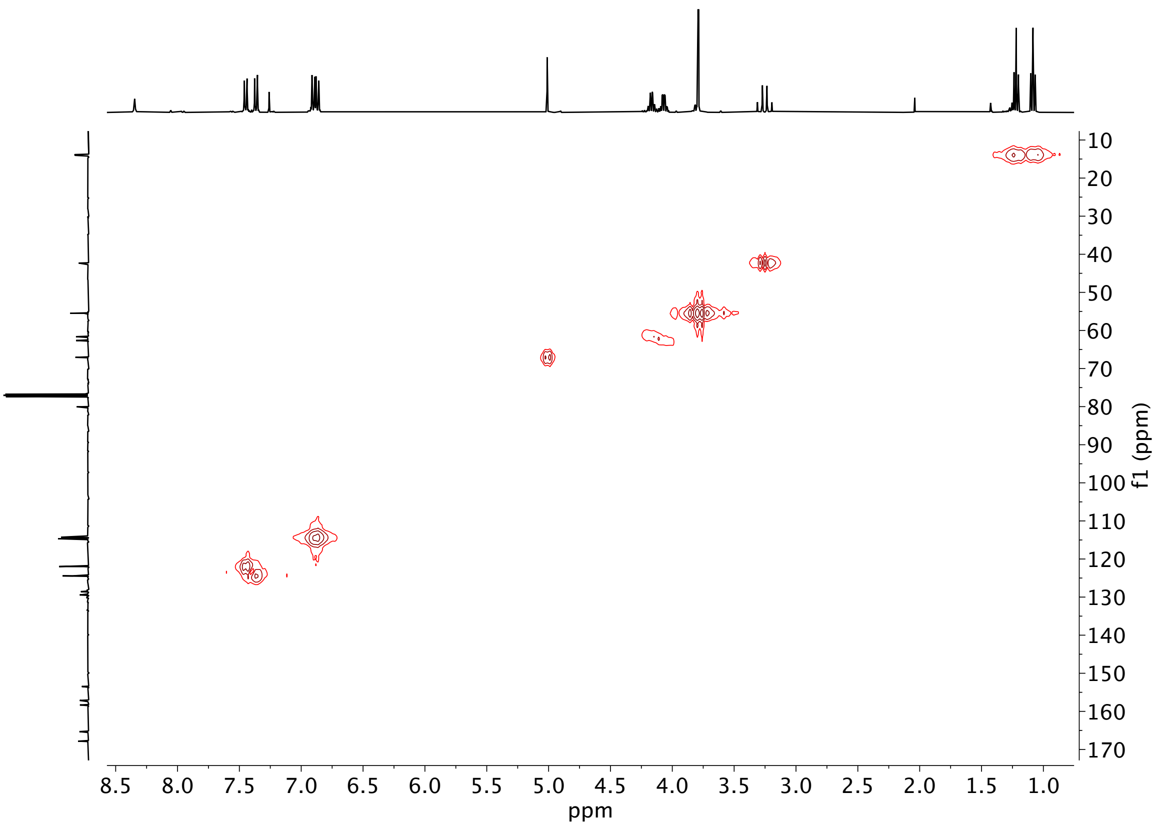

Figure S31. HMQC spectrum of (±)-**2** in $CDCl_3$.

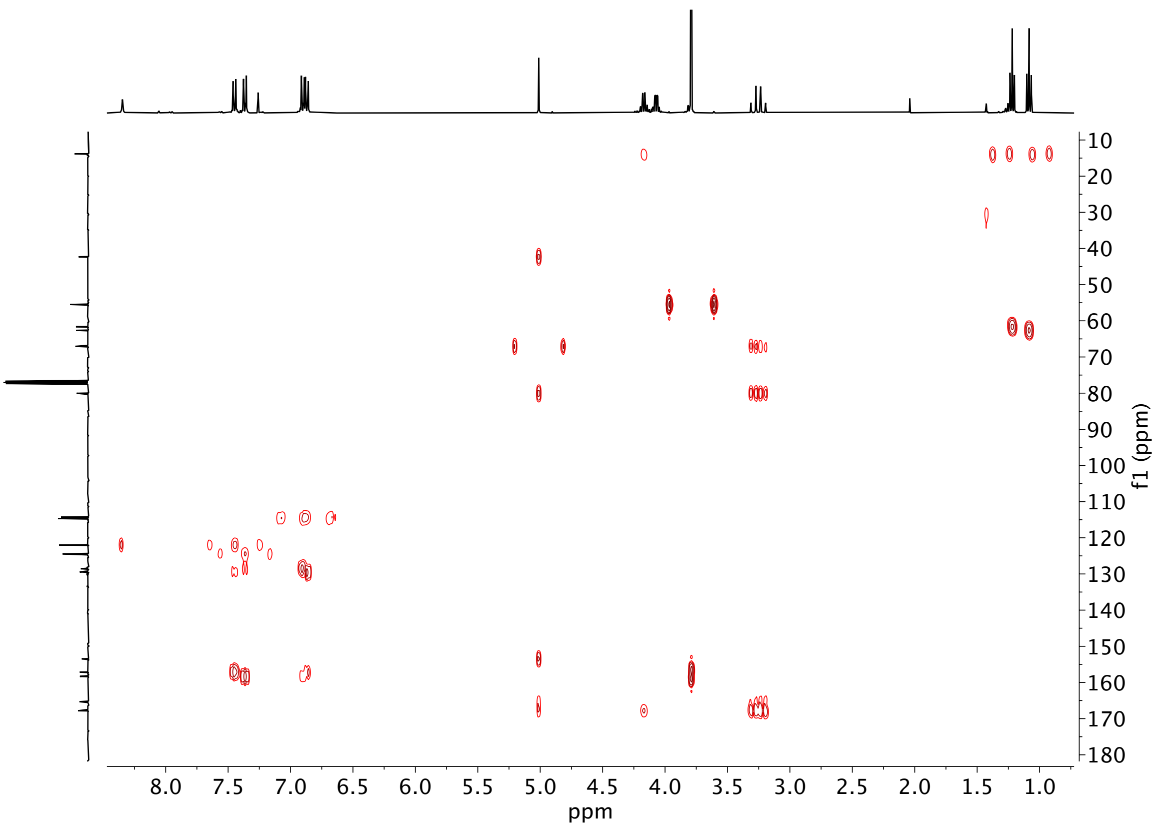

Figure S32. HMBC spectrum of (±)-**2** in $CDCl_3$.

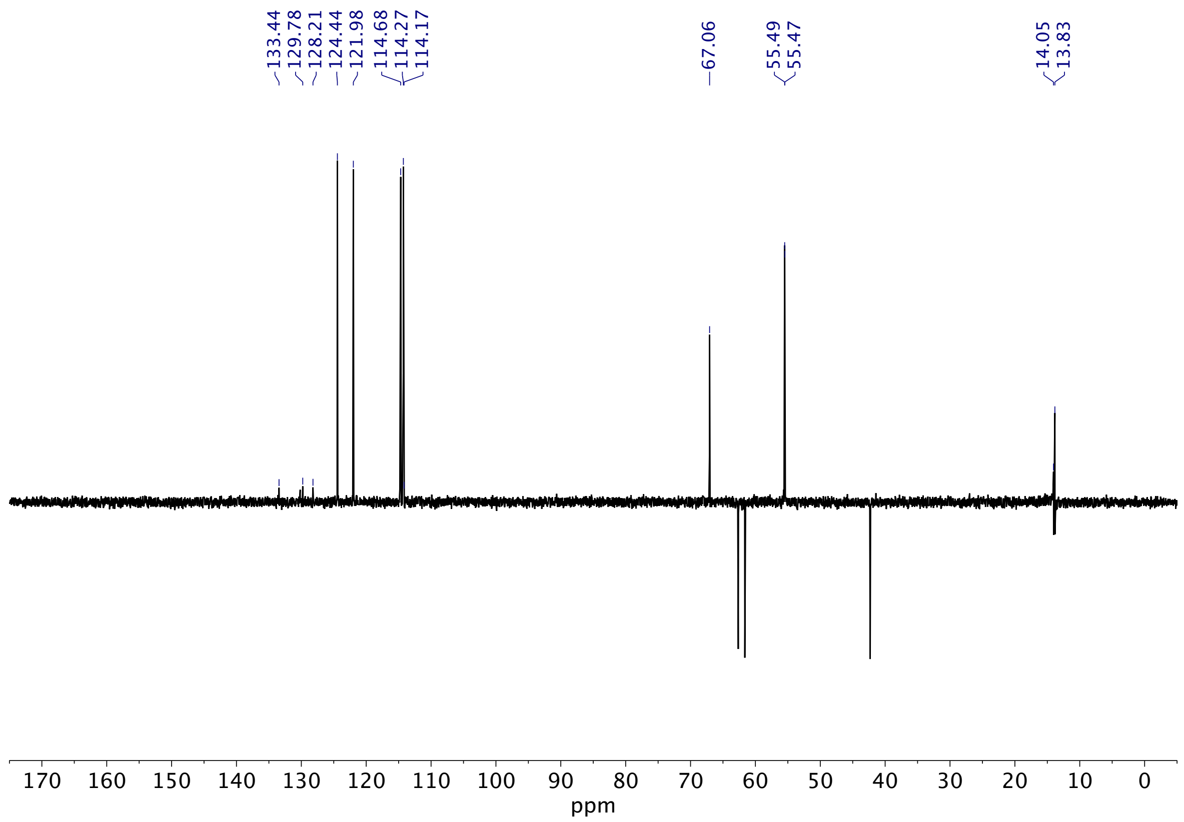

Figure S33. DEPT spectrum of (±)-**2** in $CDCl_3$.

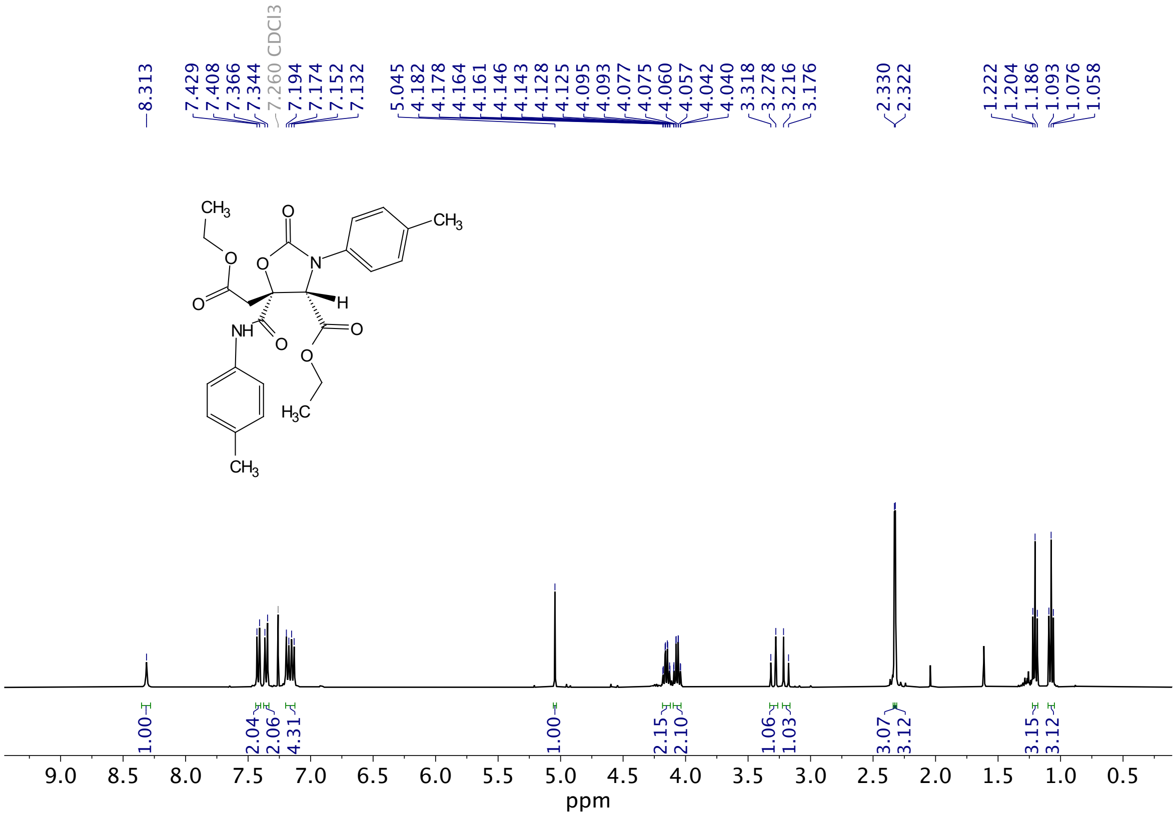

Figure S34. ^{1}H NMR spectrum (400 MHz, $CDCl_3$) of compound (±)-**16**.

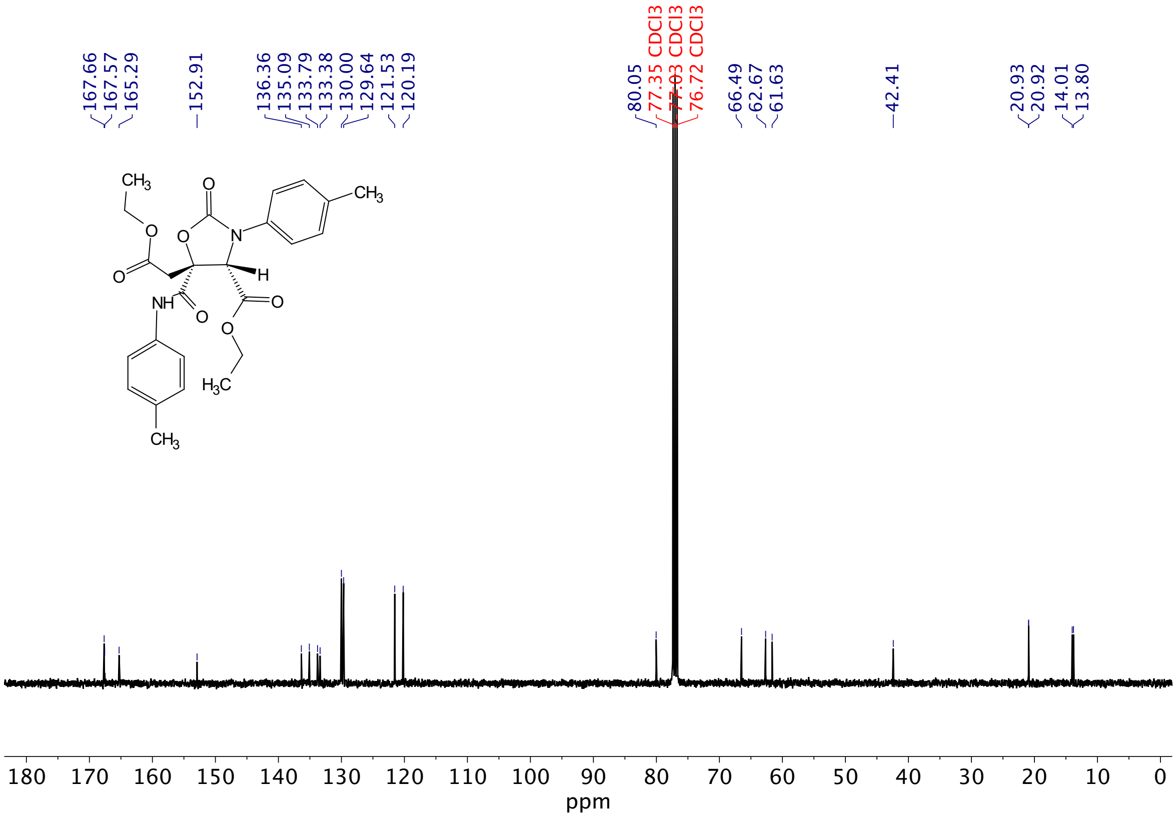

Figure S35. $^{13}C\{^{1}H\}$ NMR spectrum (101 MHz, $CDCl_3$) of compound (±)-**16**.

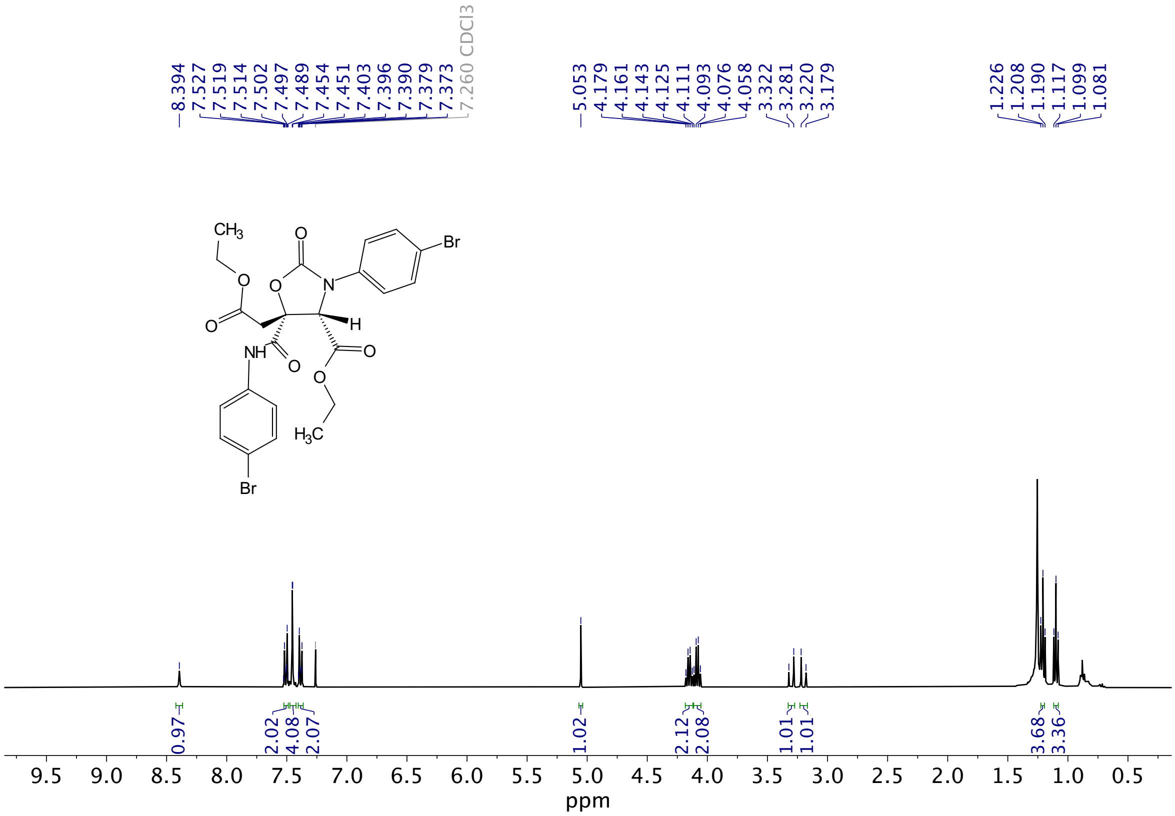

Figure S36. ^{1}H NMR spectrum (400 MHz, $CDCl_3$) of compound (±)-**17**.

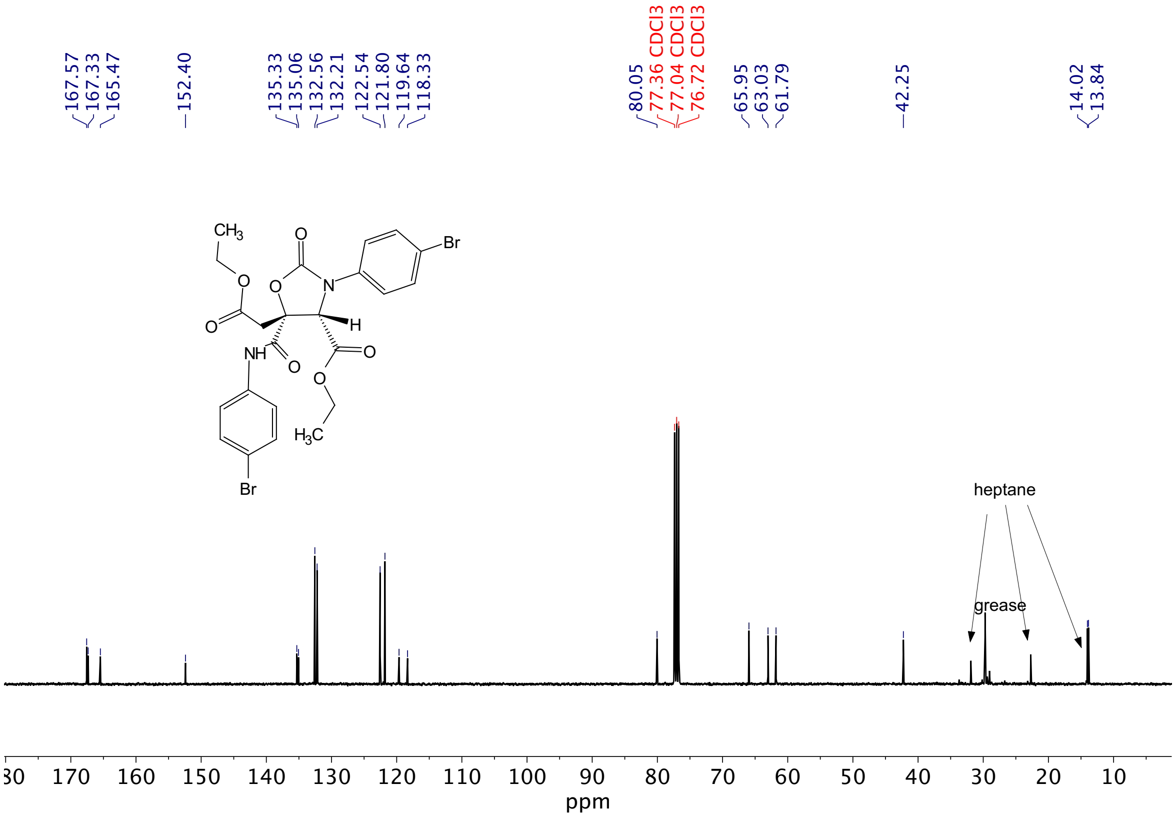

Figure S37. $^{13}C\{^{1}H\}$ NMR spectrum (101 MHz, $CDCl_3$) of compound (±)-**17**.

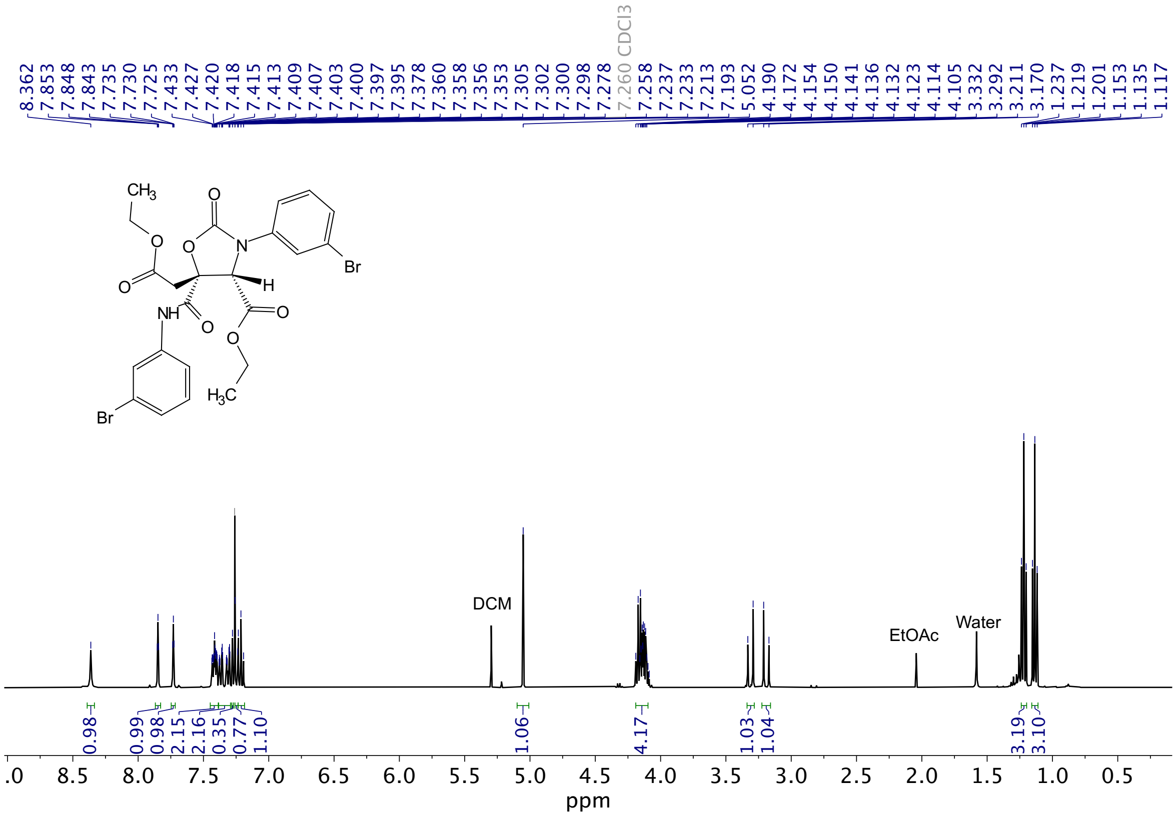

Figure S38. ^{1}H NMR spectrum (400 MHz, $CDCl_3$) of compound (±)-**18**.

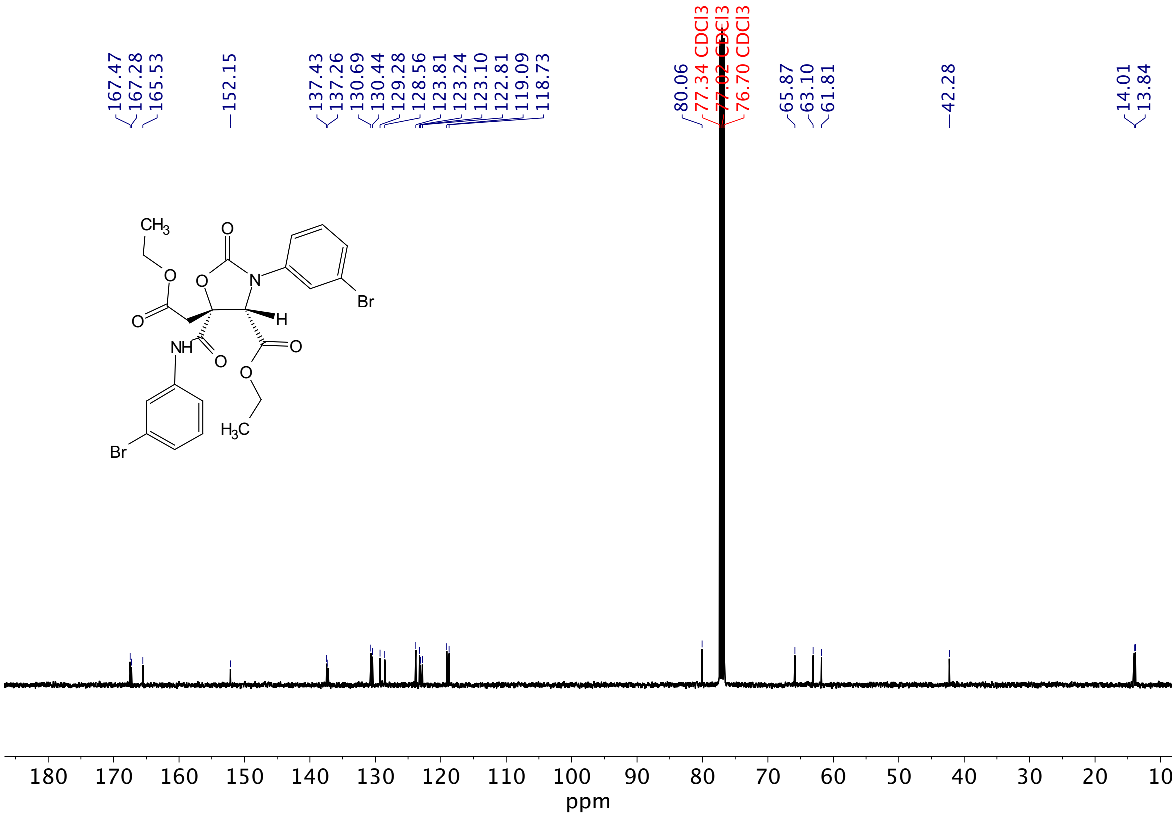

Figure S39. $^{13}C\{^{1}H\}$ NMR spectrum (101 MHz, $CDCl_3$) of compound (±)-**18**.

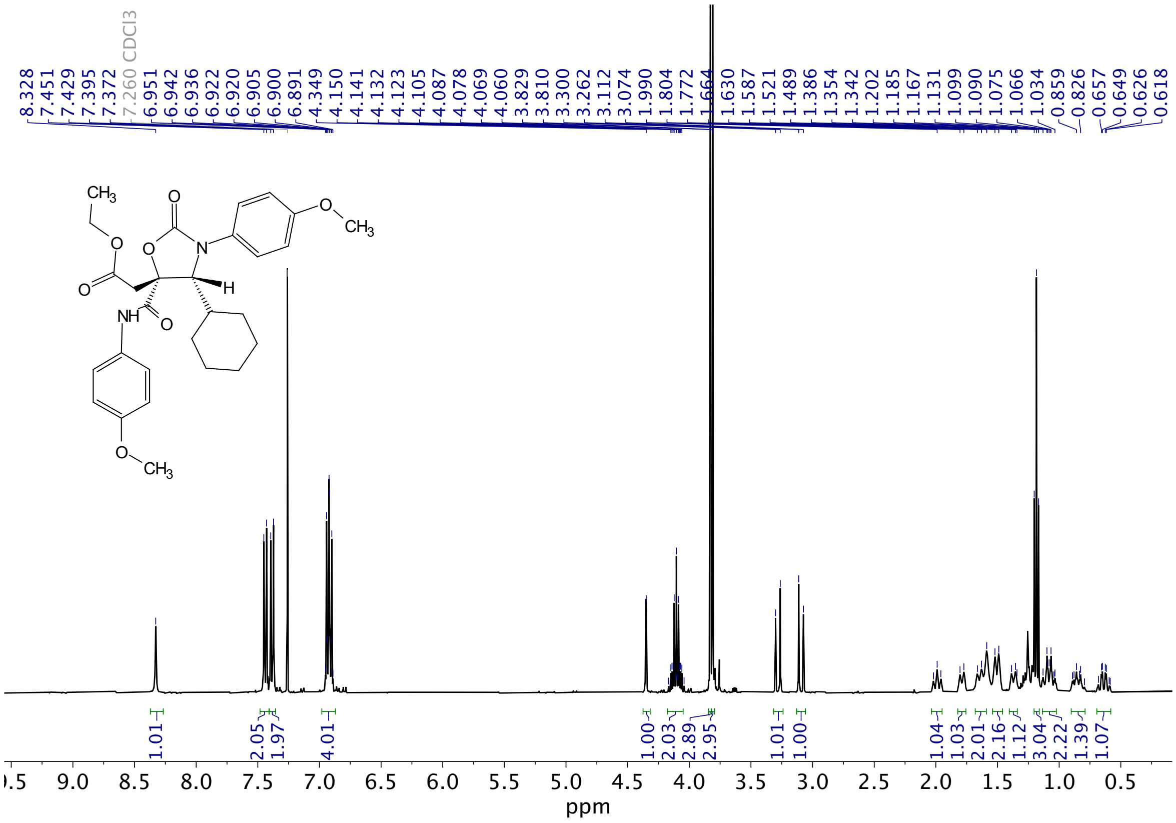

Figure S40. ^{1}H NMR spectrum (400 MHz, $CDCl_3$) of compound (±)-**19**.

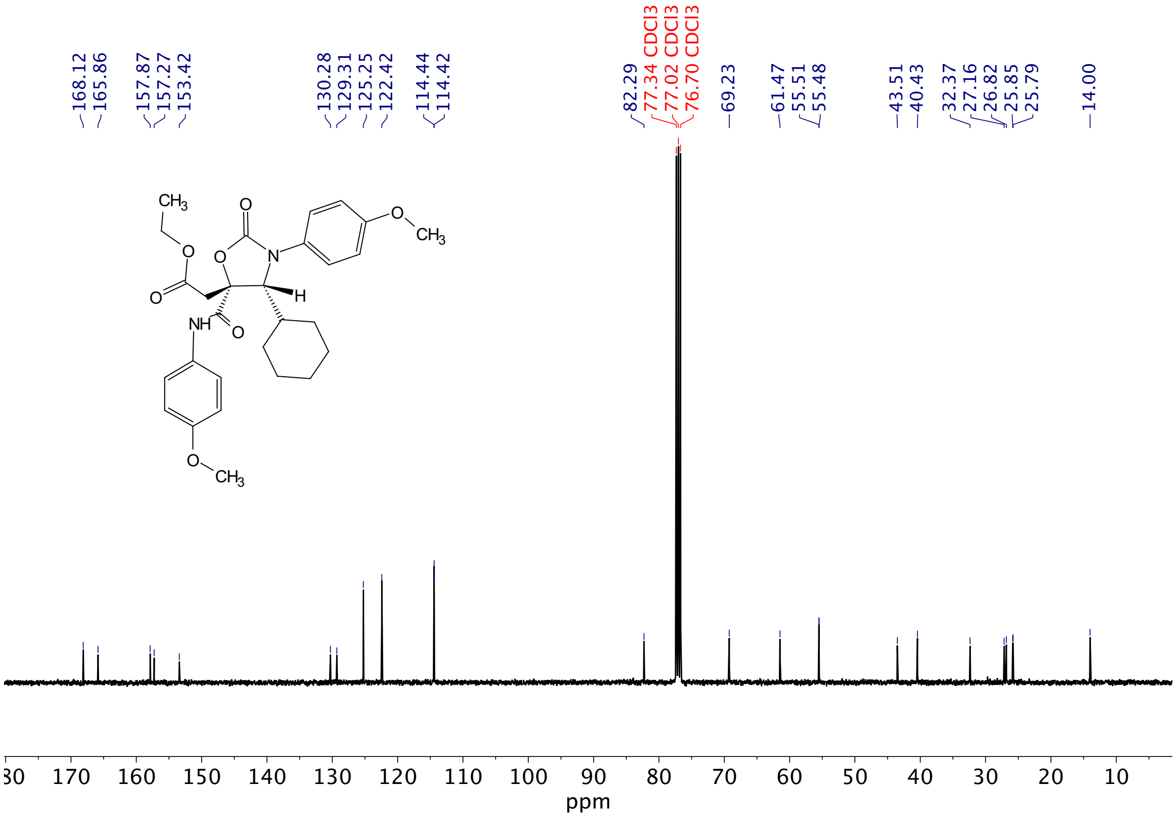

Figure S41. $^{13}C\{^{1}H\}$ NMR spectrum (101 MHz, $CDCl_3$) of compound (±)-**19**.

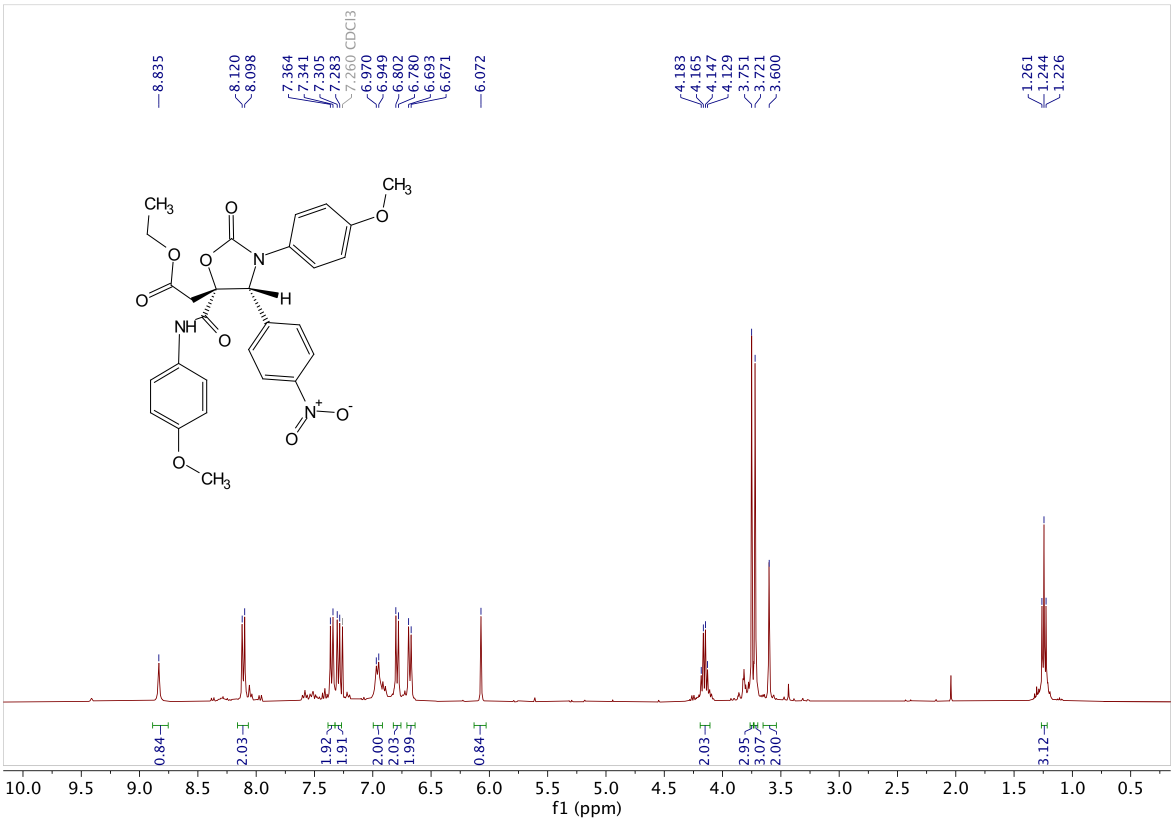

Figure S42. ^{1}H NMR spectrum (400 MHz, $CDCl_3$) of compound (±)-**20**.

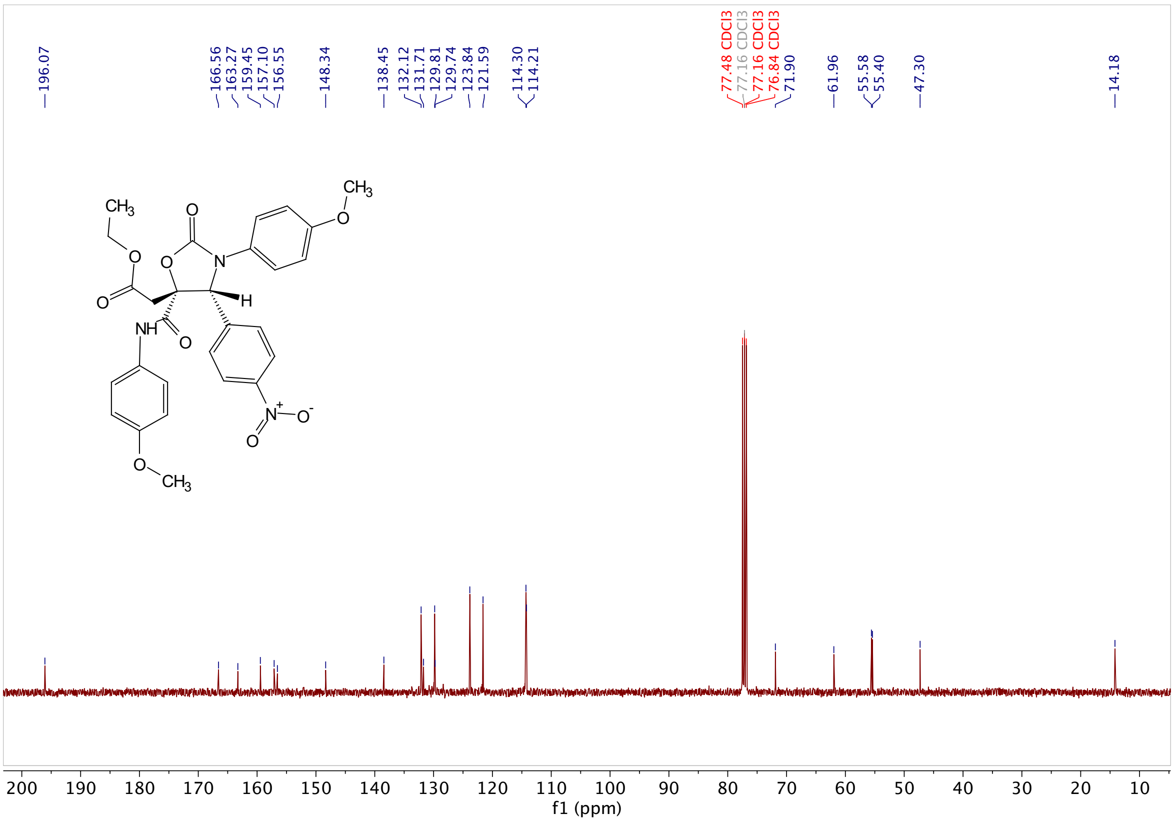

Figure S43. $^{13}C\{^{1}H\}$ NMR spectrum (101 MHz, $CDCl_3$) of compound (±)-**20**.

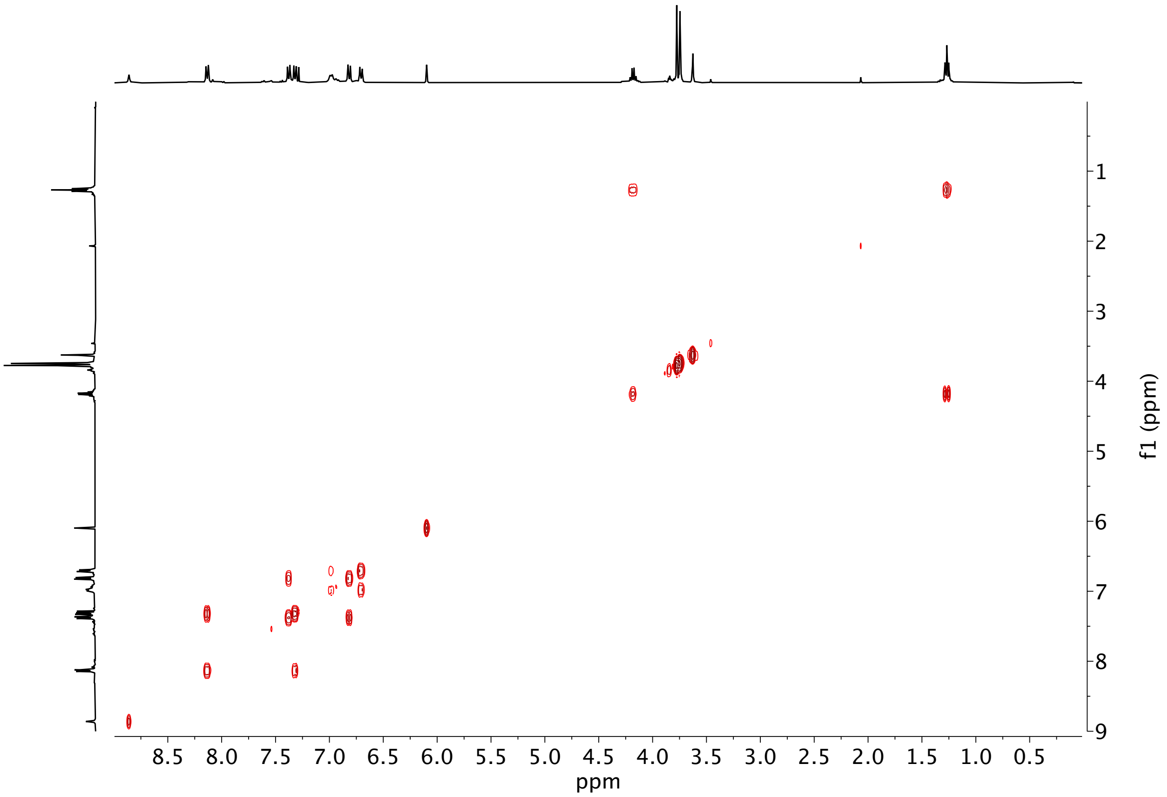

Figure S44. COSY spectrum of compound (±)-**20** in $CDCl_3$.

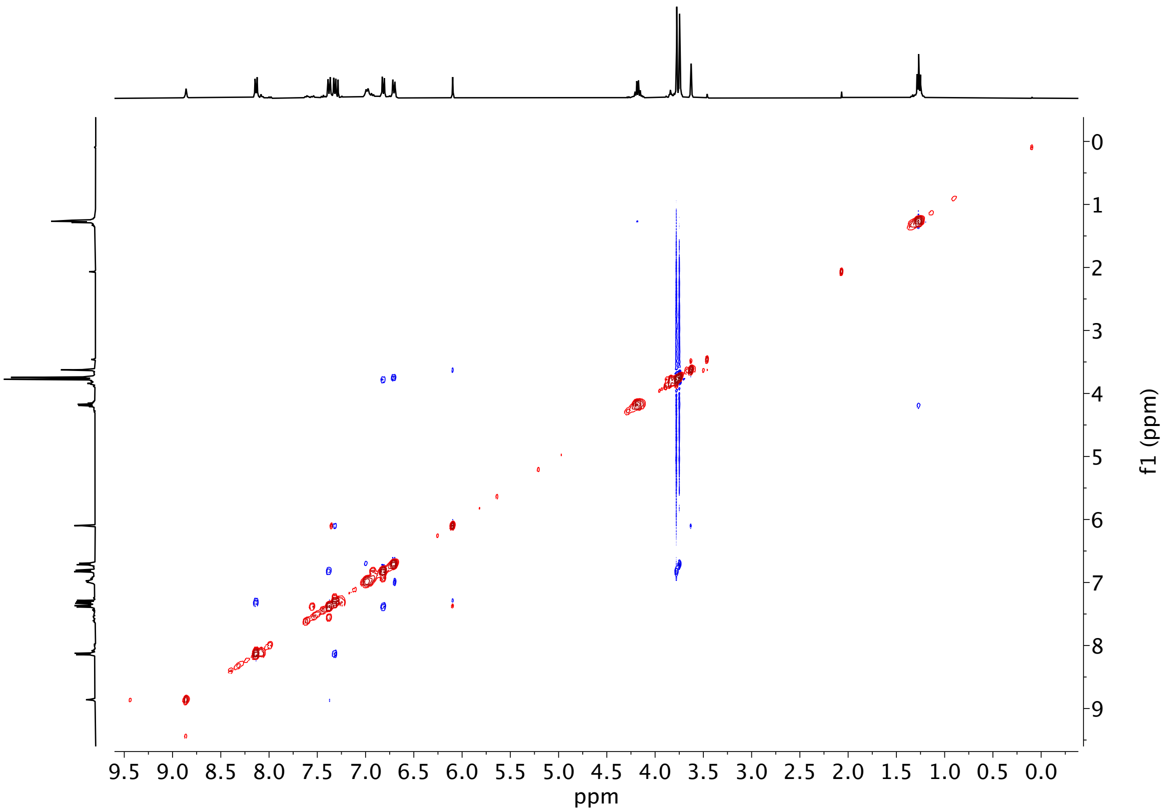

Figure S45. NOESY spectrum of compound (±)-**20** in $CDCl_3$.

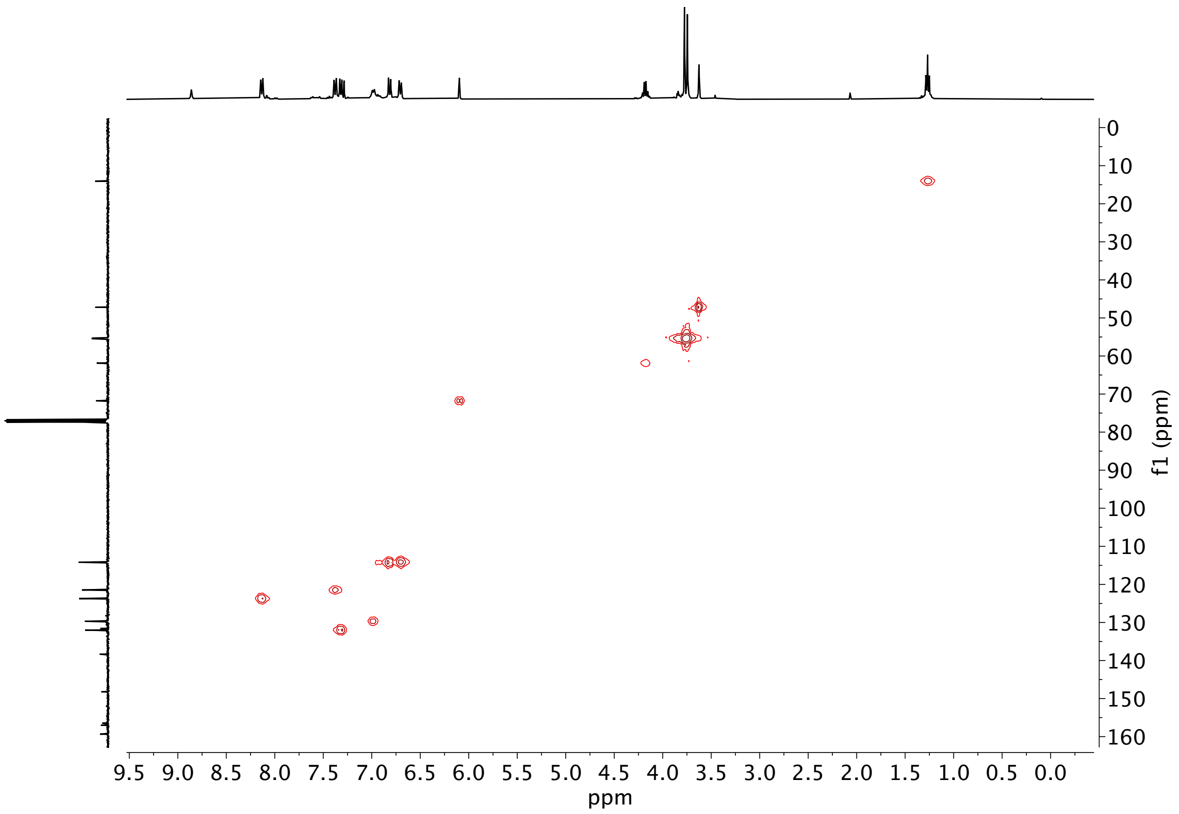

Figure S46. HMQC spectrum of compound (±)-**20** in $CDCl_3$.

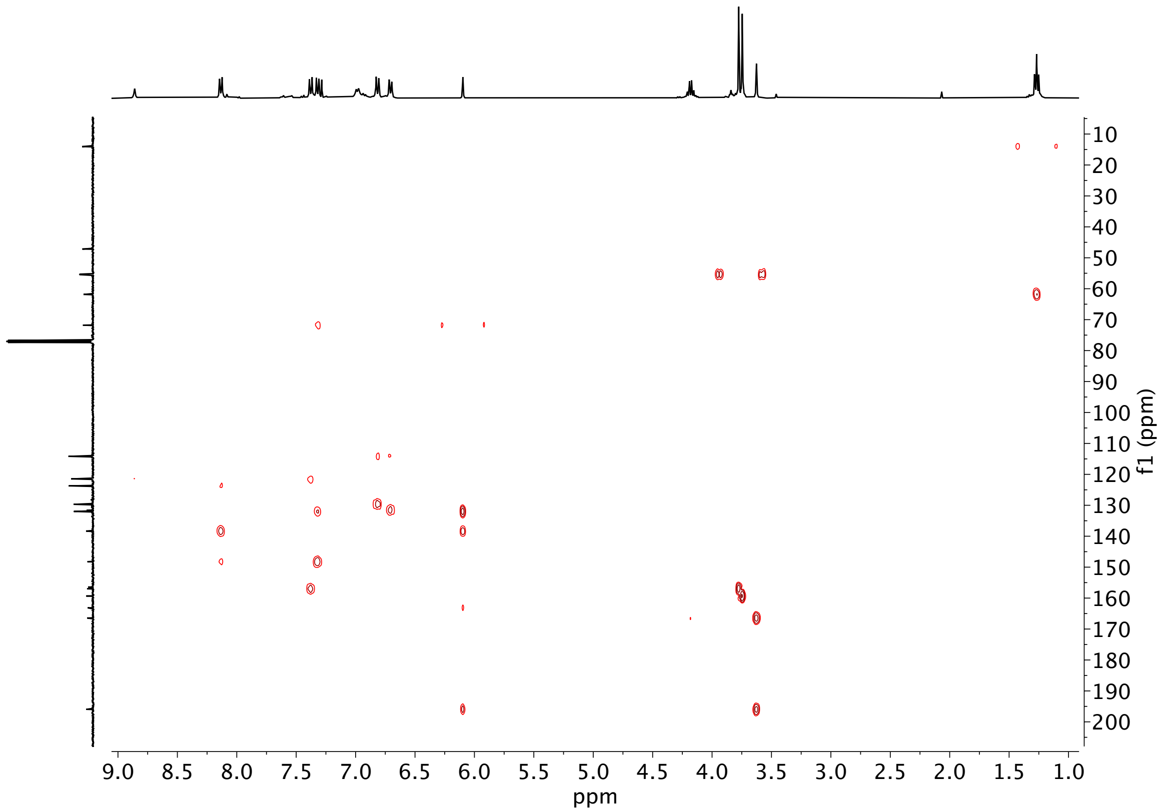

Figure S47. HMBC spectrum of compound (±)-**20** in $CDCl_3$.

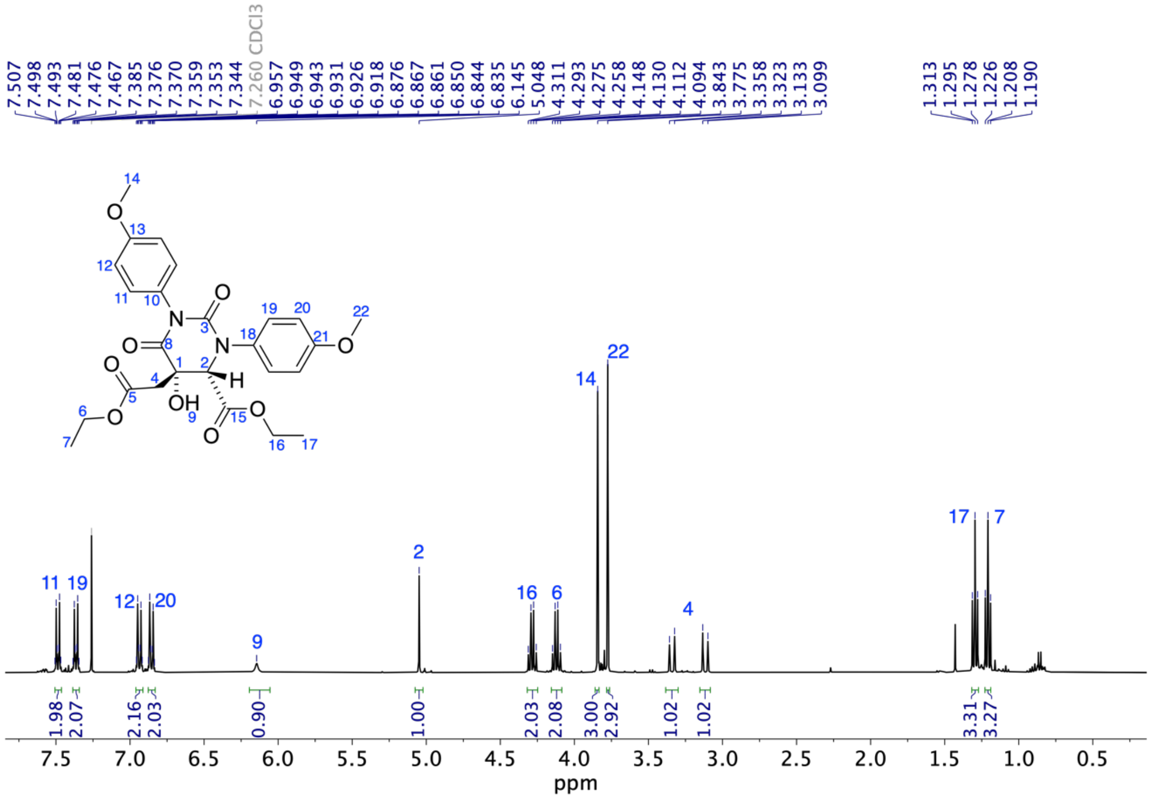

Figure S48. ^{1}H NMR spectrum (400 MHz, $CDCl_3$) of side product to (±)-**3**. Peak assignment is based on the proposed structure of (±)-**3** shown in the figure.

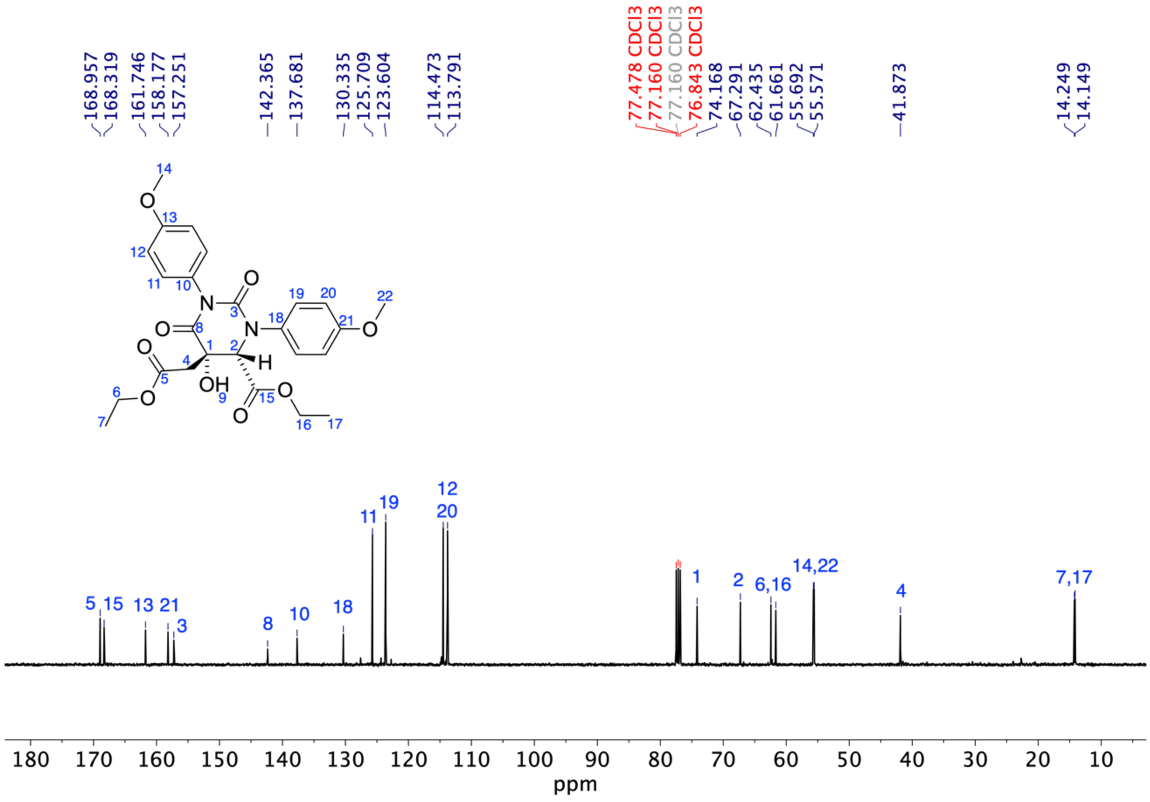

Figure S49. $^{13}C\{^{1}H\}$ NMR spectrum (101 MHz, $CDCl_3$) of side product (±)-**3**. Peak assignment is based on the proposed structure of (±)-**3** shown in the figure.

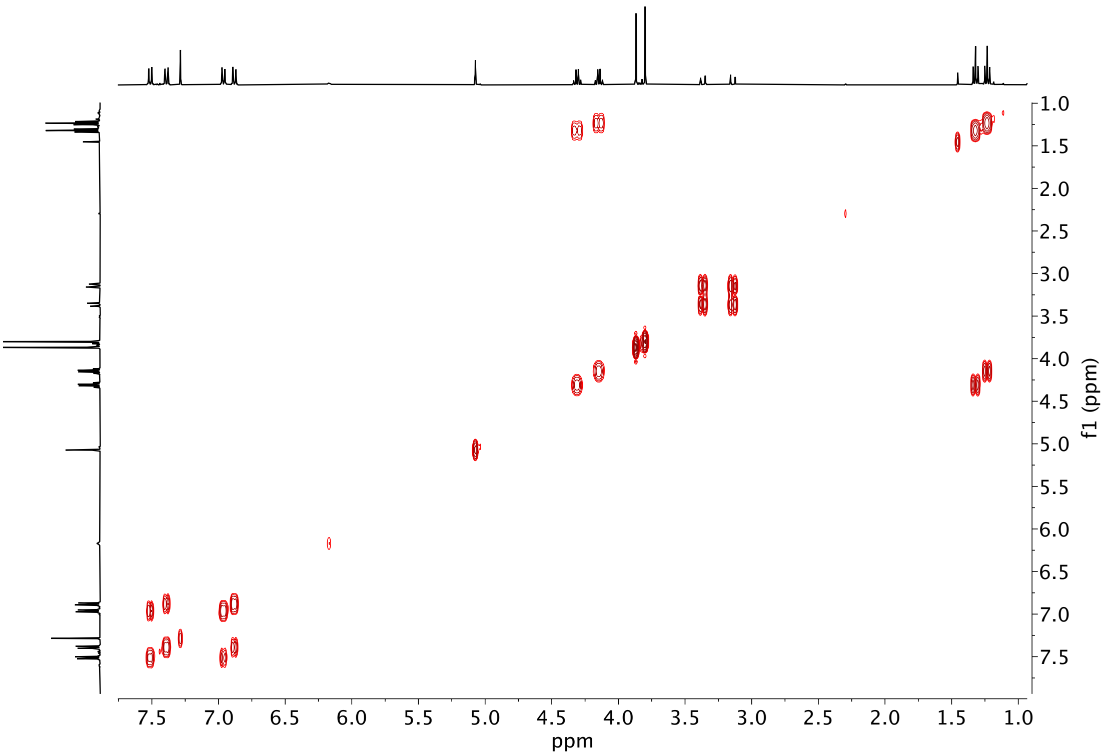

Figure S50. COSY spectrum of side product (±)-**3** in $CDCl_3$.

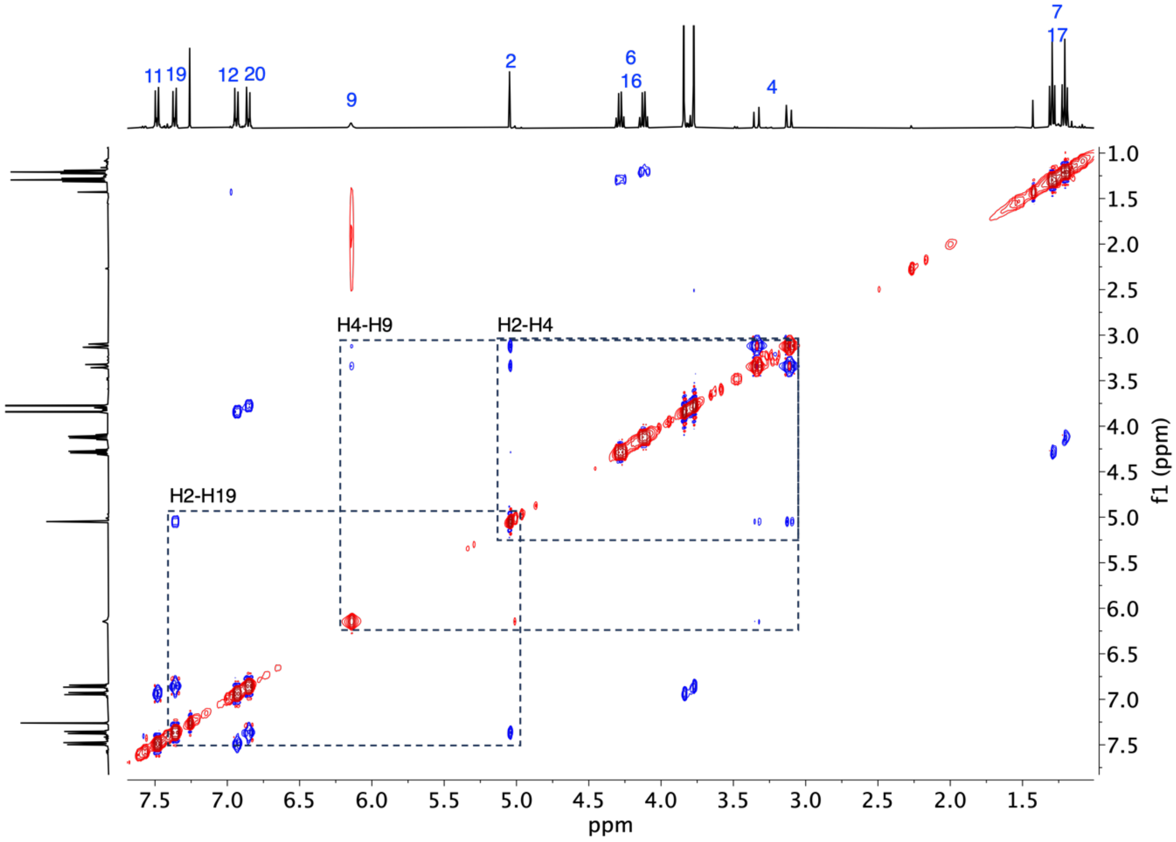

Figure S51. NOESY spectrum of side product (±)-**3** in $CDCl_3$. Cross peaks relevant for assignment of are marked.

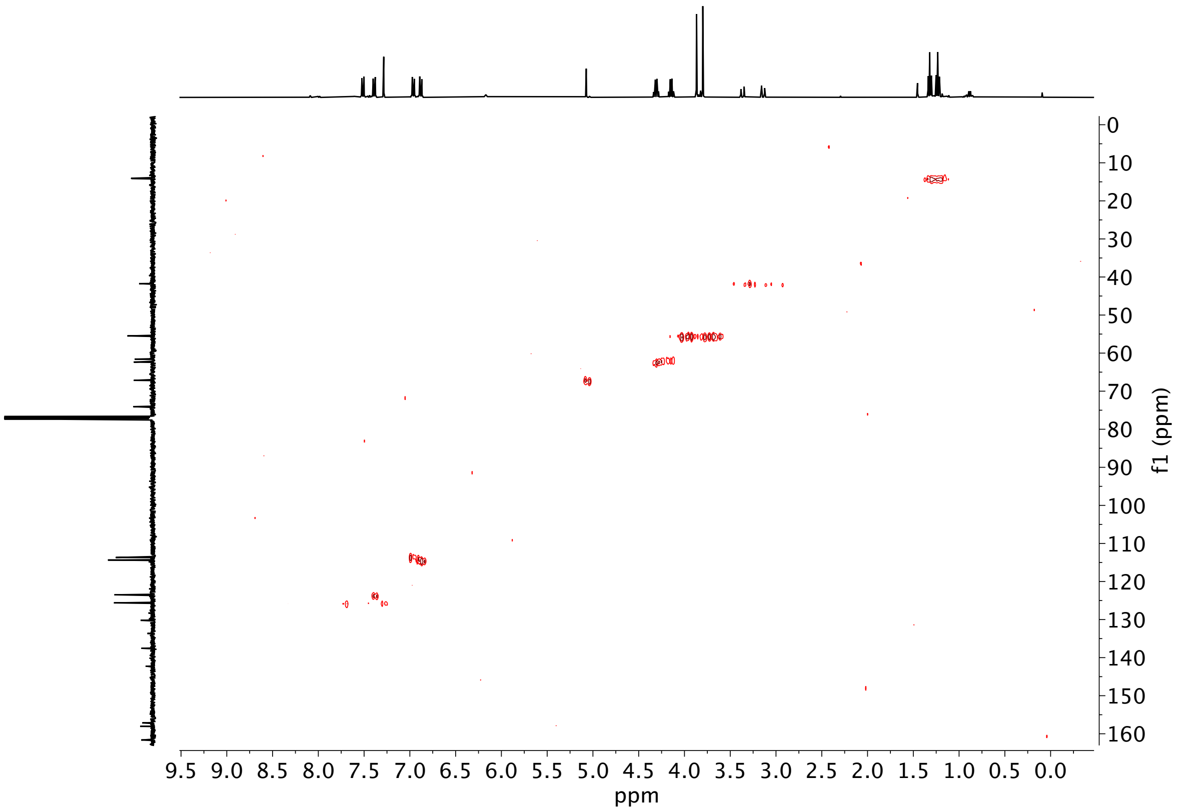

Figure S52. HMQC spectrum of side product (±)-**3** in $CDCl_3$.

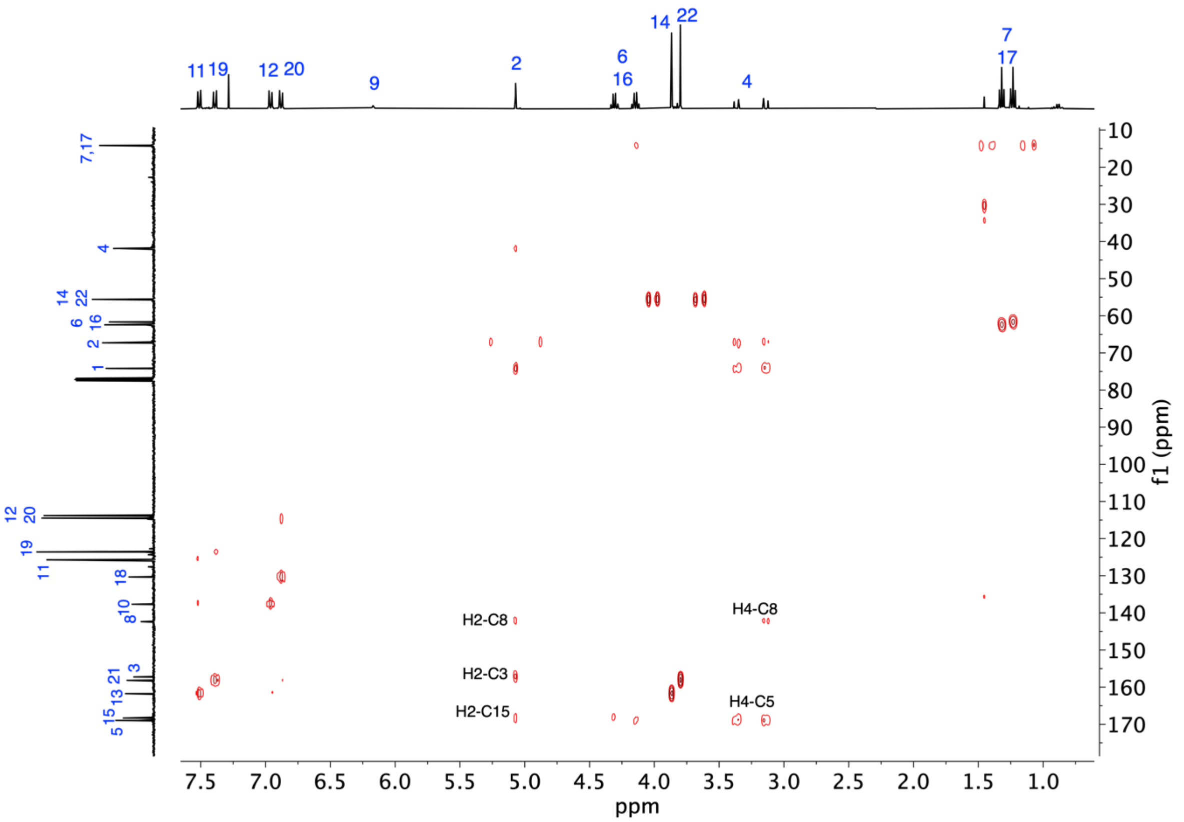

Figure S53. HMBC spectrum of side product (±)-**3** in $CDCl_3$. Cross peaks relevant for assignment of are marked.

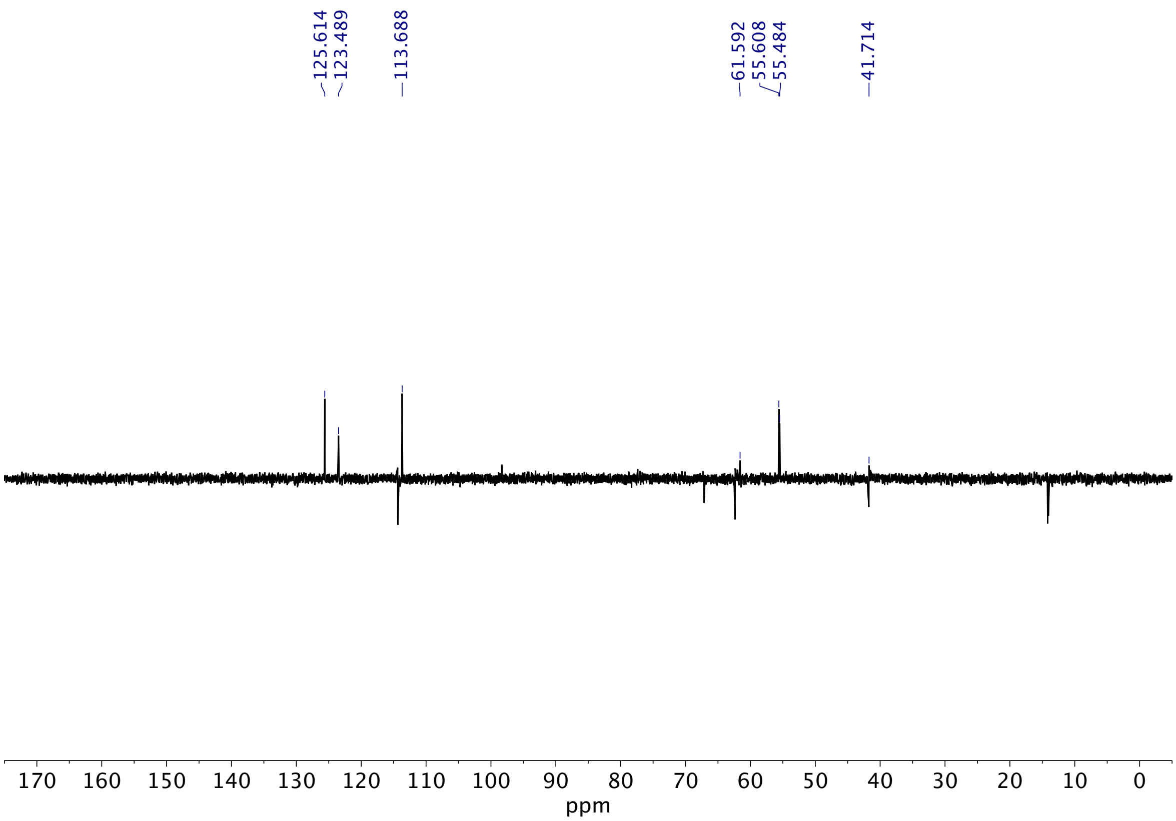

Figure S54. DEPT spectrum of side product (±)-**3** in $CDCl_3$.

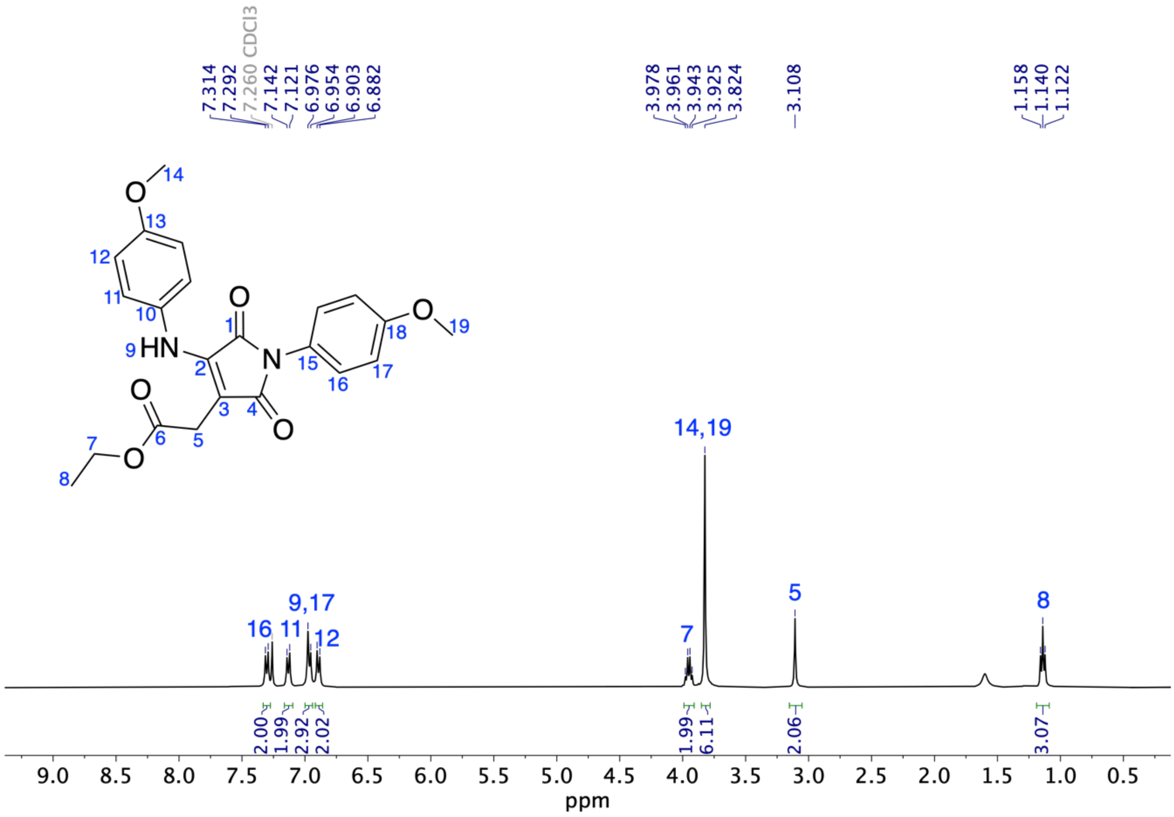

Figure S55. ^{1}H NMR spectrum (400 MHz, $CDCl_3$) of side product **4**.

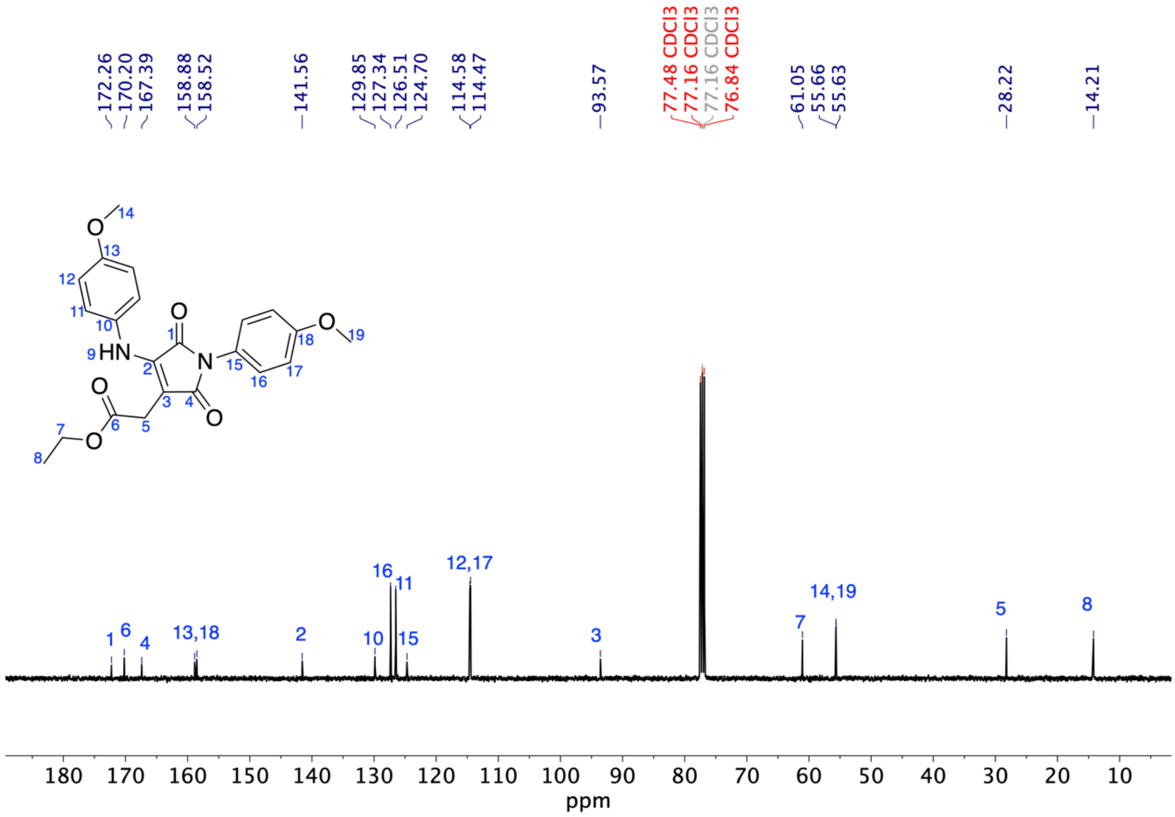

Figure S56. $^{13}C\{^{1}H\}$ NMR spectrum (400 MHz, $CDCl_3$) of side product **4**.

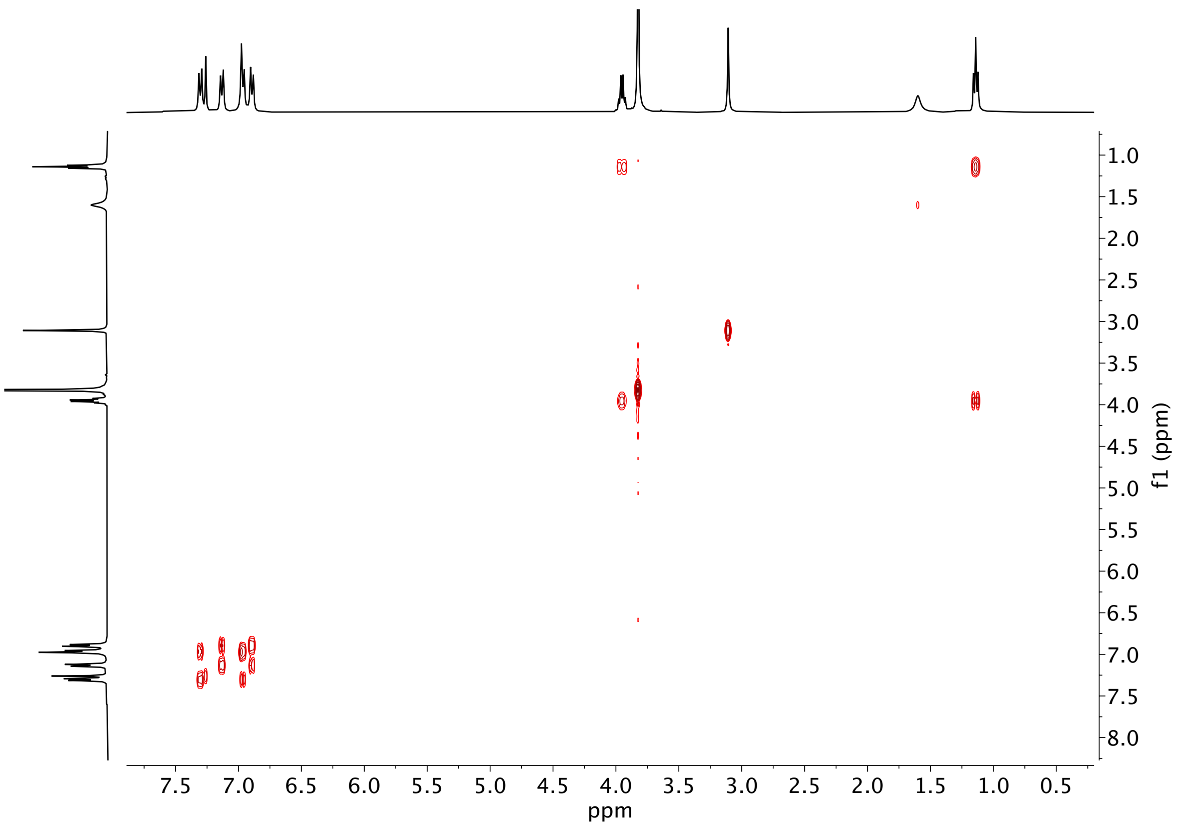

Figure S57. COSY spectrum of side product **4** in $CDCl_3$.

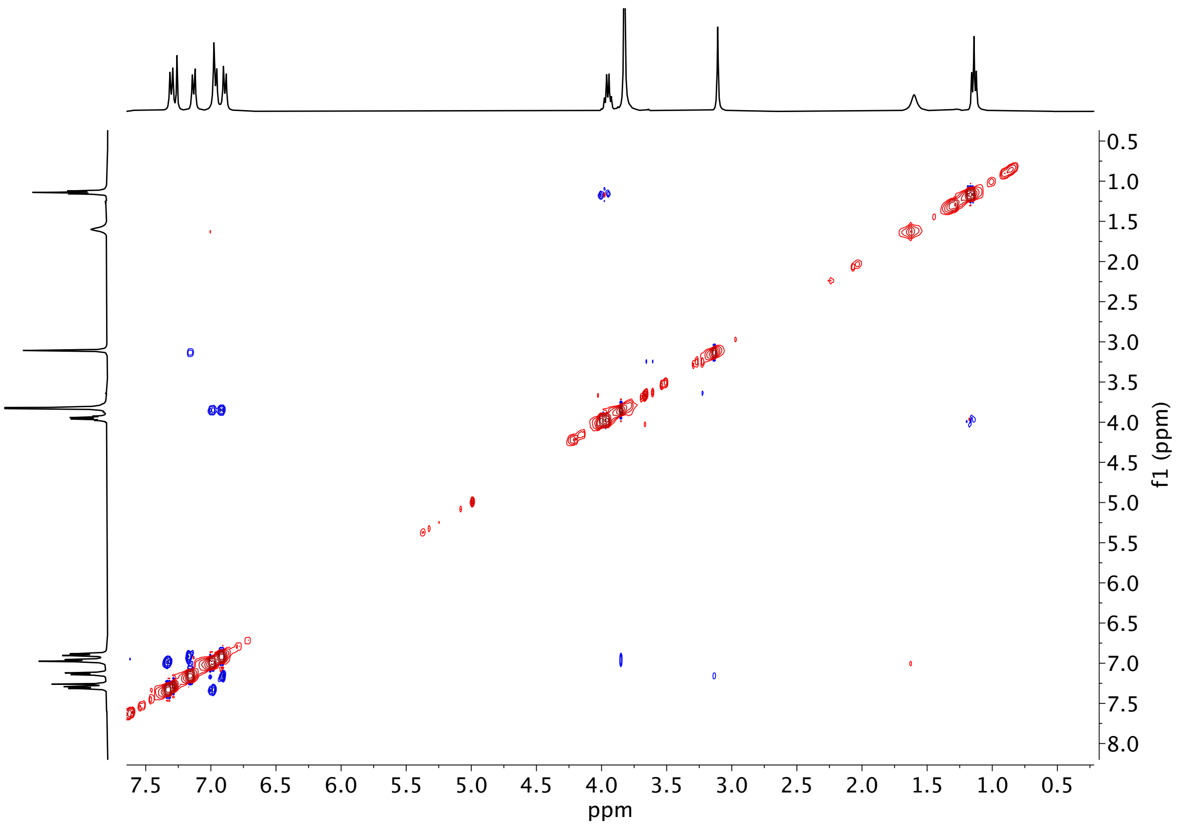

Figure S58. NOESY spectrum of side product **4** in $CDCl_3$.

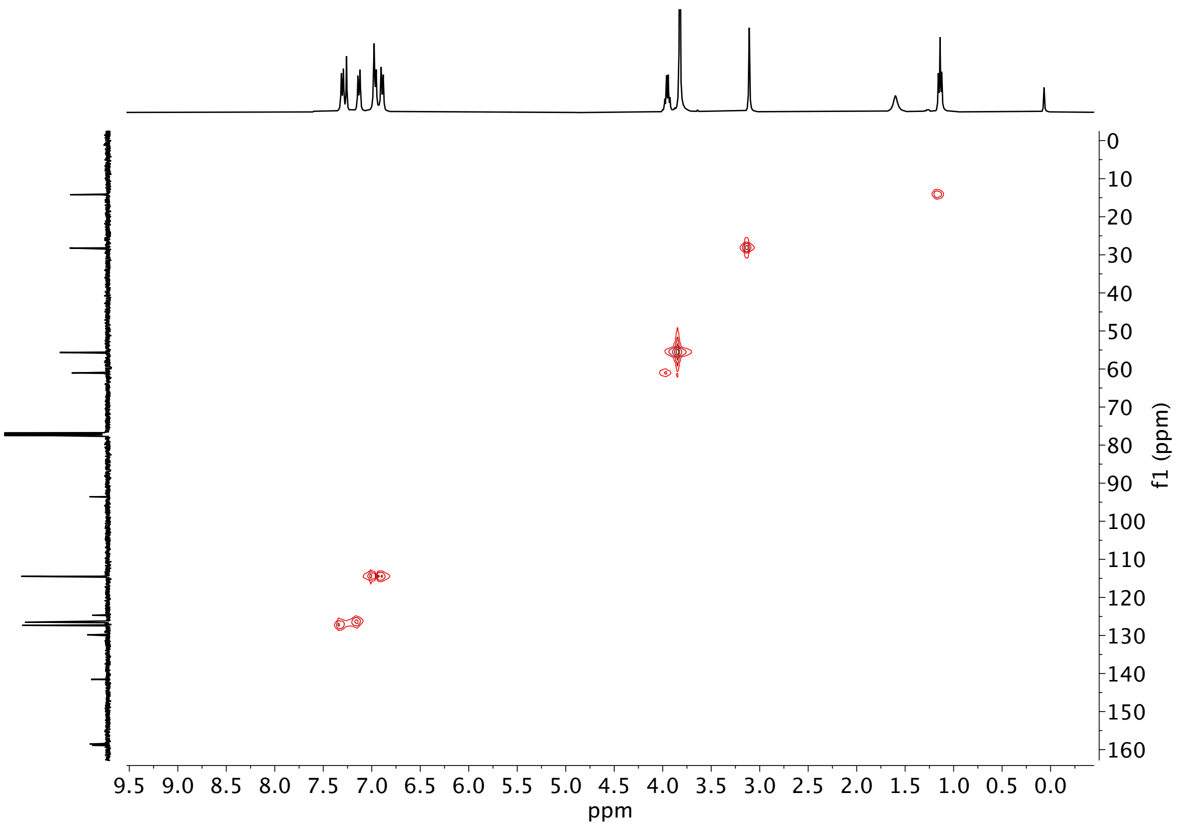

Figure S59. HMQC spectrum of side product **4** in $CDCl_3$.

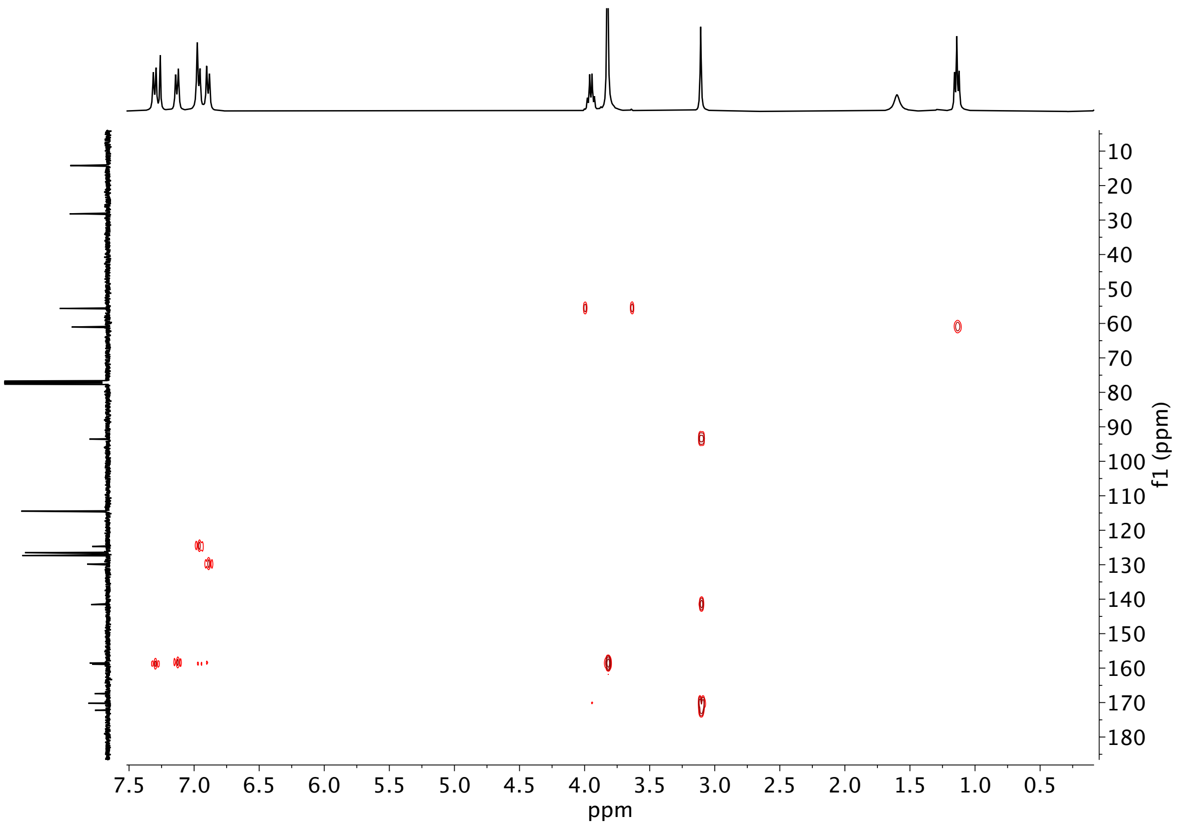

Figure S60. HMBC spectrum of side product **4** in $CDCl_3$.

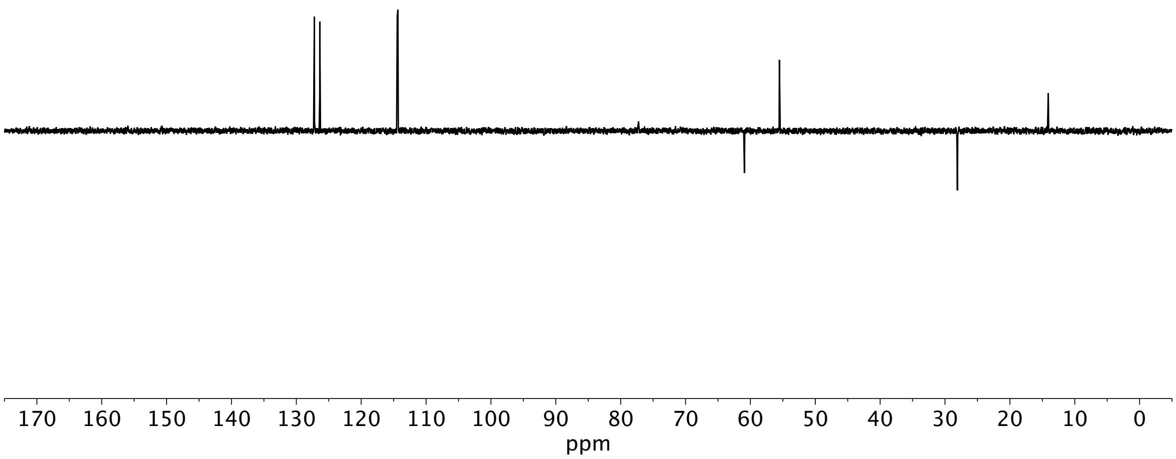

Figure S61. DEPT spectrum of side product **4** in $CDCl_3$.

References

S1. K. Aggarwal, V.; Gültekin, Z.; S. Grainger, R.; Adams, H.; L. Spargo, P. (1R,3R)-2-Methylene-1,3-dithiolane 1,3-Dioxide: A Highly Reactive and Highly Selective Chiral Ketene Equivalent in Cycloaddition Reactions with a Broad Range of Dienes. *J. Chem. Soc., Perkin Trans. 1* **1998,** (17), 2771-2782.

S2. Ferreira de Freitas, R.; Harding, R. J.; Franzoni, I.; Ravichandran, M.; Mann, M. K.; Ouyang, H.; Lautens, M.; Santhakumar, V.; Arrowsmith, C. H.; Schapira, M. Identification and Structure–Activity Relationship of HDAC6 Zinc-Finger Ubiquitin Binding Domain Inhibitors. *J. Med. Chem.* **2018,** *61* (10), 4517-4527.

S3. Li, Y.; Strand, D.; Grimme, S.; Jónsson, S.; Wärnmark, K. The Long-Awaited Synthesis and Self-Assembly of a Small Rigid C3-Symmetric Trilactam. *Chem. Commun.* **2022,** *58* (23), 3751-3754.

S4. del Corte, X.; Maestro, A.; Vicario, J.; Martinez de Marigorta, E.; Palacios, F. Brönsted-Acid-Catalyzed Asymmetric Three-Component Reaction of Amines, Aldehydes, and Pyruvate Derivatives. Enantioselective Synthesis of Highly Functionalized γ-Lactam Derivatives. *Org. Lett.* **2018,** *20* (2), 317-320.

S5. Sheldrick, G. M. A Short History of SHELX. *Acta Cryst.* **2008,** *A64* (1), 112-122.

S6. Sheldrick, G. M. Crystal Structure Refinement with SHELXL. *Acta Cryst.* **2015,** *C71* (1), 3-8.

S7. CrysAlis PRO. Agilent Technologies 2011.

S8. Dolomanov, O. V.; Bourhis, L. J.; Gildea, R. J.; Howard, J. A. K.; Puschmann, H. OLEX2: A Complete Structure Solution, Refinement and Analysis Program. *J. Appl. Crystallogr.* **2009,** *42* (2), 339-341.

S9. Schrödinger Release 2020-3: Jaguar, Schrödinger, LLC, New York, NY, 2020.

S10. Bochevarov, A. D.; Harder, E.; Hughes, T. F.; Greenwood, J. R.; Braden, D. A.; Philipp, D. M.; Rinaldo, D.; Halls, M. D.; Zhang, J.; Friesner, R. A. Jaguar: A High-Performance Quantum Chemistry Software Program with Strengths in Life and Materials Sciences. *Int. J. Quantum Chem* **2013,** *113* (18), 2110-2142.

S11. Zhao, Y.; Truhlar, D. G. The M06 Suite of Density Functionals for Main Group Thermochemistry, Thermochemical Kinetics, Noncovalent Interactions, Excited States, and Transition Elements: Two New Functionals and Systematic Testing of Four M06-Class Functionals and 12 Other Functionals. *Theor. Chem. Acc.* **2008,** *120* (1), 215-241.
